# Multi-omic integration of single-cell data uncovers methylation profiles of super-enhancers in skeletal muscle stem cells

**DOI:** 10.1186/s13072-025-00619-0

**Published:** 2025-08-11

**Authors:** Anyu Zeng, Hailong Liu, Shuling He, Xuming Luo, Zhiqi Zhang, Ming Fu, Baoxi Yu

**Affiliations:** 1 Department of Joint Surgery, First Affiliated Hospital of Sun Yat-sen University, #58 Zhongshan 2nd Road, Guangdong 510080 Guangzhou, P. R. China; 2 Department of Bone and Soft Tissue Surgery, Sun Yat-sen University Cancer Center, Guangzhou, Guangdong 510080 P. R. China; 3 Department of Orthopaedics, Qilu Hospital of Shandong University, Jinan, Shandong 250012 P. R. China; 4 Traditional Chinese Medicine Prevention and Health Care Department, Zhongshan Hospital of Traditional Chinese Medicine Affiliated to Guangzhou University of Chinese Medicine, Zhongshan, Guangdong 528400 P. R. China #3 Kangxin Road,

**Keywords:** Super-enhancer, Methylation reprogramming, Skeletal muscle stem cell, Aging

## Abstract

**Introduction:**

Skeletal muscle stem cells (MuSCs) have strong regenerative abilities, but as we age, their ability to regenerate decreases, leading to a decline in muscle function. Although the methylation reprogramming of super-enhancers (SEs) plays a pivotal role in regulating gene expression associated with the aging process, our understanding of the molecular diversity of stem cells during aging remains limited. This study aimed to identify the methylation profile of SEs in MuSCs and explore potential therapeutic molecular targets associated with aging.

**Methods:**

The ROSE software was employed to identify super enhancers from the ChIP-seq data obtained from the ENCODE database. Additionally, the ALLCools and Methylpy packages were applied to analyze the methylation profile of SEs and to identify differentially methylated regions (DMRs) between aged and control samples using single-cell bisulfite sequencing (scBS-seq) data from the Gene Expression Omnibus (GEO) database. Overlap analysis was used to assess the regions of SEs and DMRs. The target genes and motifs were analyzed using KEGG, GO, and HOMER to identify key biological pathways and functions, followed by validation through snATAC-seq and immunofluorescence techniques.

**Results:**

In conclusion, we conducted a multi-omics and cross-species analysis of MuSCs, creating a detailed methylation profile of SEs during aging. We identified key motifs and genes affected by SE methylation reprogramming, revealing important molecular pathways involved in aging. Notably, further analysis of the key gene PLXND1 revealed a decreasing expression trend in aged MuSCs, which appears to be linked to the hypermethylation of SE Rank 869. This epigenetic alteration is likely to contribute to the dysregulation of the SEMA3 signaling pathway, with profound implications for muscle regeneration in MuSCs during aging.

**Conclusion:**

These findings suggest that epigenetic alterations in the methylation reprogramming of SEs are closely linked to the disruption of transcriptional networks during MuSCs aging. Moreover, our results offer valuable insights into the mechanisms driving SE methylation reprogramming, shedding light on how these epigenetic changes contribute to the molecular processes underlying aging.

**Supplementary Information:**

The online version contains supplementary material available at 10.1186/s13072-025-00619-0.

## Introduction

Super-enhancers (SEs) are highly influential distal DNA cis-regulatory elements characterized by Histone H3 Lysine 27 acetylation (H3K27ac), comprising synergistic clusters of standard enhancers that create robust gene regulatory domains with extensive functional impact [1, 2]. Recent studies have demonstrated that methylation modifications of SEs can influence gene expression and direct cell fate transitions, without changing the nucleotide sequence [3]. Transcription factors (TFs) such as GATA3 and AP-1 have been shown to regulate enhancer reprogramming, contributing to the acquisition of drug resistance in breast cancer [4]. Furthermore, FOXA1 is instrumental in driving enhancer activation, thereby enhancing the metastatic potential of pancreatic ductal adenocarcinoma [5]. In neuroblastoma, treatment with all-trans retinoic acid (ATRA) induces specific methylation changes within SEs, facilitating a shift from a malignant to a differentiated cell state [6]. Additionally, Song et al. demonstrated that dynamic, allele-specific DNA methylation at SEs leads to cell-to-cell heterogeneity and influences cellular differentiation, with these processes observed both in vitro and in vivo [7]. SE-mediated non-mutational epigenetic reprogramming represents a highly intricate and tightly regulated cellular process, where SE methylation plays a central role in modulating gene expression without altering the underlying genetic sequence. This form of epigenetic modulation not only highlights the dynamic influence of SE methylation on cellular plasticity and fate decisions but also holds significant potential for therapeutic applications.

In recent years, aging-induced sarcopenia has attracted considerable attention due to its profound impact on health, as skeletal muscle is the largest tissue in the human body and a key regulator of overall energy expenditure [8]. Studies utilizing multi-omics single-cell sequencing of skeletal muscle have revealed significant epigenetic instability in skeletal muscle stem cells (MuSCs) associated with aging, which drives alterations in cell identity and function [9, 10]. These alterations in MuSCs are thought to contribute to the decline in muscle regeneration and function observed in aged individuals. Specifically, the reprogramming of SE methylation has emerged as a critical mechanism in modulating cellular plasticity and identity. Despite the growing recognition of SE-mediated reprogramming’s potential to influence cell fate, the molecular mechanisms underlying these changes remain poorly understood. By precisely regulating cellular responses, SE methylation reprogramming offers a promising strategy for enhancing the efficacy of targeted therapies, presenting an opportunity for the development of personalized and more effective therapeutic interventions in age-related conditions such as sarcopenia.

Consequently, this project aims to investigate the dynamic reprogramming of SE methylation in MuSCs, with the objective of elucidating the molecular mechanisms that govern cell identity and function in this context. Specifically, we aim to construct a comprehensive atlas of SE methylation reprogramming in MuSCs from mice, and to explore key genes and regulatory motifs through single-nucleus ATAC sequencing (snATAC-seq) of human skeletal muscle. This integrated approach provides a deeper understanding of SE methylation dynamics and their role in aging, enabling comparative insights across species.

## Materials and methods

**Fig. 1:**
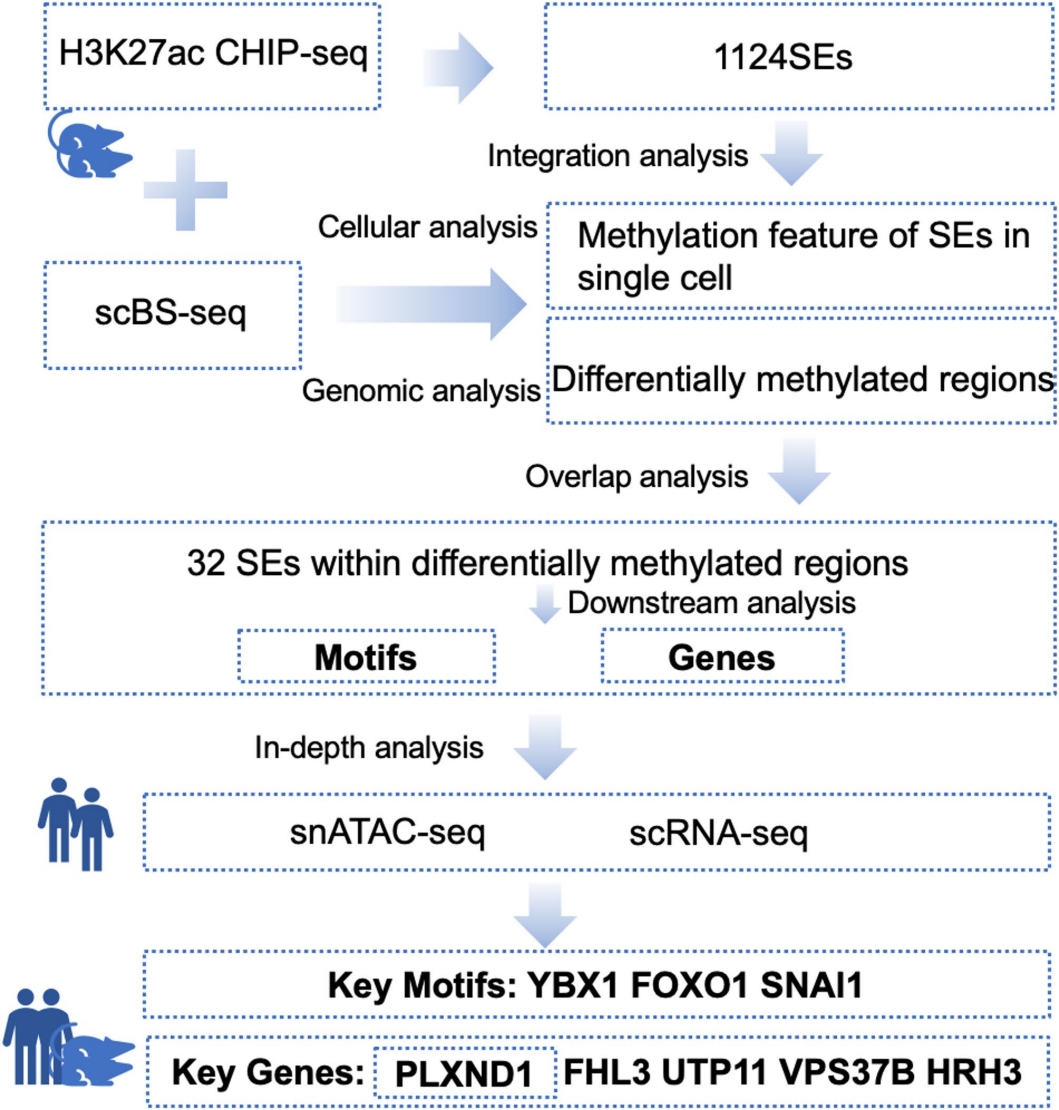
Research methodology flow diagram. Data were sourced from the ENCODE and GEO databases, as well as the China National GeneBank (CNGB). Using the ROSE pipeline, a total of 1,124 SEs in mesenchymal stem cells (MSCs) were identified, and their associated target genes were determined. DNA methylation analysis was performed at the single-cell level in MuSCs using scBS-seq, revealing the methylation reprogramming profile of SEs. Additionally, 1,663 differentially methylated regions were identified by comparing young and aged samples at the genomic level. Overlap analysis further identified 32 SEs exhibiting significant methylation reprogramming. Subsequent investigation of these SEs led to the identification of key regulatory motifs and target genes. These motifs and genes were further analyzed using snATAC-seq, scRNA-seq, and immunofluorescence, allowing for deeper exploration of key regulatory elements

The flowchart of this research is shown in Fig. 1.

### Data acquisition from public databases

H3K27ac ChIP-seq data (accession ID: ENCSR000CCL) and its corresponding control data (accession ID: ENCSR000CAS) were retrieved from the ENCODE database (https://www.encodeproject.org). Single-cell bisulfite sequencing (scBS-seq) DNA methylation data were acquired from the Gene Expression Omnibus (GEO, https://www.ncbi.nlm.nih.gov/geo, GSE121436), generated using the Illumina HiSeq 2500 platform for mus musculus [11]. This dataset includes samples from 3 young mice (2 months old) and 3 aged mice (24 months old). In brief, the isolation of skeletal muscle stem cells and process are as follow:1) Tibialis anterior muscles were dissected from mice, minced, and enzymatically digested at 37 °C in DMEM containing 0.08% collagenase D, 0.1% trypsin, and 10 µg/mL DNase I. Digestion was repeated three times to ensure complete dissociation. Supernatants were collected in fetal bovine serum, filtered through a 70 μm cell strainer, and centrifuged at 515 × g for 15 min at 4 °C. Cell pellets were resuspended in freezing medium (10% DMSO in fetal calf serum) and stored in liquid nitrogen until use. 2) Genomic DNA lysates were processed following a previously published single-cell bisulfite sequencing protocol [12]. Single-cell genomic DNA was purified using AMPure XP beads (Beckman Coulter) and subjected to bisulfite conversion and purification using the EZ-Direct kit (Zymo Research). First-strand synthesis was performed using Klenow exo- polymerase (Enzymatics) and a primer containing the Illumina Read 1 sequence followed by a 3′ random hexamer. This reaction was repeated four additional times for pre-amplification. Following purification, second-strand synthesis was carried out using a primer containing the Illumina Read 2 sequence. Final library amplification and indexing were subsequently performed. Processed libraries were aligned and analyzed using Bismark [13].

Additionally, snATAC-seq and scRNA-seq data were obtained from the Human Muscle Ageing Cell Atlas database (https://db.cngb.org/cdcp/hlma/) [9], associated with the project (CNP0004394, CNP0004395, CNP0004494, and CNP0004495) from the China National GeneBank (CNGB) Nucleotide Sequence Archive.

### Sample collection from human and mouse muscle tissues

Muscle samples were collected specifically for this study from three aged patients (1 male and 2 females, average age 70) undergoing arthroplasty, and from three young male patients (average age 23) undergoing arthroscopic surgery for anterior cruciate ligament (ACL) injuries. Additionally, six male C57BL/6J wild-type mice were obtained from GemPharmatech Co., Ltd. (Jiangsu, China) and assigned to two age groups: three mice aged 8 weeks (young group) and three aged 12 months (aged group). All human and animal samples were newly collected for the present study. Ethical approval for human sample collection was obtained from the Clinical Research Ethics Committee of the First Affiliated Hospital of Sun Yat-sen University (IRB: 2021 − 334), and informed consent was obtained from all participants. Animal experiments were approved by the Institutional Animal Care and Use Committee (IACUC) of Sun Yat-sen University (SYSU-IACUC-2021-000690).

### Identified SEs and function annotation analysis

Binary alignment files in BAM format for MSC H3K27ac ChIP-seq, aligned to the Mmu10 reference genome, were primarily sourced from the ENCODE database. Enhancer and SE identification was performed using the ROSE software in a Python3 environment. During SE identification, enhancers were ranked based on H3K27ac signal intensity, excluding H3K27ac ChIP-seq peaks located within ± 2500 bp of transcription start sites (TSS). Enhancers within a 12,500 bp proximity were stitched together. SEs were subsequently identified by determining a cut-off value at the geometric inflection point. SE features were characterized using the ChIPseeker [14] package (Version 1.40.0). SE-associated target genes were predicted using algorithms developed by Young’s laboratory [15, 16] (Young Lab website: http://younglab.wi.mit.edu/super_enhancer_code.html). Motif scanning was performed using the HOMER [17] (Version 4.10) software. Functional annotation of SE-targeted genes was conducted using the ClusterProfiler [18] (Version 4.8.3) and org.Mm.eg.db (Version 3.17.0) packages, with statistical significance set at *p* < 0.05.

### Revealing SE methylation signatures at single-cell resolution

Data preparation and integration: The data processing and analysis pipeline for methylation involves several key steps: (1) demultiplexing of FASTQ files into individual cells; (2) quality control at the reads level; (3) sequence alignment; (4) BAM file processing. The process begins with raw sequencing data in FASTQ format, which is aligned to the reference genome using tools such as Bismark. During this stage, additional preprocessing steps, including filtering, sorting, and duplicate removal, are conducted to enhance data quality and minimize technical artifacts. The aligned reads are then stored in BAM format and subsequently transformed into ALLC files using the ALLCools package [19] (https://github.com/lhqing/ALLCools). The ALLC files, which provide base-level methylation or accessibility information, undergo further extraction and processing. The resulting ALLC data are then converted into count matrix outputs and can be integrated with SE information, enabling a more comprehensive and detailed analysis.

Integration, clustering and SE annotation: First, reads were mapped to the mouse mm10 genome, generating single-cell methylation-specific files. Second, clustering, identification of methylated regions, and integration of additional datasets were performed at the single-cell level. In brief, cells were filtered based on key mapping metrics: (1) overall mCG level > 0.4; (2) overall mCH level < 0.2; (3) total final reads > 500; (4) Bismark mapping rate > 0.1. Genomic bin features (100 kb) were filtered by excluding bins with mean total cytosine base calls below 500 (low coverage) or above 3,000 (unusually high coverage). And then performed principal component analysis (PCA) on the scaled mc level matrix. The number of principal components (PCs) was selected by inspecting the variance ratio of each PC using the elbow method. The CH and CG PCs were then concatenated together for further analysis in clustering and manifold learning.

A consensus clustering [20] approach was employed, based on multiple iterations of Leiden clustering [21] over a k-nearest neighbor (KNN) graph, to account for the stochastic variability of the Leiden algorithm. Using fixed resolution parameters, Leiden clustering was repeated on the KNN graph with different random initializations, and the resulting cluster assignments were aggregated into a new feature matrix, with each individual Leiden result representing a distinct feature. Manifold learning techniques, including t-distributed stochastic neighbor embedding (t-SNE) [22] and Uniform Manifold Approximation and Projection (UMAP) [23] were applied to the principal component matrix, consistent with the input used for clustering. These algorithms were implemented via the Scanpy [24] (Version 1.10.1) package. Additionally, SEs location information was integrated with the identified clusters for further analysis.

### Integration of scBS-seq data reveals age-associated DMRs and methylation changes at SE loci

Comparing old and young groups, differentially methylated regions (DMRs) were identified by integrating scBS-seq data using the ALLCools package, based on pooled methylation data from all cells derived from 3 old and 3 young mice. First, differentially methylated sites (DMSs) were identified using the RMS (Root-Mean-Square) test implemented in Methylpy [25] (Version 1.3). This test compares methylation levels across multiple replicates and computes a root-mean-square statistic to assess differences between groups. Sites with an adjusted *p* < 0.01 were considered significant. DMRs were defined as genomic regions containing at least four significant DMSs, with any pair of adjacent DMSs located within 100 kb. This analysis resulted in the identification of 1,663 DMRs. Following the analysis of methylation data, we assessed the overlap between DMRs and SEs using Intervene [26] (Version 0.6.5).

### SnATAC-seq data processing, dimensionality reduction, and cell type annotation

Fragment files for each library were downloaded, and transcription start site enrichment scores, fragment counts, and doublet scores for each nucleus were calculated using ArchR [27] (Version 1.0.3) in conjunction with BSgenome.Hsapiens.UCSC.hg38 (Version 1.4.5). Nuclei with enrichment scores below 4 or fewer than 1,000 fragments were removed. Doublets were filtered out using the filterDoublets function with a filterRatio of 1.5. Subsequently, the data were further processed using latent semantic indexing-based dimensionality reduction on 500 bp genomic tiles via the addIterativeLSI function. Batch correction across samples was performed using Harmony. ScRNA-seq data were employed to achieve accurate cluster identification using restricted integration techniques. Following this, the UMAP embedding method was utilized to visualize single cells within the reduced-dimensional space.

### Analysis of genes targeted by SEs

A reproducible peak set was established in ArchR using the addReproduciblePeakSet function. Subsequently, differentially enriched peaks were identified with the getMarkerFeatures function, applying thresholds of log2FC > 1 and a false discovery rate (FDR) < 0.05 to ascertain statistically significant peaks. Collaborative accessibility among genomic regions was computed using the addCoAccessibility function. After integrating snATAC-seq data, the addPeak2GeneLinks function was used to examine the co-accessibility between chromatin peaks and their linked genes.

### Analysis of motif enrichment within SEs

The addMotifAnnotations function was utilized to identify motifs enriched in differential peaks, including those found in the Marker peak, by leveraging the CisBP motif database. Through grouping analysis, the enriched motifs in different young and old groups were ranked based on their significance. Z-scores were derived after performing bias correction with chromVAR (Version 0.2.0), and the distribution of Z-scores enriched for TFs was analyzed using the getVerDeviations function. TFs occupancy was analyzed via TF footprinting, utilizing the getFootprints function to calculate putative binding sites while incorporating the Tn5 insertion bias into the analysis.

The addTrajectory function was employed to construct the trajectory of MuSC differentiation into type I fibers. Subsequently, the getTrajectory function was utilized to visualize alterations in the motif matrix and gene expression matrix along the differentiation trajectory, resulting in the generation of a pseudo-time heat map.

### Cell-cell communication analyses

Cell-cell communication analyses were performed on the scRNA-seq data using CellChat [28, 29] (v1.6.0). Specifically, CellChat was run separately for each group, utilizing log-normalized expression data and cell annotation information for the analysis. All analyses were conducted following the standard pipeline (https://github.com/sqjin/CellChat/blob/master/tutorial/CellChat-vignette.html) with default parameters and the human ligand-receptor database.

### Histology and immunofluorescence

Paraffin sections were obtained from muscle tissue, air-dried, and subsequently fixed and washed in PBS. The sections were permeabilized with 0.5% Triton X-100 for 30 min. Blocking was performed using a buffer containing 4% bovine serum albumin (BSA) for 30 min. Following the blocking step, the sections were incubated overnight at 4 °C with primary antibodies targeting plexin-D1 (PLXND1) (1:100, #bs-12739R, Bioss, Beijing, China) and paired box 7 (PAX7) (1:100, #20570-1-AP, Proteintech, Wuhan, China), (1:100, #gb113190-100, Servicebio, Wuhan, China). The next day, the slides were incubated with fluorescein-labeled secondary antibodies for 50 min at room temperature in the dark, followed by nuclear counterstaining with DAPI. Fluorescence images were acquired using an upright fluorescence microscope (Nikon, Tokyo, Japan) and recorded with a Pannoramic MIDI Scanner (3DHISTECH, Budapest, Hungary).

### Statistical analysis

Quantification of fluorescence signal intensities from immunofluorescence images was performed using ImageJ. Data are presented as mean ± standard deviation (SD). Statistical comparisons between two groups were conducted using unpaired two-tailed Student’s *t*-tests, with *p* < 0.05 considered statistically significant. In the analysis of snATAC-seq data, differential chromatin accessibility was determined using the getMarkerFeatures function, applying thresholds of FDR < 0.05 and log2FC > 1 to identify significantly enriched peaks. Motif enrichment and TF activity analyses were conducted using chromVAR, based on bias-corrected Z-scores. TFs with higher absolute Z-scores were interpreted as having greater regulatory activity. Positive regulators were identified according to two stringent criteria: (1) a Pearson correlation coefficient greater than 0.5 between motif activity and gene score with an FDR-adjusted *p* < 0.01, ensuring statistical robustness; and (2) TFs exhibiting the greatest inter-cluster variability, ranking within the top 50% of Z-scores. For DNA methylation analysis, DMSs were detected using the RMS test, with adjusted *p* < 0.01 considered significant. DMRs were defined as genomic regions containing at least 4 DMSs. Functional enrichment analyses, including Gene Ontology and pathway enrichment, were performed using ClusterProfiler, with statistical significance defined as *p* < 0.05. All statistical analyses were carried out using GraphPad Prism (v9.0) or appropriate R/Bioconductor packages.

## Results

### 1124 SEs were identified and analyzed

**Fig. 2:**
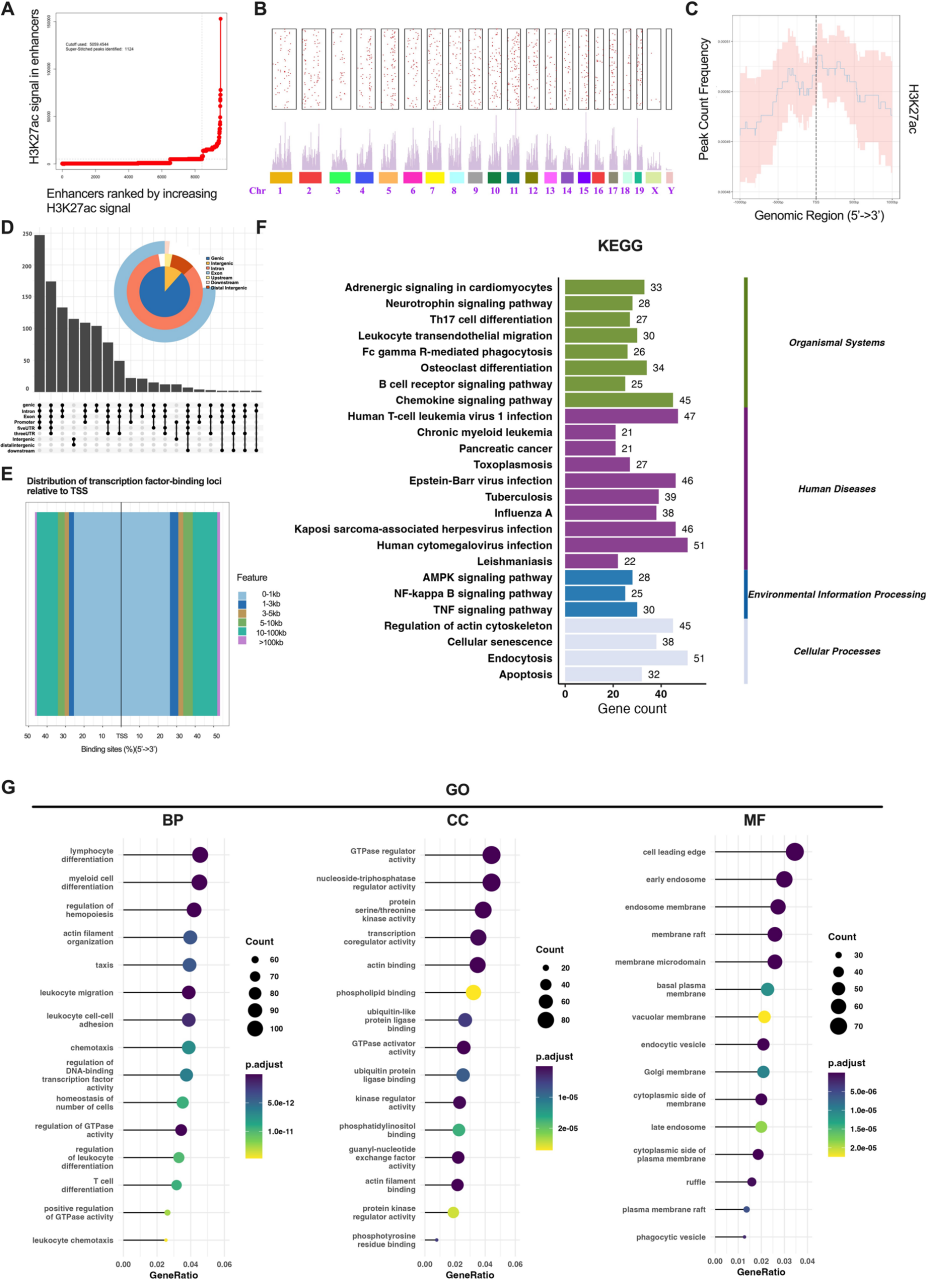
The genomic landscape of SEs in MSCs. **A**: Based on the signal strength of H3K27ac, 1124 super-enhancers were identified using the ROSE algorithm, with a cutoff value of 5,059.45. These enhancers were ranked and named according to their signal intensity. **B**: Genomic distribution of 1,124 SEs across chromosomes. Each dot represents one SE. SEs tend to be enriched in chromosomal regions with higher H3K27ac ChIP-seq signal peaks, as shown in the bar plot below. **C**: The average binding profile of peaks near TSS regions, with a 95% confidence interval, was examined. This profile is shown as a graph indicating the read count frequency from − 1000 bp to + 1000 bp around the TSS. **D**: A matrix layout displays the interactions among the 7 main regions, arranged by size, with dark circles indicating intersecting sets. **E**: Distribution of the distances from each peak to the nearest gene: Determine the closest gene by measuring the distance from the peak to the transcription start site, irrespective of whether the peak is in an intron, intergenic region, or another genomic location. **F**: KEGG analysis showed that these genes are involved in cell differentiation, actin cytoskeleton regulation, apoptosis, and other processes. **G**: GO analysis indicated that these genes are strongly linked to cell differentiation, GTPase regulator activity, and different membrane functions

Using the ROSE algorithm to analyze H3K27ac ChIP-seq data from MSCs, we identified 1,124 SEs (Fig. 2A). These SEs were ranked and annotated based on their H3K27ac signal intensity. The chromosomal distribution of SEs is presented in Fig. 2B, where each point corresponds to a single SE. The accompanying bar plot depicts the aggregate H3K27ac signal peaks, representing enhancer activity across genomic loci. The peaks of H3K27ac are predominantly concentrated around the TSS, spanning from − 1000 bp to + 1000 bp, as illustrated in Fig. 2C. The specific features of SEs, displayed in Fig. 2D, indicate that SEs encompass various regions of DNA, including distal intergenic regions. Additionally, the distribution of genes nearest to SEs is presented in Fig. 2E, with approximately 60% of these genes located within 3000 kb of the TSS. A comprehensive analysis of the 1,124 SEs using gene ontology (GO) and Kyoto Encyclopedia of Genes and Genomes (KEGG) pathways revealed significant enrichment of SE-targeted genes in categories such as cell differentiation, cellular senescence, and transcriptional activity (as depicted in Fig. 2F and G). These findings align with our hypothesis that SEs play a regulatory role in determining cell fate.

### Construction and integrated analysis of SEs methylation profiles at the cellular and genomic levels

**Fig. 3:**
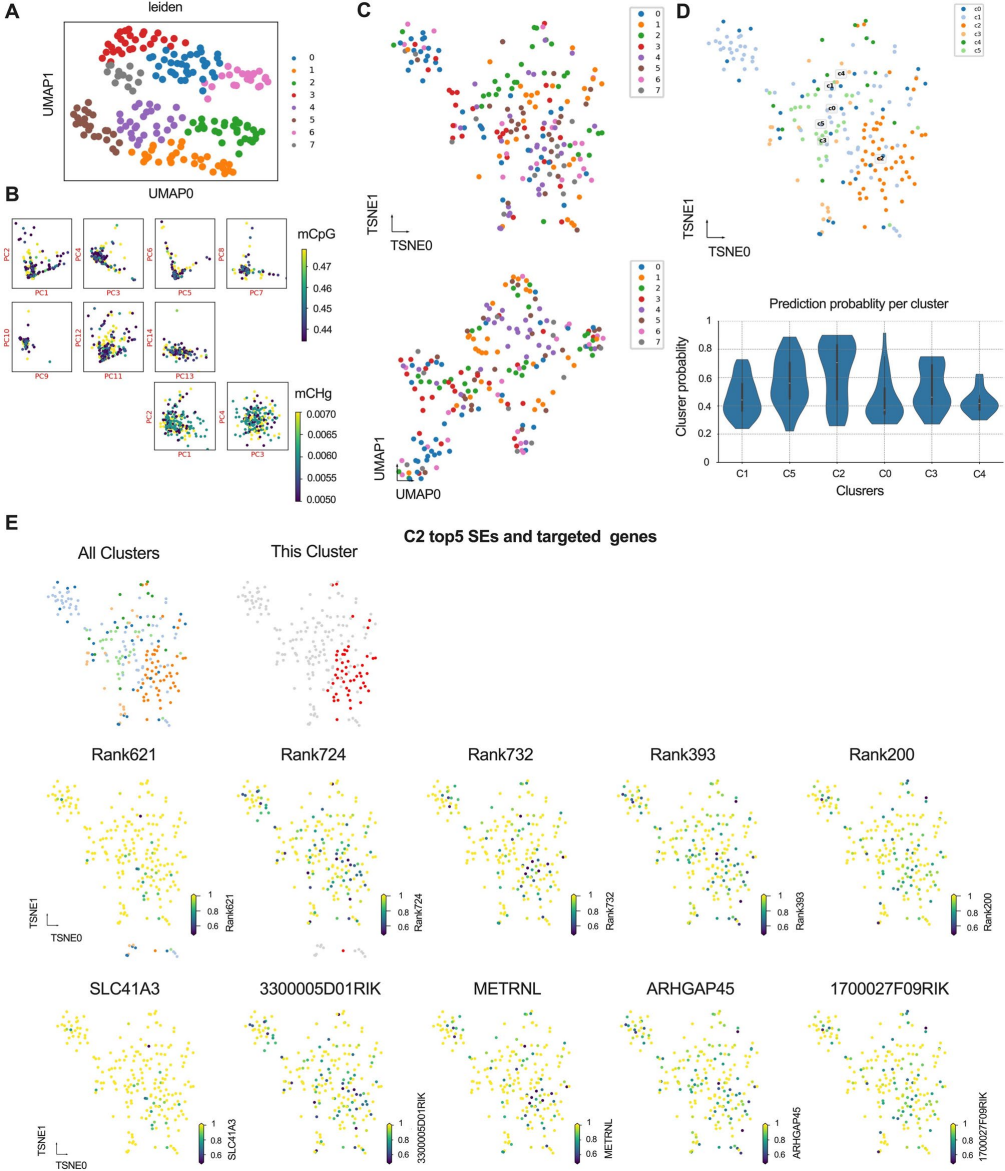
The methylation patterns of SEs were analyzed and characterized in cellular profiles. **A**: The highly variable features (HVFs) are used for pre-clustering by the Leiden algorithm. **B**: PCA was initially performed separately on the mCH and mCG matrices, with the mCG matrix showing a greater variety of principal components compared to the mCH matrix. **C**: Manifold learning was applied to the data using both t-SNE (top) and UMAP (bottom) techniques. **D**: Consensus clustering was performed to identify and define 6 distinct clusters within the group (top), and the final prediction probabilities for these clusters were shown (bottom). **E**: The methylation profiles of the top 5 SEs and genes in cluster 2 were presented, given that cluster 2 was the most prominent of the 6 clusters

To characterize the SE methylation profiles, we initially identified 27,315 highly variable features (HVFs) from the scBS-seq dataset. HVFs refer to genomic regions that display high variability in methylation levels across single cells. A cell-by-feature methylation fraction matrix was constructed using these HVFs, which enabled preliminary clustering of the cells into 8 groups. Feature enrichment scores were then computed for each cluster to highlight cluster-specific methylation signatures (Fig. 3A).

To further capture distinct methylation contexts, we performed PCA separately on the mCG and mCH methylation matrices. The top principal components from each matrix were concatenated to generate an integrated low-dimensional representation of the methylome. This combined feature space was used for subsequent k-nearest neighbor (KNN) clustering (Fig. 3B). Dimensionality reduction techniques, including t-SNE and UMAP, were applied to visualize cellular relationships in the reduced space (Fig. 3C). Given the relatively limited number of single cells and the sensitivity of manifold learning algorithms to data sparsity, we further employed consensus clustering to improve the robustness of cluster assignment. This approach yielded 6 stable and reproducible cell clusters, with final cluster identities and prediction probabilities illustrated in Fig. 3D. SE loci were integrated with the scBS-seq dataset. We then calculated and assessed the methylation levels of each SE across individual cells using the area under the receiver operating characteristic curve (AUROC). SEs were subsequently prioritized based on their AUROC scores. The top five SEs, along with their putative target genes associated with Cluster 2, are shown in Fig. 3E and Supplementary Figs. 1 and 2.

**Fig. 4:**
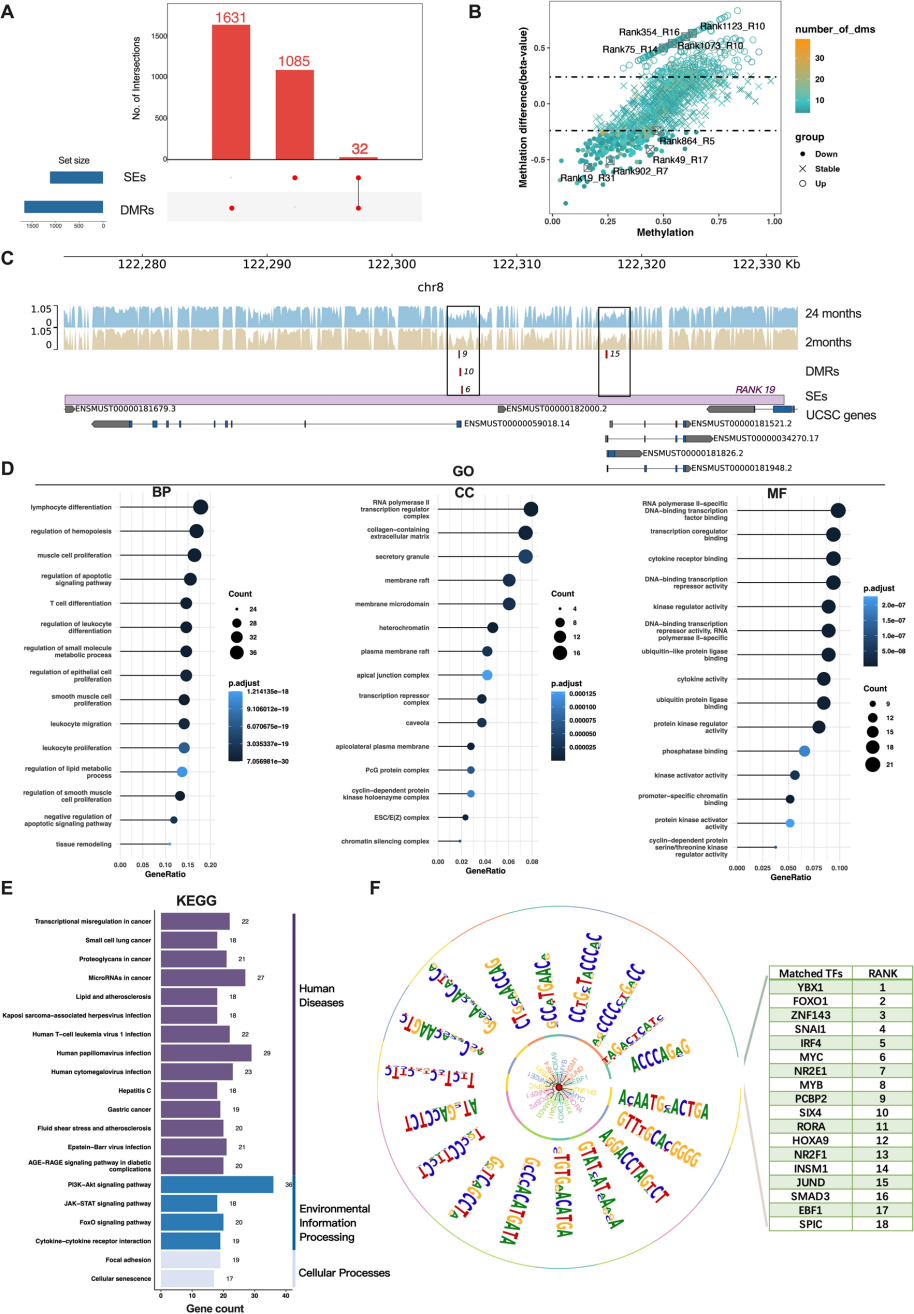
Identification and functional characterization of DMRs within SEs. **A**: 32 SEs were identified from the overlapping analysis between DMRs and SEs. **B**: The DMRs were presented in a volcano plot, highlighting the SEs within the DMRs that had the highest number of DMSs. **C**: SE Rank 19 contains a total of 40 DMPs within 4 DMRs, potentially influencing the transcription of more than 8 nearby genes. **D**: GO analysis showed that these genes are closely linked to cell differentiation, cell proliferation, and transcriptional regulation. **E**: KEGG analysis demonstrated that these genes participate in the PI3K-Akt signaling pathway, FOXO signaling pathway, and cancer-related regulatory processes. **F**: Motif analysis using HOMER on the 32 SEs screened out 18 motifs and associated TFs, with YBX1 being the top-ranked factor

Differentially methylated sites (DMSs) between young (2 months) and old (24 months) mice were identified, with regions containing more than 4 DMSs defined as DMRs. A total of 1,663 DMRs were identified, with 32 overlapping with SEs following the analysis (Fig. 4A). The top 8 DMRs, characterized by the highest number of DMSs within SEs, are presented in Fig. 4B. For example, SE Rank 19 contains a total of 40 DMPs, potentially influencing the transcription of more than eight neighboring genes (Fig. 4C).

Further GO and KEGG analyses of the target genes associated with these 32 SEs revealed strong correlations with muscle cell proliferation, transcription coregulator binding, and the FOXO signaling pathway, underscoring the significant role these SEs play in the aging process (Fig. 4D and E). Motif analysis of the 32 SEs identified 18 motifs along with their corresponding TFs, with YBX1, FOXO1, and ZNF143 showing a particularly strong association with SE-related activities (Fig. 4F).

### Integrative multi-omics and cross-species analysis of age-associated methylated SEs in MuSCs

#### 1) YBX1, FOXO1, and SNAI1 are critical TFs for regulating SE activities

Given the dynamic nature of methylation changes, snATAC-seq and scRNA-seq analyses of human skeletal muscle cells were conducted to further elucidate the regulatory patterns of these differentially methylated SEs on downstream motifs and target genes.

**Fig. 5:**
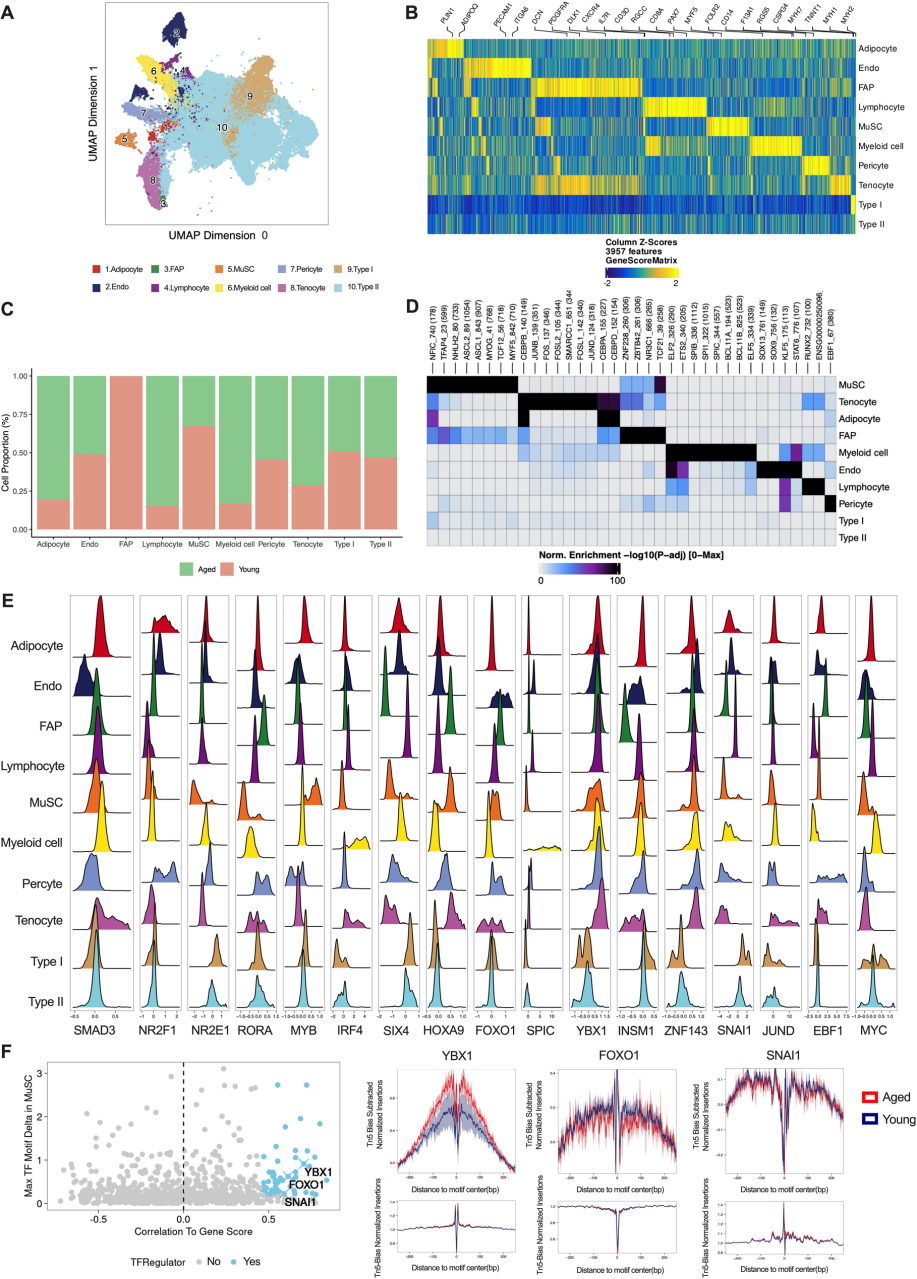
The comprehensive analysis was performed using snATAC-seq on human skeletal muscle cells, comparing samples from aged and young individuals. **A**: UMAP analysis of 258,642 cells revealed 10 distinct skeletal muscle cell populations. **B**: The heatmap illustrated the different cell types identified in the snATAC-seq analysis, with each cell type labeled by its specific marker genes. **C**: The proportions of each cell type were calculated and compared between the aged and young individuals. **D**: Open chromatin peak regions corresponding to the top 9 motifs were highlighted for each cell type. **E**:17 TFs were analyzed using Z-scores across all cell types to evaluate their regulatory roles. **F**: Positive regulatory factors, such as YBX1, FOXO1, and SNAI1, were marked in the volcano plot to highlight their relevance(left). Footprint analysis of YBX1, FOXO1, and SNAI1 was conducted in MuSCs, comparing their binding patterns between the aged and young groups to explore potential age-related differences in their activity(right)

First, snATAC-seq data was integrated with scRNA-seq to robustly identify the main cell types, including adipocytes, endothelial cells (Endo), fibro/adipogenic progenitors (FAP), MuSCs, lymphocytes, myeloid cells, pericytes, tenocytes, type I myofibers, and type II myofibers (Fig. 5A), utilizing specific cell markers and marker peaks as illustrated in Fig. 5B and Supplementary Fig. 3A. Cell proportion analyses demonstrated progressive shifts in the populations of adipocytes, FAPs, lymphocytes, MuSCs, and myeloid cells with aging (Fig. 5C).

Differential features between the aged and young groups for each cell type were calculated and visualized using MA plots (Supplementary Fig. 3B). In MuSCs, 107 features were upregulated, and 555 were downregulated. Further, to identify enriched motifs in peaks that were either up- or down-regulated in each cell type, we extended the differential feature analysis, with partial results shown in Fig. 5D. Based on peak accessibility (FDR ≤ 0.1, log2FC ≥ 0.5), the ranked motifs and associated TFs were compared between the young and aged in MuSC groups. In the aged group, the top three motifs were NFYB, SP2, and PBX3, while in the young group, NR3C1, AR, and PGR were the top motifs. This distinct motif ranking underscores the differences in chromatin accessibility and regulatory features between the two groups (Supplementary Fig. 3C).

To enhance the accuracy of activity predictions for the 18 TFs involved in the methylation reprogramming of SEs, a Z-score analysis was performed to evaluate the activity levels of these TFs. This approach enables a more robust assessment of TF activity by accounting for expression variability across different conditions. Higher absolute Z-scores were associated with greater read depth, thereby improving prediction accuracy. We conducted a detailed analysis of the Z-score distribution across 18 motifs and identified 17 TFs that consistently showed significant activity. Notably, SMAD3 exhibited a consistent Z-score pattern across all cell types, while JUND had the highest Z-score across all cell types. These findings indicate their strong regulatory activity and suggest their roles as key TFs with broad influence in various cellular processes. Furthermore, YBX1 in MuSCs appeared to have a higher Z-score compared to other cell types, suggesting a stronger regulatory activity specifically within MuSCs (Fig. 5E). In the detailed analysis of MuSCs during aging, JUND, SNAI1, INSM1, YBX1, and IRF4 emerged as the top 5 TFs with the highest Z-scores. Their elevated Z-scores underscore their critical roles in the transcriptional networks that regulate age-related changes in MuSC function (Supplementary Fig. 3D).

To gain a deeper understanding of motif dynamics during the aging process, an integrated analysis was conducted to identify key motifs involved in regulatory changes. Positive regulators were defined based on two rigorous criteria: first, TFs needed to exhibit a correlation coefficient greater than 0.5 between motif activity and gene score, with an adjusted *p* of less than 0.01, ensuring the robustness and statistical significance of the association. Second, TFs that demonstrated the largest differences between clusters and ranked within the top 50% of Z-scores were also classified as positive regulators, reflecting their substantial variation in activity across different clusters. This comprehensive analysis led to the identification of positive regulatory factors, including YBX1, FOXO1, and SNAI1, which are likely to play crucial roles in the regulatory networks governing aging-related processes (Fig. 5F). Using foot printing analysis to precisely identify TF binding sites, YBX1 demonstrated a significantly more pronounced binding trough, indicating strong binding activity. This trend was especially prominent in the aged group of MuSCs, suggesting an enhanced regulatory role for YBX1 in the transcriptional networks associated with aging. This approach provides a comprehensive understanding of the functional roles these TFs play within the broader cellular landscape.

#### 2) Identification of key regulatory genes linked to SEs in MuSCs.

**Fig. 6:**
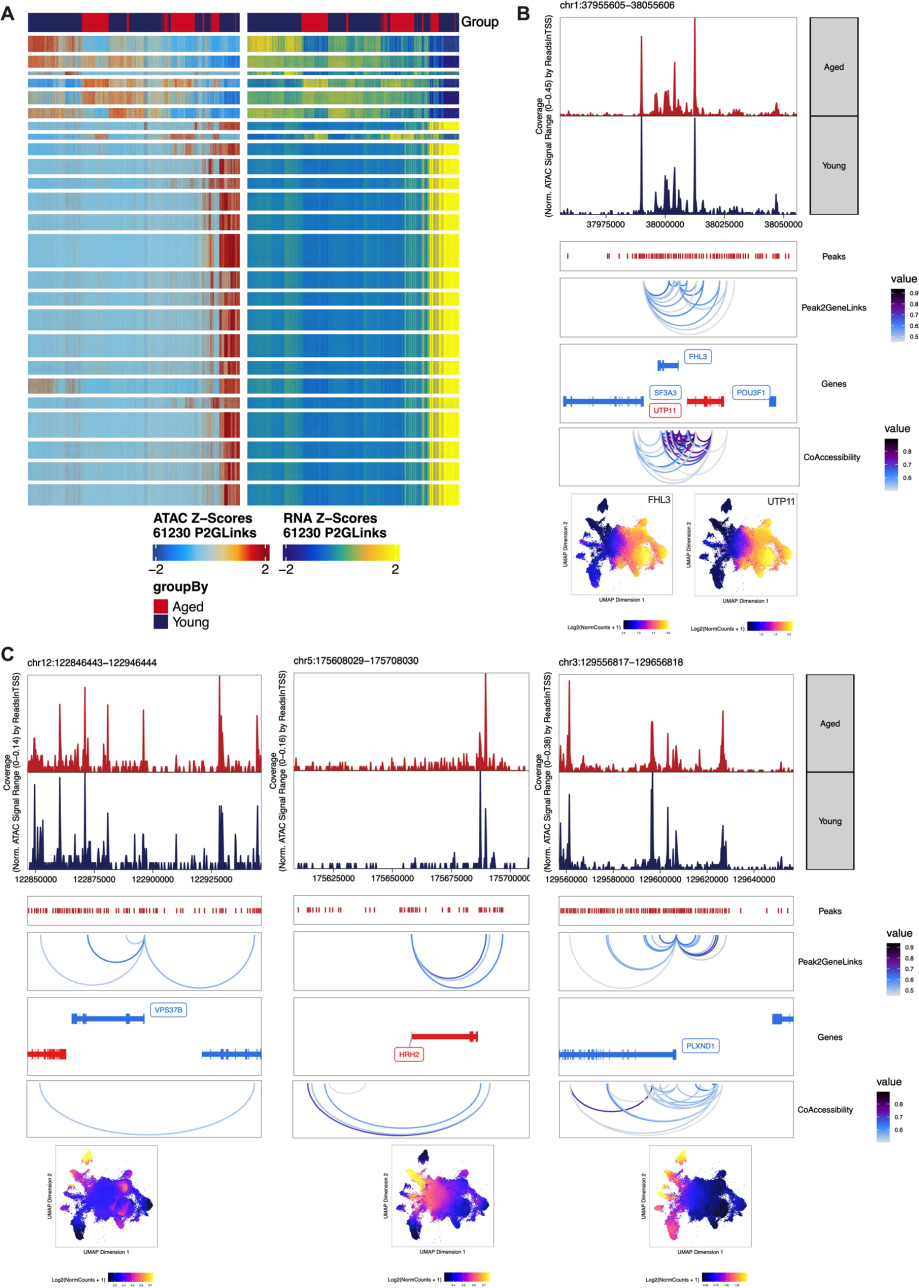
The expression of SE-targeted genes was analyzed for correlation with peak accessibility. **A**: The peak-to-gene heatmap visualized the correspondence of all peak-to-gene links, with snATAC-seq data on the left and scRNA-seq on the right. **B** and **C**: The co-accessibility and peak-to-gene linkages for FHL3, UTP11, VPS37B, HRH3, and PLXND1 were specifically highlighted. The UMAP mapping (bottom) represented the expression of these genes across different cell types

To elucidate the regulatory networks of the genes targeted by the 32 SEs, we integrated scRNA-seq data to assess the correlation between peak accessibility and gene expression. This was visualized using peak-to-gene and co-accessibility linkage analysis. Initially, peak-to-gene heatmaps were generated to display the correspondence of all peak-to-gene links, with snATAC-seq data represented on the left and scRNA-seq data on the right, allowing for a comprehensive view of the relationship between chromatin accessibility and gene expression (Fig. 6A). Subsequently, we identified the top five genes exhibiting the strongest peak-to-gene associations and co-accessibility links: FHL3, UTP11, VPS37B, HRH3, and PLXND1. These genes demonstrated the most significant correlation between chromatin accessibility and gene expression, highlighting their potential regulatory significance within the SE-associated networks (Fig. 6B and C).

Using UMAP embedding to map gene expression scores (bottom of Fig. 6B and C), we observed a marked concentration of PLXND1 expression specifically within MuSCs. This targeted expression pattern highlights the potential significance of PLXND1 in the regulatory landscape of MuSCs, prompting our interest in further investigating its role.

#### 3) PLXND1 showed a decreasing expression trend during aged, linked to the hypermethylation of SE Rank 869 possibly

**Fig. 7:**
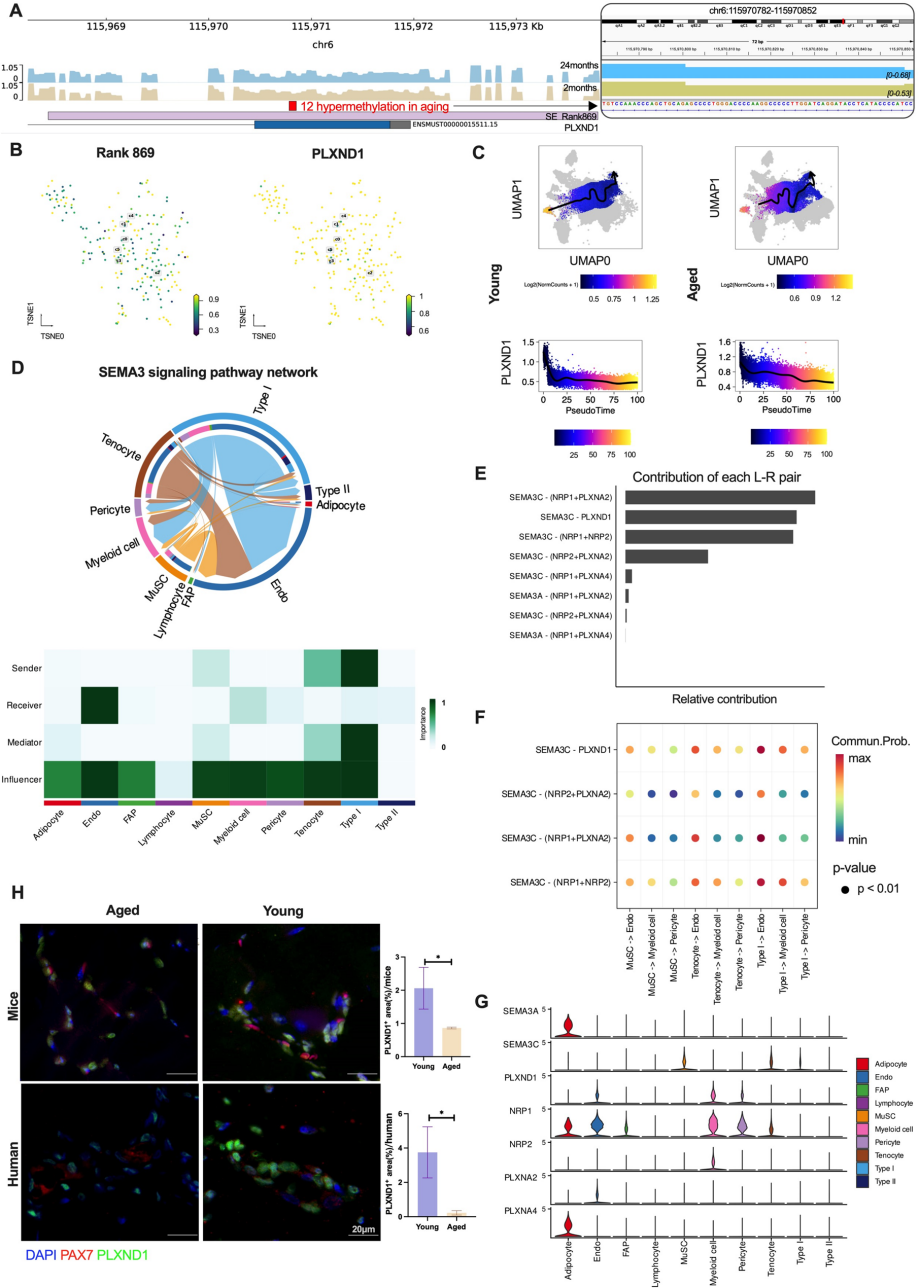
The investigation of the role of PLXND1 in MuSC. **A**: The methylation status of PLXND1 and its associated super-enhancer located on chromosome 6 was analyzed, revealing 12 hypermethylated differentially methylated sites within the region. A magnified view highlights a hypermethylated subregion at chr6:115970782–115,970,852. In the old group, the methylation signal ranged from 0 to 0.68, whereas in the young group, it ranged from 0 to 0.53. **B**: the methylation profiles of SE ranked 869 and PLXND1 exhibited distinct differences. **C**: The expression of PLXND1 progressively decreased over pseudo-time in both the young (left) and aged (right) groups. However, the young group exhibited a higher initial expression of PLXND1 compared to the aged group at the early stages. **D**: According to the CellChat analysis, the SEMA3 pathway was significantly activated in the communication between MuSCs and other cell types (top), exerting a pronounced influence on a broad spectrum of cell types (bottom). **E** & **F**: PLXND1 and SEMA3C were key ligand-receptor pairs in the SEMA3 pathway involved in cell-cell communication, particularly in the interactions between MuSCs and Endo, myeloid cells, and pericytes. **G**: The expression of SEMA3C was notably highest in MuSCs, while PLXND1 expression was significantly elevated in Endo, myeloid cells, and pericytes, suggesting a key role for the SEMA3C-PLXND1 axis in modulating cell communication across these cell populations. **H**: The representative images (left) and corresponding quantification (right) of immunofluorescence analysis illustrate the PLXND1 + area, with PLXND1 marked in green, the muscle marker PAX7 in red, and nuclei in blue, comparing aged and young cohorts in both human and murine samples. The scale bar represents 20 μm, with a sample size of *n* = 3 individuals for each group

Based on the methylation atlas, we further examined the methylation status of the PLXND1-associated super-enhancer, SE Rank 869 (Fig. 7A). This super-enhancer harbors a DMR containing 12 differentially methylated sites, with increased methylation observed in the aged group. Further analysis revealed a distinct difference in methylation patterns between SE Rank 869 and PLXND1 (Fig. 7B). We hypothesized that the observed hypermethylation at SE Rank 869 may be associated with the expression of PLXND1. To investigate this, we explored the dynamic expression of PLXND1 throughout MuSC differentiation into muscle cells (Type I) using pseudotime analysis in both the young and aged groups. While PLXND1 expression progressively decreased during differentiation in both groups, its levels were significantly lower in MuSCs from the aged group compared to the young group (Fig. 7C). This suggests that age-related reductions in PLXND1 expression may contribute to the diminished regenerative capacity of MuSCs in older individuals, highlighting the potential link between the hypermethylation of SE Rank 869 and the age-associated decline in PLXND1 expression.

Cell to cell communication analysis was conducted to explore the role of PLXND1 in MuSCs (Supplementary Fig. 4). As a result, the Class 3 semaphorin (SEMA3) signaling pathway was prominently activated in the communication between MuSCs, Endos, myeloid cells, and pericytes. Endo acted as the primary recipients, while MuSCs, tenocytes, and type I served as the main senders and mediators. A broad spectrum of cell types acted as influencers in this signaling network (Fig. 7D). The SEMA3C-PLXND1 axis played a central role in the SEMA3 signaling pathway, serving as a major contributor to the regulation of intercellular communication during this signaling network (Fig. 7E). Also, the SEMA3C-PLXND1 axis was identified as the most significant ligand-receptor(L-R) pair in orchestrating intercellular communication between MuSCs, Endos, myeloid cells, and pericytes (Fig. 7F). As depicted in Fig. 7G, SEMA3C expression was notably highest in MuSCs, while PLXND1 expression was significantly upregulated in Endos, myeloid cells, and pericytes.

Given the high conservation of PLXND1 between mice and humans, its distribution was corroborated in both human and mouse muscle tissues. Immunofluorescence analysis was performed to visualize PLXND1 expression, with PAX7 (a marker for skeletal muscle stem cells) labeled in red and PLXND1 labeled in green. The results revealed a significant reduction in the area positive for PLXND1 in the aged group compared to the young group in both human and murine muscle tissues. This reduction in PLXND1 expression in aging muscle suggests a potential link to impaired muscle regeneration and function (Fig. 7H). Dysregulation of SEMA3 signaling has been implicated in various conditions, including cancer metastasis, neurodegenerative diseases, and vascular pathologies, making it a promising target for therapeutic interventions aimed at modulating these signaling pathways [30]. Our findings highlight the critical role of the SEMA3C-PLXND1 axis within the SEMA3 signaling pathway, with particular relevance to muscle regeneration in MuSCs during aging.

## Discussion

In this study, we described the methylation profile of SEs in MuSCs for the first time using H3K27ac ChIP-seq and scBS-seq, providing new insights into the regulatory roles of SEs. Furthermore, the methylation dynamics of the SEs during aging were systematically investigated, resulting in the identification of 32 key SEs. The associated downstream motifs and target genes were comprehensively analyzed. To further explore these findings, we conducted bioinformatic analyses on publicly available snATAC-seq and scRNA-seq datasets, providing robust evidence for the regulatory interactions between these SEs and their downstream motifs and genes. This dual approach strengthens the correlation between methylated SEs and gene expression patterns, offering deeper insights into the molecular mechanisms governing muscle stem cell function and regeneration. Moreover, pseudotime analysis and immunofluorescence staining confirmed that PLXND1 expression was downregulated in aged MuSCs, which was associated with the hypermethylation of SE Rank 869 during aging. These findings suggest that SE-mediated methylation changes contribute to the age-related decline in PLXND1 expression, potentially impairing MuSC function and muscle regeneration.

The role of SEs in regulating cell fate was first proposed in 2015. SEs drive the expression of key genes that control cell identity and function, and in cancer, they can activate genes that promote tumor growth and spread, making them important research targets [31]. Beyond cancer, SEs have potential in combating aging by maintaining tissue health and regulating stem cell function. By controlling genes involved in cell renewal, differentiation, and stress response, SEs may help preserve tissue regenerative capacity and delay age-related decline. However, the role of SEs in aging is not well understood, and more research is needed to explore their potential in promoting healthy aging and preventing age-related diseases.

Aging is also associated with changes in the epigenetic landscape, such as DNA methylation and chromatin remodeling. Studies have shown a strong link between DNA methylation and aging; factors like disease, aging, and unhealthy lifestyles can change DNA methylation patterns at specific CpG sites and across the methylome. Mayne et al. noted that DMPs are used to create “epigenetic clocks” that accurately predict biological age [32]. This project therefore investigated the methylation reprogramming of SEs in MuSCs during aging. Through the SE methylation reprogramming atlas, we can easily obtain the methylation status of specific SEs or genes in MuSCs with high precision, providing clear and reliable insights into epigenetic regulation at both the single-cell and whole-genome levels.

We initially selected MSC-derived SEs due to their well-characterized profiles and established roles in stem cell regulation, which provided a valuable reference framework for analyzing methylation dynamics. Although this study focuses on aging-associated epigenetic changes in MuSCs, there is currently a lack of SE datasets derived specifically from MuSCs under aged conditions. Given the developmental and functional similarities between MSCs and MuSCs, including shared transcription factors and signaling pathways such as FOXO and Wnt, MSC-derived SEs were considered a biologically relevant alternative [33–35]. Furthermore, previous studies have shown that some SEs are conserved or functionally important across related stem cell types [1, 15] supporting the rationale for their use in this analysis.

To ensure biological specificity, all downstream analyses including motif enrichment, integration with snATAC-seq and scRNA-seq data, as well as transcription factor activity evaluation were performed using MuSC-specific datasets. This approach ensured that the identification of key regulators such as YBX1, FOXO1, and SNAI1 accurately reflects epigenetic regulation in MuSCs during aging.

Through KEGG and GO analysis of the downstream motifs and genes associated with the 32 methylated SEs, we identified these SEs as crucial regulatory molecules. More than two significant skeletal muscle multi-omics sequencing studies have been published in 2024, providing comprehensive datasets that have deepened our understanding of muscle aging at the single-cell level [9, 10]. We leveraged these publicly available datasets to corroborate our findings and perform integrative analyses. While these landmark studies focused primarily on the cellular and transcriptomic landscape of aging skeletal muscle, our investigation uniquely concentrated on SE-associated regulatory mechanisms in MuSCs, particularly the identification of open chromatin regions and downstream TF motifs enriched within SE domains.

Integrative analysis revealed YBX1 as a key TF motif associated with age-related SEs in MuSCs, suggesting its role in transcriptional remodeling during aging. It was reported that YBX1 deficiency leads to gene mis-splicing, contributing to BMSC aging and differentiation shifts, with its deletion accelerating bone loss and its overexpression enhancing bone formation in mice [36]. And YBX1 could interact with lncRNA DSCAM-AS1 to disrupt the role of SE in inactivating the FOXA1 transcription network in cancer [37]. These studies highlight YBX1 as a pivotal regulatory molecule within the SE regulatory network, strongly supporting our research predictions and underscoring its significance in gene regulation.

In our analysis, alongside FOXO1 and SNAI1, YBX1 was identified as one of the key TFs enriched at SE regions with age-dependent methylation changes. Notably, footprinting analysis revealed deeper YBX1 binding patterns in aged MuSCs, indicating greater transcriptional engagement with SEs. These findings suggest that YBX1 is not merely a marker of aging but also a functional mediator of age-related transcriptional drift. Its consistent association with super-enhancer dynamics and regulatory remodeling during aging underscores its potential involvement in the decline of MuSC regenerative capacity. Given its established functions in other stem cell systems, we propose that YBX1 operates as a conserved regulator of SE**-** mediated gene expression throughout the aging process. Future studies aimed at comprehensive mapping of YBX1 binding sites, identification of its downstream target genes, and elucidation of its mechanistic role in SE regulation are warranted. Such investigations hold significant promise for advancing our understanding of stem cell aging and may ultimately contribute to the development of innovative therapeutic approaches to mitigate muscle degeneration.

SnATAC-seq was employed to assess the chromatin accessibility of the SE regions and to examine the correlation between their openness and the expression of downstream target genes. This approach enabled the identification of genes regulated by SEs, including FHL3, UTP11, VPS37B, HRH3, and PLXND1. Upon visualizing PLXND1 expression, we observed its predominant expression in MuSCs, prompting us to select it for further analysis. In our study, pseudotime analysis revealed a decreasing trend in PLXND1 expression in aged MuSCs, which might be associated with hypermethylation of SE Rank 869. This finding was further supported by immunofluorescence staining of muscle tissues from both humans and mice, where a reduced expression of PLXND1 was observed in the aged groups. PLXND1 is highly conserved across multiple species and is recognized as a receptor that plays a crucial role in signaling pathways regulating cell migration, axon guidance, and vascular development [38, 39]. Recent reports have highlighted that the importance of PLXND1 in regulating vascular function by serving as a mechanosensor in endothelial cells [40]. Additionally, endothelial cells are integral to muscle regeneration, as they provide lactate to macrophages, which effectively stimulates the formation of new muscle fibers [41].

Our CellChat analysis identified SEMA3C-PLXND1 as a key mediator in the SEMA3 signaling pathway, facilitating communication between MuSCs, Endos, myeloid cells, and pericytes. The SEMA3 signaling pathway negatively regulates endothelial cell migration and angiogenesis, thereby maintaining the integrity of the vascular network. By modulating endothelial cell behavior, SEMA3 ensures proper vascular organization and prevents aberrant angiogenic sprouting, preserving both the structural and functional integrity of the vasculature [42]. Additionally, the SEMA3 pathway has been implicated in regulating T cell responses, promoting synoviocyte proliferation, and inducing pro-inflammatory cytokine production by monocytes [43, 44]. Overall, disruption of the SEMA3 signaling pathway compromises both muscle repair mechanisms and the immune environment, thereby contributing to the reduced regenerative capacity typically observed in aging muscle [45]. These findings suggest that PLXND1 plays a critical role in regulating muscle aging and may be significantly involved in the pathogenesis of muscle loss and decline in strength.

Furthermore, we found a striking difference in the methylation status between PLXND1 and SE Rank 869, as shown in Fig. 7A. This significant divergence suggests a complex regulatory interplay that could be critical for understanding the mechanisms of PLXND1 expression and its role in muscle aging and regeneration. We also examined the methylation status of an additional 31 SEs and assessed the corresponding methylation changes in their target genes. Interestingly, although the methylation status of the majority of SEs was consistent with that of their target genes, notable exceptions were observed for FCNB, GRAMD4, and EEA1 (Supplementary Fig. 5). The differential methylation between SEs and their downstream genes offers compelling evidence of a sophisticated epigenetic regulatory network mediated by SEs. This intricate mechanism not only highlights the role of SEs in modulating gene expression but also underscores their potential to orchestrate complex cellular functions and influence cell fate decisions, particularly in the context of aging and disease.

This study has several limitations that warrant consideration. First, due to the lack of MuSC-specific SE annotations, we used 1,124 MSC-derived SEs as a proxy to study age-related methylation, which may cause some regulatory discrepancies. Future efforts to define SEs directly in young and aged MuSCs will help to refine these findings and enhance cell-type specificity. Second, although we employed multiple strategies to minimize batch effects, including stringent quality control, normalization across datasets, and principal component analysis for batch effect correction, integrating multi-omics data from different platforms, sources, and collection time points remains a significant challenge. Considering the temporal and environmental sensitivity of DNA methylation [46, 47] we cannot completely rule out the possibility that residual batch effects or experimental heterogeneity contributed to the observed patterns, particularly the limited overlap among some datasets. Finally, although SE Rank 869 was identified as a candidate regulatory region associated with PLXND1 expression in aged MuSCs, the causal nature of this interaction remains to be determined. Functional assays such as CRISPR interference, enhancer deletion, or chromatin conformation capture would be necessary to elucidate the molecular mechanism by which this SE modulates PLXND1 and its role in skeletal muscle stem cell aging [48, 49].

## Conclusion

Our study offers a valuable framework for exploring SE–associated methylation reprogramming in aging stem cells and lays the foundation for future mechanistic and therapeutic investigations into MuSCs regulation during aging.

## Supplementary Information

Below is the link to the electronic supplementary material.

Supplementary Material 1

## Data Availability

Data is provided within the manuscript or supplementary information files.

## Abbreviations

SE: Super-enhancer
H3K27ac: Histone H3 Lysine 27 acetylation
GATA3: GATA Binding Protein 3
AP-1: Activating protein-1
FOXA1: Forkhead box A1
ATRA: All-trans retinoic acid
snATAC-seq: Single-nucleus ATAC sequencing
ENCODE: ENCyclopedia of DNA elements
ChIP-seq: Chromatin immunoprecipitation sequencing
scRNA-seq: Single-cell RNA sequencing
MSC: Mesenchymal stem cells
GEO: GENE expression omnibus
CNGB: China national GeneBank
scBS-seq: Single-cell bisulfite sequencing
MSCs: Mesenchymal stem cells
TSS: Transcription start sites
DMR: Differentially methylated region
KNN: k-nearest neighbor
FDR: False discovery rate
PLXND1: Plexin-D1
PAX7: Paired box 7
BSA: Bovine serum albumin
GO: Gene ontology
KEGG: Kyoto encyclopedia of genes and genomes
HVF: Highly variable features
AUROC: The receiver-operating characteristic curve
DMS: Differentially methylated sites
YBX1: Y-Box binding protein 1
FOXO1: Forkhead box protein
FOXO: Forkhead box O
ZNF143: Zinc finger protein 143
Endo: Adipocytes, endothelial cells
FAP: Fibro/adipogenic progenitors
MuSCs: Skeletal muscle stem cells
NFYB: Nuclear transcription factor Y subunit beta
SP2: Sp2 transcription factor
PBX3: PBX homeobox 3
NR3C1: Nuclear receptor subfamily 3 group C member 1
AR: Androgen receptor
SNAI1: Snail family transcriptional repressor 1
PGR: Progesterone receptor
SMAD3: SMAD family member 3
JUND: JunD proto-oncogene, AP-1 transcription factor subunit
INSM1: INSM transcriptional repressor 1
IRF4: Interferon regulatory factor 4
FHL3: Four and A half LIM domains 3
UTP11: UTP11 small subunit processome component
VPS37B: VPS37B subunit Of ESCRT-I
HRH3: Histamine receptor H3
FCNB: Ficolin B
GRAMD4: GRAM domain containing 4
EEA1: Early endosome antigen 1
SEMA3: Class 3 semaphorin
L-R: Ligand-receptor
