# Supplementary material for: Multi-omic integration of single-cell data uncovers methylation profiles of super-enhancers in skeletal muscle stem cells
Source: Epigenetics Chromatin. 2025 Aug 11;18:54. doi: 10.1186/s13072-025-00619-0 (PMC12337566; doi:10.1186/s13072-025-00619-0)
Supplement: Supplementary file 1 — Supplementary Material 1 [file 13072_2025_619_MOESM1_ESM.zip › Supplementary data/supplefig1.pdf]

Cluster c0 Top SEs

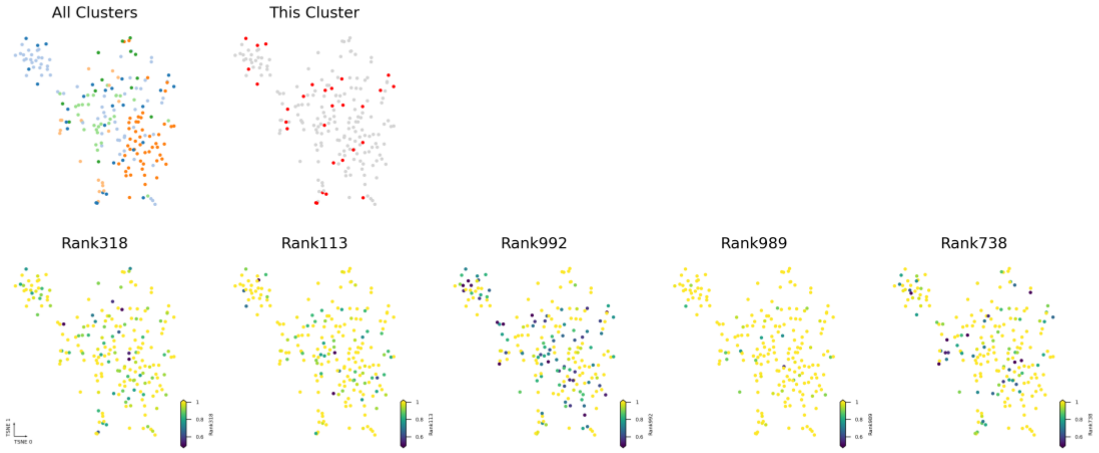

Cluster c1 Top SEs

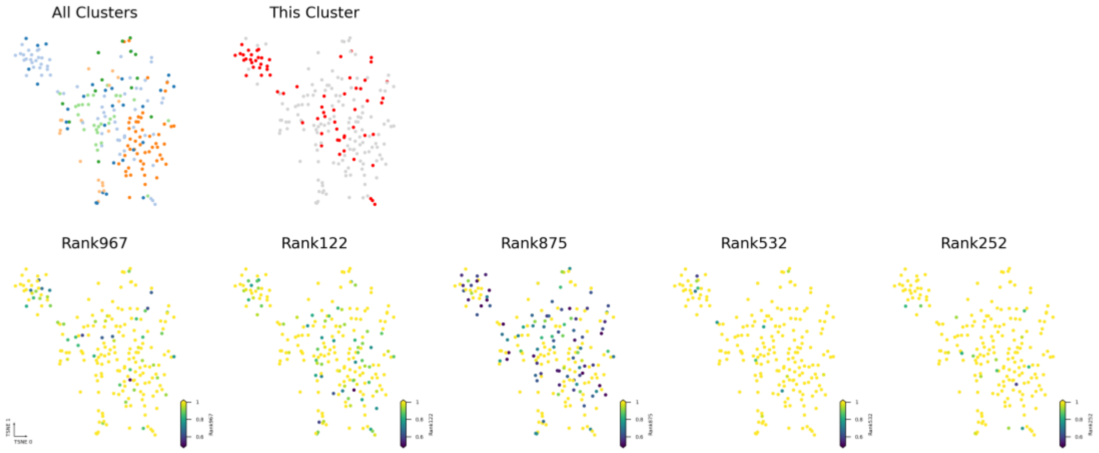

Cluster c2 Top SEs

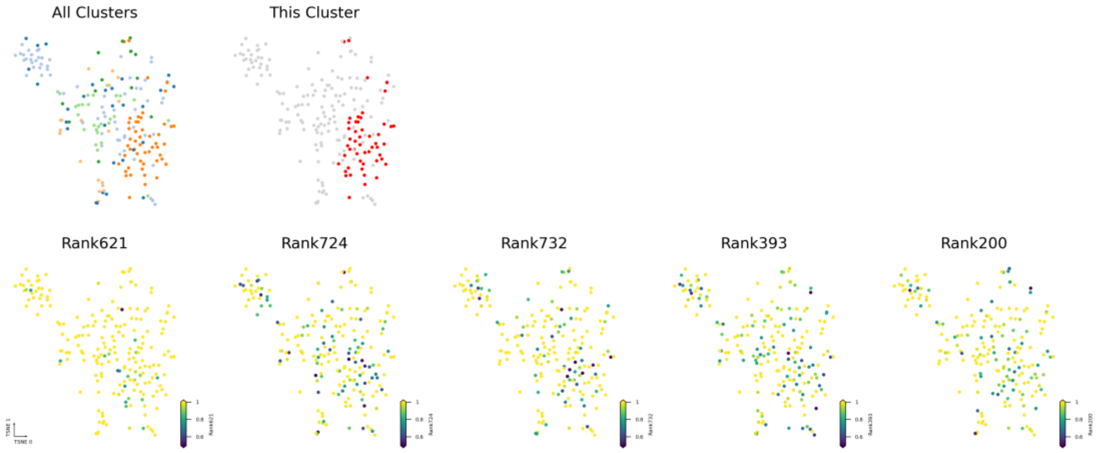

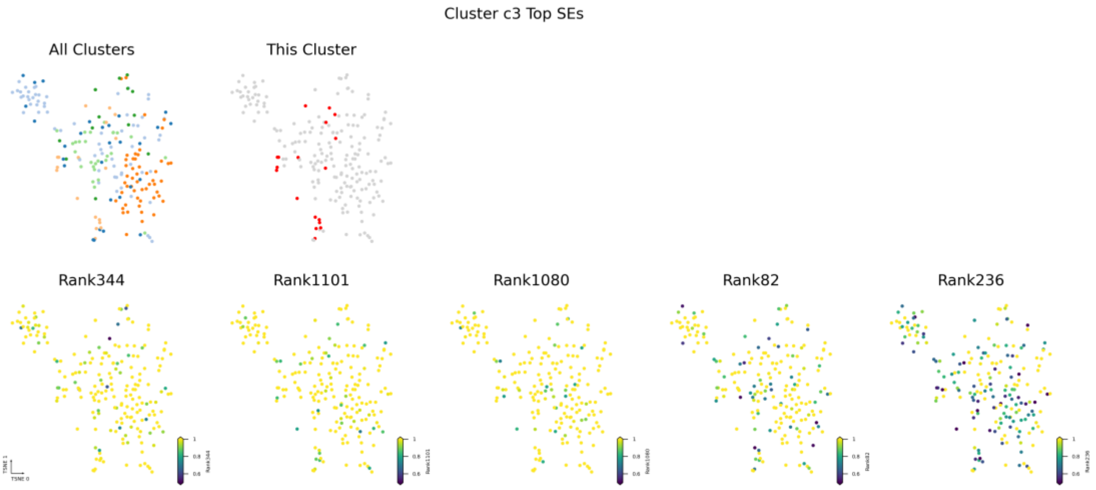
Cluster c3 Top SEs
All Clusters
This Cluster
Rank344
Rank1101
Rank1080
Rank82
Rank236

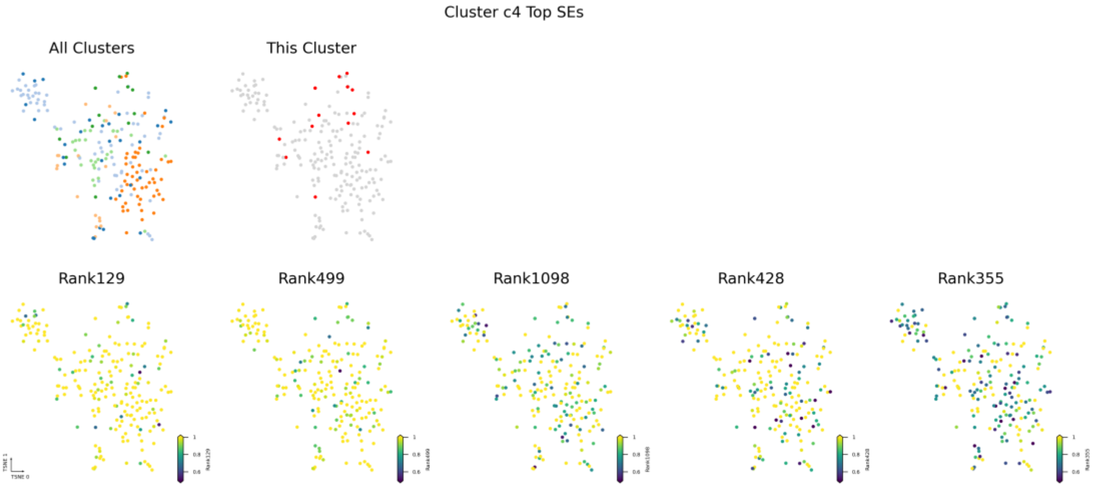
Cluster c4 Top SEs
All Clusters
This Cluster
Rank129
Rank499
Rank1098
Rank428
Rank355

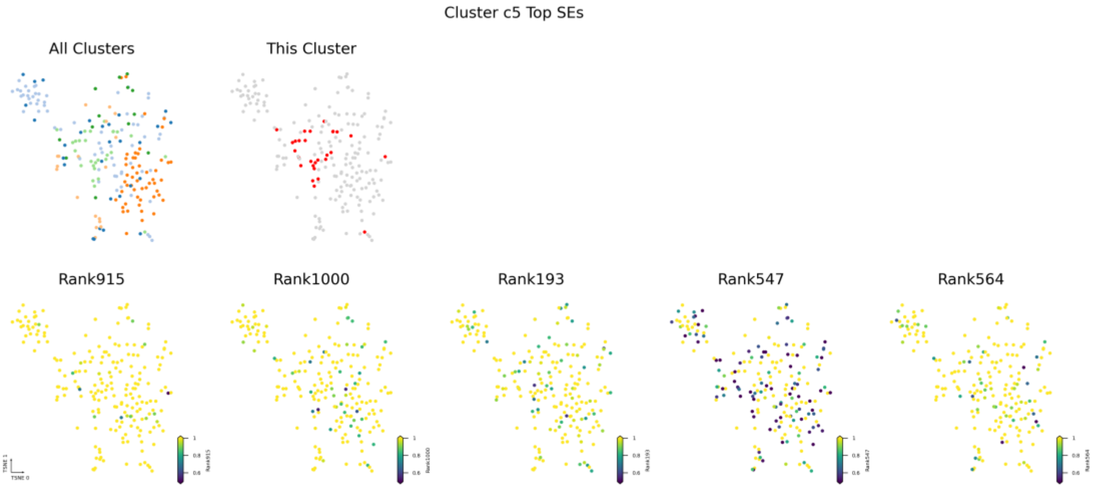
Cluster c5 Top SEs
All Clusters
This Cluster
Rank915
Rank1000
Rank193
Rank547
Rank564
