# Supplementary material for: Multi-omic integration of single-cell data uncovers methylation profiles of super-enhancers in skeletal muscle stem cells
Source: Epigenetics Chromatin. 2025 Aug 11;18:54. doi: 10.1186/s13072-025-00619-0 (PMC12337566; doi:10.1186/s13072-025-00619-0)
Supplement: Supplementary file 1 — Supplementary Material 1 [file 13072_2025_619_MOESM1_ESM.zip › Supplementary data/supplefig2.pdf]

Cluster c0 Top SEs

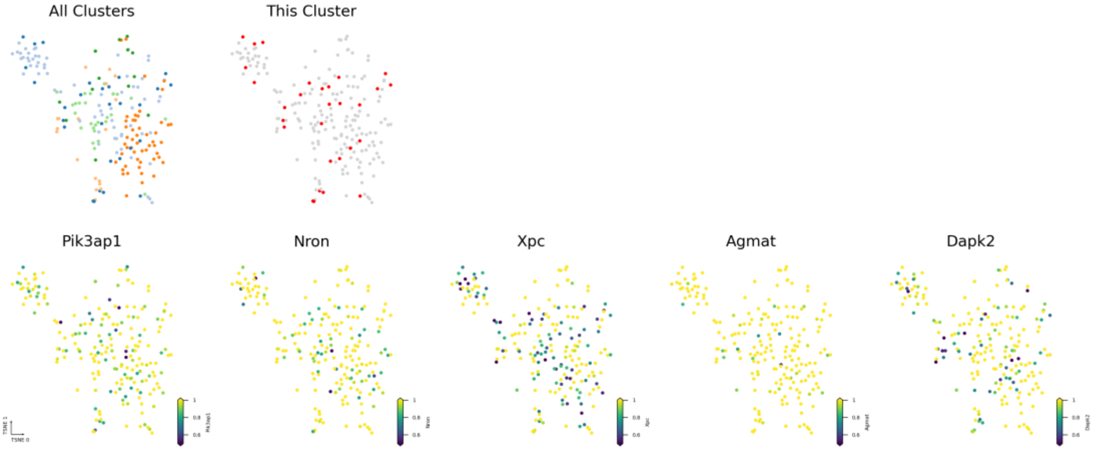

Cluster c1 Top SEs

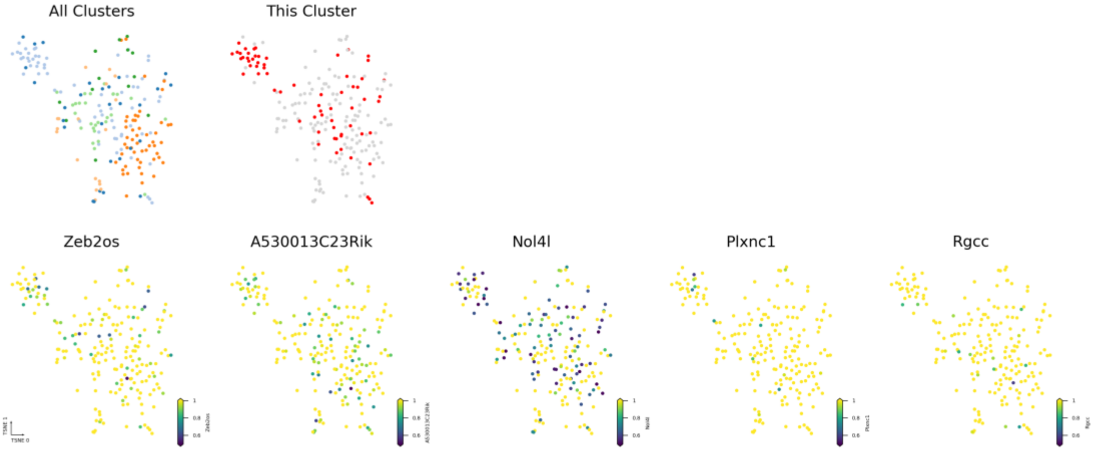

Cluster c2 Top SEs

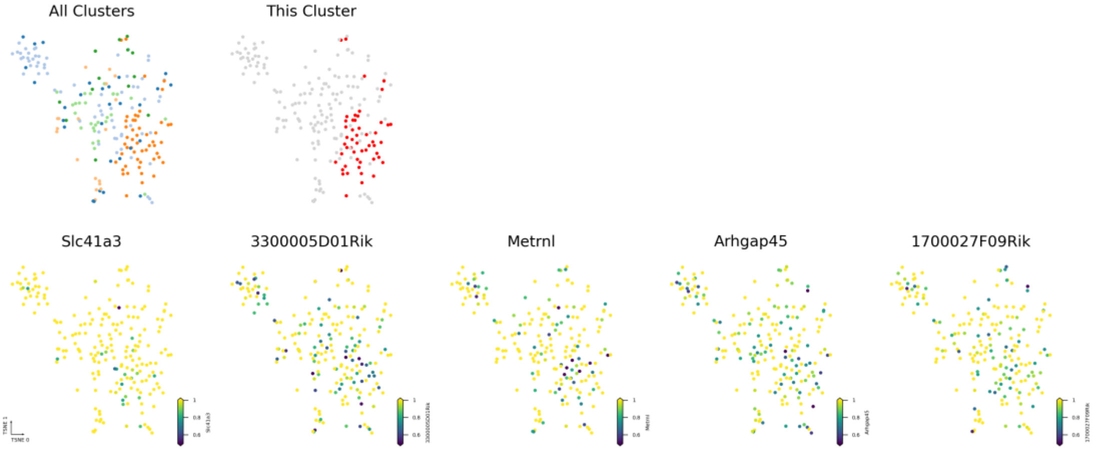

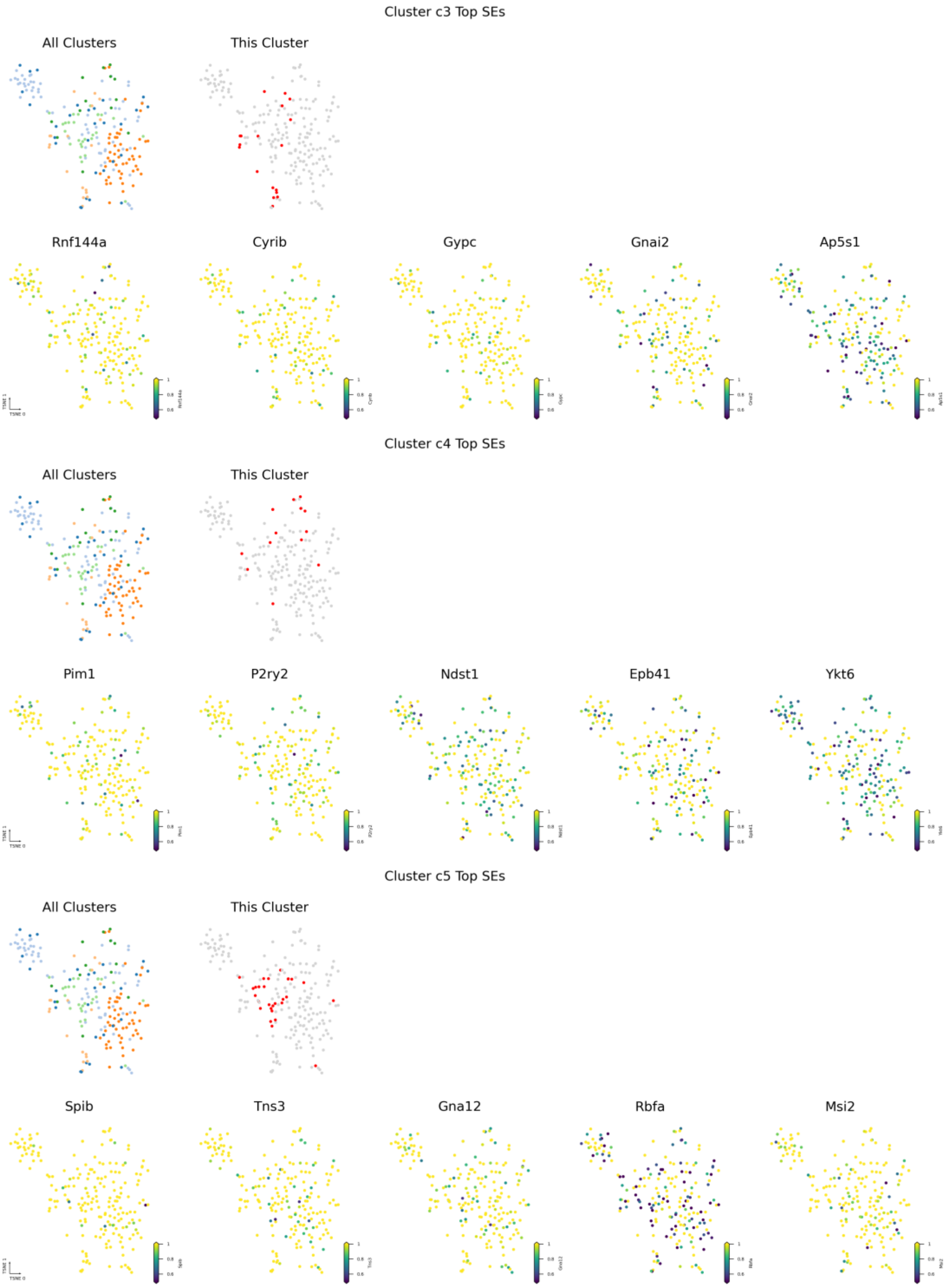
Cluster c3 Top SEs
All Clusters
This Cluster
Rnf144a
Cyrib
Gypc
Gnai2
Ap5s1
Cluster c4 Top SEs
All Clusters
This Cluster
Pim1
P2ry2
Ndst1
Epb41
Ykt6
Cluster c5 Top SEs
All Clusters
This Cluster
Spib
Tns3
Gna12
Rbfa
Msi2
