# Supplementary material for: Multi-omic integration of single-cell data uncovers methylation profiles of super-enhancers in skeletal muscle stem cells
Source: Epigenetics Chromatin. 2025 Aug 11;18:54. doi: 10.1186/s13072-025-00619-0 (PMC12337566; doi:10.1186/s13072-025-00619-0)
Supplement: Supplementary file 1 — Supplementary Material 1 [file 13072_2025_619_MOESM1_ESM.zip › Supplementary data/Supplementary Table 1.pdf]

Supplementary Table 1. Information of 1124 super enhancers

REFSEQ	SE(rank)	REGION_ID	chrom	start	end	gene_id
NM_001001182	156	11__lociStitched	chr2	60070859	60089121	Baz2b
NM_001001183	544	5__lociStitched	chr17	25086681	25091170	Tmem204
NM_001001738	381	8__lociStitched	chr19	47904596	47915908	Itprip
NM_001001806	1	38__lociStitched	chr17	84089458	84185524	Zfp36l2
NM_001001932	270	9__lociStitched	chr10	95765274	95781298	Eea1
NM_001001932	1124	10__lociStitched	chr10	96150591	96201801	Eea1
NM_001002786	908	7__lociStitched	chr17	29162581	29172695	Rab44
NM_001003918	235	13__lociStitched	chr16	8745801	8779208	Usp7
NM_001004164	676	5__lociStitched	chr10	88412510	88422982	Gnptab
NM_001004180	608	5__lociStitched	chr5	114590493	114597812	Fam222a
NM_001005419	295	9__lociStitched	chr10	67695640	67717054	Ado
NM_001005421	694	5__lociStitched	chr9	45084207	45095801	Jaml
NM_001008542	1092	10__lociStitched	chr19	53303537	53326772	Mxi1
NM_001008706	574	5__lociStitched	chr13	3861175	3867111	Calm5
NM_001009545	622	5__lociStitched	chr12	113422287	113430469	Adam6b
NM_001010941	128	12__lociStitched	chr5	146675626	146704304	Gpr12
NM_001013365	273	9__lociStitched	chr11	4223662	4240674	Osm
NM_001013373	597	5__lociStitched	chr9	45293998	45301045	Tmprss13
NM_001013792	514	5__lociStitched	chr15	27594952	27598438	Otulin
NM_001015046	573	5__lociStitched	chr11	74571321	74577216	Rap1gap2
NM_001018042	179	11__lociStitched	chr2	73037197	73064593	Sp3
NM_001018042	383	8__lociStitched	chr2	72995651	73007546	Sp3
NM_001024474	422	8__lociStitched	chr13	52533841	52552071	Diras2
NM_001024955	396	8__lociStitched	chr13	101539217	101553587	Pik3r1
NM_001025254	933	7__lociStitched	chr18	82565599	82578703	Mbp
NM_001025255	51	17__lociStitched	chr18	82504226	82543732	Mbp
NM_001025379	796	5__lociStitched	chr14	31211944	31229166	Sema3g
NM_001029842	1031	7__lociStitched	chr11	109470280	109496624	Slc16a6
NM_001033219	127	12__lociStitched	chr15	73618528	73646284	Slc45a4
NM_001033222	1053	7__lociStitched	chr19	59350117	59385905	Pdzd8
NM_001033266	455	8__lociStitched	chr11	89077625	89101292	Gm525
NM_001033308	365	16__lociStitched	chr4	132778236	132817512	Themis2
NM_001033339	521	5__lociStitched	chr17	23632745	23636599	Mmp25
NM_001033393	95	14__lociStitched	chr11	115190127	115242894	Tmem104
NM_001033399	330	9__lociStitched	chr13	43239601	43269171	Gfod1
NM_001033430	500	5__lociStitched	chr6	39257695	39259980	Kdm7a
NM_001033463	70	21__lociStitched	chr6	113644448	113693207	Tatdn2
NM_001033465	274	9__lociStitched	chr7	139970379	139987672	Spef1l
NM_001033539	1122	10__lociStitched	chr16	32160037	32202572	Bex6
NM_001033632	71	21__lociStitched	chr7	140979601	141048376	Ifitm6
NM_001034882	562	5__lociStitched	chr14	79440604	79446181	Kbtbd6
NM_001037298	328	9__lociStitched	chr8	122536966	122565694	Piezo1

NM_001037493	621	5_ _lociStitched	chr6	90622358	90630418	Slc41a3
NM_001037724	858	5_ _lociStitched	chr8	88284701	88309984	Adcy7
NM_001037744	169	11_ _lociStitched	chr14	122044054	122067152	Timm8a2
NM_001039153	801	5_ _lociStitched	chr5	122257896	122275270	Tctn1
NM_001039472	598	5_ _lociStitched	chr1	136133675	136140751	Kif21b
NM_001039723	572	5_ _lociStitched	chr5	123089985	123095871	Tmem120b
NM_001039959	717	5_ _lociStitched	chr19	8980641	8993688	Ahnak
NM_001040111	954	7_ _lociStitched	chr7	101354069	101369304	Arap1
NM_001040398	469	8_ _lociStitched	chr5	123125807	123153300	Setd1b
NM_001042501	99	15_ _lociStitched	chr5	3490785	3516809	Fam133b
NM_001042660	331	9_ _lociStitched	chr18	75363597	75393385	Smad7
NM_001043317	904	7_ _lociStitched	chr7	30886603	30896102	Cd22
NM_001045514	363	16_ _lociStitched	chr4	63373204	63410721	Akna
NM_001045523	582	5_ _lociStitched	chr2	118883131	118889324	Bahd1
NM_001045529	94	14_ _lociStitched	chr16	93767746	93819869	Morc3
NM_001048207	1080	10_ _lociStitched	chr18	32538157	32558614	Gypc
NM_001079686	506	5_ _lociStitched	chr10	5205655	5208647	Syne1
NM_001080548	1109	10_ _lociStitched	chr2	6255281	6286301	Usp6nl
NM_001080935	1066	10_ _lociStitched	chr11	97815270	97830859	B230217C12Rik
NM_001080944	549	5_ _lociStitched	chr2	126495676	126500400	Atp8b4
NM_001080944	914	7_ _lociStitched	chr2	126465801	126476332	Atp8b4
NM_001081092	522	5_ _lociStitched	chr2	179933737	179937600	Taf4
NM_001081104	342	9_ _lociStitched	chr5	65900656	65937647	Chrna9
NM_001081105	910	7_ _lociStitched	chr5	65850727	65861048	Rhoh
NM_001081117	1064	10_ _lociStitched	chr7	135806094	135818598	Mki67
NM_001081151	640	5_ _lociStitched	chr8	117197383	117206426	Gan
NM_001081175	1059	7_ _lociStitched	chr1	180279569	180319404	Itpkb
NM_001081236	1005	7_ _lociStitched	chr5	118360205	118381582	Spring1
NM_001081265	595	5_ _lociStitched	chr5	139160688	139167598	Dnaaf5
NM_001081274	31	18_ _lociStitched	chr4	149146353	149182878	Pgd
NM_001081279	534	5_ _lociStitched	chr8	35564052	35568281	Mfhas1
NM_001081308	1099	10_ _lociStitched	chr5	117135519	117160967	Taok3
NM_001081315	457	8_ _lociStitched	chr17	28808598	28832817	Brpf3
NM_001081337	856	5_ _lociStitched	chr8	125518215	125543314	Sipa1l2
NM_001081355	286	9_ _lociStitched	chr4	143099703	143119549	Prdm2
NM_001081379	841	5_ _lociStitched	chr8	123016329	123037942	Ankrd11
NM_001081408	989	7_ _lociStitched	chr4	141750713	141770057	Agmat
NM_001081433	326	9_ _lociStitched	chr1	54896488	54924108	Ankrd44
NM_001081454	938	7_ _lociStitched	chr7	80444148	80457504	Furin
NM_001081557	316	9_ _lociStitched	chr4	151717464	151742964	Camta1
NM_001081636	72	21_ _lociStitched	chr17	47507277	47579598	Ccnd3
NM_001081650	485	8_ _lociStitched	chr4	62654355	62686858	Rgs3
NM_001081652	957	7_ _lociStitched	chr11	6575940	6591506	Nacad

NM_001081977	344	9__lociStitched	chr12	26379959	26417924	Rnf144a
NM_001083587	1000	7__lociStitched	chr11	8688685	8709276	Tns3
NM_001085355	146	12__lociStitched	chr17	5179055	5220918	Arid1b
NM_001085355	965	7__lociStitched	chr17	5082996	5099572	Arid1b
NM_001085410	871	7__lociStitched	chr15	9074166	9079939	Nadk2
NM_001085491	625	5__lociStitched	chr4	129496453	129504664	Fam229a
NM_001098203	714	5__lociStitched	chr11	75152551	75165509	Hic1
NM_001099217	889	7__lociStitched	chr15	75133969	75142257	Ly6c2
NM_001099276	951	7__lociStitched	chr1	133072774	133087750	Pik3c2b
NM_001099277	405	8__lociStitched	chr7	16043032	16058567	Zfp541
NM_001099314	737	5__lociStitched	chr4	43598822	43612991	Msmp
NM_001099332	812	5__lociStitched	chr3	100993825	101012664	Cd101
NM_001101597	531	5__lociStitched	chr9	31251932	31256101	Gm7244
NM_001101606	615	5__lociStitched	chrX	167103781	167111467	Gm8817
NM_001102471	529	5__lociStitched	chr19	46846163	46850312	Cnnm2
NM_001109661	49	17__lociStitched	chr4	32219489	32258130	Bach2
NM_001109906	687	5__lociStitched	chr2	166966893	166978079	Stau1
NM_001110131	260	9__lociStitched	chr7	25267177	25280555	Cic
NM_001110267	375	8__lociStitched	chr17	46005171	46014447	Vegfa
NM_001110826	294	9__lociStitched	chr9	44561507	44582590	Ddx6
NM_001111073	878	7__lociStitched	chr7	31031233	31038127	Fxyd5
NM_001111121	388	8__lociStitched	chr10	70120397	70133226	Ccdc6
NM_001111311	1020	7__lociStitched	chr1	91041308	91065297	Lrrfip1
NM_001111324	495	8__lociStitched	chr13	41467729	41506226	Nedd9
NM_001112700	18	23__lociStitched	chr11	118183784	118275158	Cyth1
NM_001113350	1024	7__lociStitched	chr8	18911937	18936881	Xkr5
NM_001113353	610	5__lociStitched	chr17	5929397	5936904	Synj2
NM_001113488	135	12__lociStitched	chr11	117316905	117350408	Septin9
NM_001113564	585	5__lociStitched	chr6	67181245	67187675	Serbp1
NM_001114332	559	5__lociStitched	chr10	40128176	40133496	Slc16a10
NM_001114332	540	5__lociStitched	chr10	40146712	40151063	Slc16a10
NM_001122768	997	7__lociStitched	chr5	105641803	105662051	Lrrc8d
NM_001127363	749	5__lociStitched	chr7	139397820	139412548	Inpp5a
NM_001134300	875	7__lociStitched	chr2	153490242	153497009	Nol4l
NM_001134383	1097	10__lociStitched	chr6	90740342	90765642	Iqsec1
NM_001134383	251	9__lociStitched	chr6	90703444	90711091	Iqsec1
NM_001135657	478	8__lociStitched	chr2	90475018	90505295	Ptprj
NM_001135727	718	5__lociStitched	chrX	159637935	159651052	Sh3kbp1
NM_001136065	667	5__lociStitched	chr6	38823122	38833331	Hipk2
NM_001136259	859	5__lociStitched	chr8	75042709	75067993	Tom1
NM_001136484	759	5__lociStitched	chr19	17322012	17337441	Gcnt1
NM_001142323	925	7__lociStitched	chr8	71285777	71298106	Myo9b
NM_001142337	199	11__lociStitched	chr2	103944646	103979078	Lmo2

NM_001142924	776	5_ _lociStitched	chr19	55925794	55942055	Tcf7l2
NM_001145955	799	5_ _lociStitched	chr4	133649836	133667155	Pigv
NM_001146002	820	5_ _lociStitched	chr17	65655772	65675464	Txndc2
NM_001146022	587	5_ _lociStitched	chr14	33189370	33195843	Wdfy4
NM_001146100	188	11_ _lociStitched	chr10	62313967	62344283	Hk1
NM_001146123	206	11_ _lociStitched	chr10	60252011	60290818	Psap
NM_001146176	454	8_ _lociStitched	chr12	76936054	76959689	Max
NM_001146176	913	7_ _lociStitched	chr12	76908698	76919158	Max
NM_001146180	356	16_ _lociStitched	chr15	59037976	59064514	Mtss1
NM_001146318	289	9_ _lociStitched	chr11	100578522	100598846	Cnp
NM_001159290	12	19_ _lociStitched	chr11	24099751	24149677	Bcl11a
NM_001159572	4	25_ _lociStitched	chr10	60329116	60391988	Vsir
NM_001159683	59	27_ _lociStitched	chr2	170147662	170223201	Zfp217
NM_001159904	593	5_ _lociStitched	chr6	128796173	128803020	Klrb1c
NM_001159941	1049	7_ _lociStitched	chr5	114347297	114377968	Kctd10
NM_001160406	77	14_ _lociStitched	chr2	28596580	28620126	Gfi1b
NM_001161365	1037	7_ _lociStitched	chr12	102288328	102315547	Rin3
NM_001161365	61	20_ _lociStitched	chr12	102333410	102369851	Rin3
NM_001162366	163	11_ _lociStitched	chr14	66223471	66244732	Ptk2b
NM_001162905	168	11_ _lociStitched	chr10	118082551	118105218	Mdm1
NM_001162917	1070	10_ _lociStitched	chr9	64789592	64807350	Dennd4a
NM_001162998	864	5_ _lociStitched	chr11	115899429	115929754	Smim6
NM_001163175	515	5_ _lociStitched	chr12	109071484	109075042	Begain
NM_001163262	39	18_ _lociStitched	chr8	117241323	117304521	Cmip
NM_001163288	192	11_ _lociStitched	chr4	59331001	59362703	Susd1
NM_001163288	433	8_ _lociStitched	chr4	59439715	59459916	Susd1
NM_001163336	810	5_ _lociStitched	chr11	72946046	72964734	Atp2a3
NM_001163502	48	17_ _lociStitched	chr12	84172493	84209512	Mideas
NM_001163512	865	7_ _lociStitched	chr5	34974165	34977747	Rgs12
NM_001163816	964	7_ _lociStitched	chr17	57274840	57291265	Vav1
NM_001164040	420	8_ _lociStitched	chr15	74950238	74968346	Ly6e
NM_001164053	742	5_ _lociStitched	chr2	163660810	163675325	Pkig
NM_001164252	838	5_ _lociStitched	chr9	67020046	67041449	Tpm1
NM_001164482	1033	7_ _lociStitched	chr19	5648495	5675165	Sipa1
NM_001164572	707	5_ _lociStitched	chr9	122125971	122138734	Snrk
NM_001164598	239	13_ _lociStitched	chr8	126696399	126736755	Irf2bp2
NM_001164598	54	17_ _lociStitched	chr8	126555066	126598669	Irf2bp2
NM_001164717	1081	10_ _lociStitched	chr19	47431387	47451895	Sh3pxd2a
NM_001165951	505	5_ _lociStitched	chr3	102164404	102167162	Vangl1
NM_001166177	905	7_ _lociStitched	chr11	109548392	109558164	Arsg
NM_001166251	30	18_ _lociStitched	chr6	88726192	88761382	Mgll
NM_001166388	1038	7_ _lociStitched	chr15	73717314	73744699	Ptp4a3
NM_001166432	390	8_ _lociStitched	chr6	117878927	117892068	Hnrnpf

NM_001167680	114	12_ _lociStitched	chr11	116605765	116621746	Rhbdf2
NM_001167746	571	5_ _lociStitched	chr11	118144542	118150399	Dnah17
NM_001167949	524	5_ _lociStitched	chr1	133797644	133801588	Atp2b4
NM_001168277	842	5_ _lociStitched	chr6	52899813	52921442	Jazf1
NM_001168277	704	5_ _lociStitched	chr6	52954503	52967035	Jazf1
NM_001168304	994	7_ _lociStitched	chr10	40442801	40462661	Cdk19
NM_001168526	60	20_ _lociStitched	chr19	32237423	32273181	Sgms1
NM_001168538	511	5_ _lociStitched	chr14	59555028	59558383	Cdadc1
NM_001168693	646	5_ _lociStitched	chr15	97741811	97751068	Endou
NM_001170847	629	5_ _lociStitched	chr19	53773163	53781427	Rbm20
NM_001170960	642	5_ _lociStitched	chr18	49770897	49779978	Dtwd2
NM_001171026	753	5_ _lociStitched	chr10	127126988	127142048	Os9
NM_001172099	824	5_ _lociStitched	chr11	88145045	88164992	Cuedc1
NM_001174107	159	11_ _lociStitched	chr12	81746075	81765656	Map3k9
NM_001177303	231	13_ _lociStitched	chr11	98933392	98963885	Rara
NM_001177650	183	11_ _lociStitched	chr2	31060339	31088738	Fnbp1
NM_001177758	142	12_ _lociStitched	chr2	11512715	11550211	Pfkfb3
NM_001177776	155	11_ _lociStitched	chr10	21076751	21093649	Ahi1
NM_001177833	947	7_ _lociStitched	chr2	131495878	131510297	Smox
NM_001177844	980	7_ _lociStitched	chr2	4555273	4573828	Frmd4a
NM_001177980	703	5_ _lociStitched	chr4	102506182	102518539	Pde4b
NM_001190373	734	5_ _lociStitched	chr18	80408839	80422952	Kcng2
NM_001190886	526	5_ _lociStitched	chr11	34008203	34012159	Kcnip1
NM_001195096	584	5_ _lociStitched	chr10	13303262	13309591	Phactr2
NM_001195529	371	8_ _lociStitched	chr7	99592449	99598283	Tpbgl
NM_001197321	632	5_ _lociStitched	chr6	99204179	99212560	Foxp1
NM_001198984	751	5_ _lociStitched	chr18	60830412	60845230	Tcof1
NM_001199186	278	9_ _lociStitched	chr9	21315554	21333174	Slc44a2
NM_001199676	693	5_ _lociStitched	chr5	122759867	122771381	Camkk2
NM_001205053	1055	7_ _lociStitched	chr12	85624129	85661377	Jdp2
NM_001205339	1014	7_ _lociStitched	chr19	15815605	15838711	Psat1
NM_001205353	75	14_ _lociStitched	chr15	86050380	86071444	Gramd4
NM_001205353	304	9_ _lociStitched	chr15	86087931	86110975	Gramd4
NM_001205355	426	8_ _lociStitched	chr3	84455896	84474552	Fhdc1
NM_001206368	634	5_ _lociStitched	chr2	35232252	35240708	Gsn
NM_001242424	1023	7_ _lociStitched	chr15	27663679	27688458	Otulinl
NM_001243761	451	8_ _lociStitched	chr16	10495815	10518767	Ciita
NM_001243769	376	8_ _lociStitched	chr4	137364861	137374407	Cdc42
NM_001252132	1056	7_ _lociStitched	chr14	74900607	74939559	Lrch1
NM_001252202	209	11_ _lociStitched	chr1	39893564	39936680	Map4k4
NM_001252394	385	8_ _lociStitched	chr11	115631160	115643615	Slc25a19
NM_001252479	65	20_ _lociStitched	chr9	114740290	114794843	Cmtm7
NM_001252506	442	8_ _lociStitched	chr16	23265058	23287166	St6gal1

NM_001252616	613	5_ _lociStitched	chr12	77530455	77538062	Fut8
NM_001252651	823	5_ _lociStitched	chr4	126016075	126035997	Csf3r
NM_001253716	770	5_ _lociStitched	chr14	64420043	64435968	Msra
NM_001253894	470	8_ _lociStitched	chr9	21435364	21463495	Dnm2
NM_001254953	830	5_ _lociStitched	chr17	43537430	43557981	Ankrd66
NM_001256311	9	19_ _lociStitched	chr7	63895168	63938967	E030018B13Rik
NM_001267711	79	14_ _lociStitched	chr7	120860416	120884760	Eef2k
NM_001270537	1084	10_ _lociStitched	chr15	100664294	100685249	Bin2
NM_001271360	769	5_ _lociStitched	chr11	70432122	70448028	Arrb2
NM_001271461	456	8_ _lociStitched	chr2	93447033	93471196	Cd82
NM_001271591	412	8_ _lociStitched	chr5	115058478	115075609	Rpl37rt
NM_001271599	702	5_ _lociStitched	chr3	108286590	108298863	Sort1
NM_001271607	900	7_ _lociStitched	chr1	90240076	90249344	Ackr3
NM_001271843	293	9_ _lociStitched	chr9	71146201	71167184	Aqp9
NM_001276383	716	5_ _lociStitched	chr11	72457825	72470854	Spns2
NM_001277281	325	9_ _lociStitched	chr8	23028021	23055585	Ank1
NM_007377	483	8_ _lociStitched	chr11	120034063	120066099	Aatk
NM_007393	198	11_ _lociStitched	chr5	142902559	142936682	Actb
NM_007395	765	5_ _lociStitched	chr15	101144641	101160239	Acvr1b
NM_007415	806	5_ _lociStitched	chr1	180587220	180605213	Parp1
NM_007420	360	16_ _lociStitched	chr18	62156774	62192381	Adrb2
NM_007421	419	8_ _lociStitched	chr12	112615735	112633787	Adss1
NM_007426	639	5_ _lociStitched	chr8	18760122	18769137	Angpt2
NM_007472	50	17_ _lociStitched	chr6	55320378	55359672	Aqp1
NM_007512	805	5_ _lociStitched	chr4	132513430	132531237	Atp5if1
NM_007520	835	5_ _lociStitched	chr16	87705848	87726807	Bach1
NM_007551	143	12_ _lociStitched	chr9	44509766	44549006	Cxcr5
NM_007564	743	5_ _lociStitched	chr12	80113179	80127698	Zfp36l1
NM_007564	795	5_ _lociStitched	chr12	79827036	79844107	Zfp36l1
NM_007569	605	5_ _lociStitched	chr10	96370516	96377749	Btg1
NM_007570	62	20_ _lociStitched	chr1	134060068	134106549	Btg2
NM_007657	185	11_ _lociStitched	chr6	125461095	125490611	Cd9
NM_007713	404	8_ _lociStitched	chr9	57758229	57773629	Clk3
NM_007717	745	5_ _lociStitched	chr13	24501154	24515748	Cmah
NM_007717	467	8_ _lociStitched	chr13	24393227	24420550	Cmah
NM_007783	306	9_ _lociStitched	chr9	57630169	57653461	Csk
NM_007791	679	5_ _lociStitched	chr1	135681018	135691637	Csrp1
NM_007798	600	5_ _lociStitched	chr14	63124725	63131851	Ctsb
NM_007806	685	5_ _lociStitched	chr8	122427764	122438736	Cyba
NM_007901	473	8_ _lociStitched	chr3	115682291	115711601	S1pr1
NM_007961	792	5_ _lociStitched	chr6	134185521	134202399	Etv6
NM_007961	290	9_ _lociStitched	chr6	134110479	134130898	Etv6
NM_007981	73	21_ _lociStitched	chr8	46474811	46547405	Acsl1

NM_008026	374	8__lociStitched	chr9	32608655	32617334	Fli1
NM_008026	58	27__lociStitched	chr9	32495459	32531652	Fli1
NM_008037	492	8__lociStitched	chr5	32103856	32139921	Fosl2
NM_008052	798	5__lociStitched	chr5	120690110	120707411	Dtx1
NM_008102	131	12__lociStitched	chr14	47086613	47116884	Gch1
NM_008138	82	14__lociStitched	chr9	107611601	107644694	Gnai2
NM_008154	148	12__lociStitched	chr4	133158830	133201399	Gpr3
NM_008155	1094	10__lociStitched	chr7	34210038	34234087	Gpi1
NM_008163	1027	7__lociStitched	chr11	115692738	115717926	Grb2
NM_008210	386	8__lociStitched	chr1	180817848	180830437	H3f3a
NM_008230	379	8__lociStitched	chr2	126596971	126608267	Hdc
NM_008245	343	9__lociStitched	chr19	37466162	37503939	Hhex
NM_008246	764	5__lociStitched	chr3	116634245	116649814	Mfsd14a
NM_008247	579	5__lociStitched	chr13	112746161	112752186	Plpp1
NM_008277	378	8__lociStitched	chr5	123191298	123202249	Hpd
NM_008280	723	5__lociStitched	chr9	70914571	70928028	Lipc
NM_008280	399	8__lociStitched	chr9	70833345	70848117	Lipc
NM_008280	1116	10__lociStitched	chr9	70982767	71019450	Lipc
NM_008286	157	11__lociStitched	chr13	54205172	54223844	Hrh2
NM_008306	1098	10__lociStitched	chr18	60722640	60748080	Ndst1
NM_008320	28	34__lociStitched	chr8	120684839	120798561	Irf8
NM_008338	284	9__lociStitched	chr16	91520257	91539058	Ifngr2
NM_008339	120	12__lociStitched	chr11	106304957	106325944	Cd79b
NM_008348	1069	10__lociStitched	chr9	45235396	45253132	Il10ra
NM_008349	444	8__lociStitched	chr16	91394805	91417039	Il10rb
NM_008359	162	11__lociStitched	chr6	120465376	120486428	Il17ra
NM_008391	779	5__lociStitched	chr8	46788546	46804959	Irf2
NM_008391	972	7__lociStitched	chr8	46841712	46859598	Irf2
NM_008404	232	13__lociStitched	chr10	77520300	77551797	Itgb2
NM_008452	1086	10__lociStitched	chr8	72344132	72365627	Klf2
NM_008489	398	8__lociStitched	chr2	158309260	158323779	Lbp
NM_008507	755	5__lociStitched	chr5	121824355	121839578	Sh2b3
NM_008511	1034	7__lociStitched	chr6	145089911	145116653	Irag2
NM_008512	217	13__lociStitched	chr10	127587974	127605182	Lrp1
NM_008517	1068	10__lociStitched	chr10	93430845	93447251	Lta4h
NM_008522	247	13__lociStitched	chr9	110994850	111046695	Ltf
NM_008535	766	5__lociStitched	chr8	84722516	84738118	Lyl1
NM_008551	1120	10__lociStitched	chr1	131072575	131111382	Mapkapk2
NM_008562	150	12__lociStitched	chr3	95631427	95676186	Mcl1
NM_008613	1039	7__lociStitched	chr9	72441399	72468930	Mns1
NM_008655	1047	7__lociStitched	chr10	80913918	80944168	Gadd45b
NM_008714	166	11__lociStitched	chr2	26471934	26494044	Notch1
NM_008773	499	8__lociStitched	chr7	101027926	101070570	P2ry2

NM_008774	153	11_ _lociStitched	chr15	36640322	36650335	Pabpc1
NM_008782	637	5_ _lociStitched	chr4	44705425	44714360	Pax5
NM_008816	976	7_ _lociStitched	chr11	106657853	106676047	Pecam1
NM_008842	129	12_ _lociStitched	chr17	29419144	29448353	Pim1
NM_008842	907	7_ _lociStitched	chr17	29493685	29503606	Pim1
NM_008845	828	5_ _lociStitched	chr2	18968975	18989324	Pip4k2a
NM_008846	650	5_ _lociStitched	chr19	24526681	24536117	Pip5k1b
NM_008855	539	5_ _lociStitched	chr7	122256354	122260704	Prkcb
NM_008855	45	17_ _lociStitched	chr7	122382928	122416864	Prkcb
NM_008855	890	7_ _lociStitched	chr7	122309477	122317925	Prkcb
NM_008879	218	13_ _lociStitched	chr14	75174348	75193158	Lcp1
NM_008898	1063	10_ _lociStitched	chr5	135702178	135714396	Por
NM_008913	215	11_ _lociStitched	chr3	136684147	136739237	Ppp3ca
NM_008960	563	5_ _lociStitched	chr19	32901988	32907566	Pten
NM_008972	47	17_ _lociStitched	chr1	86470066	86505908	Ptma
NM_008976	504	5_ _lociStitched	chr1	189799166	189801855	Ptpn14
NM_008982	38	18_ _lociStitched	chr2	90522377	90584102	Ptprj
NM_009006	601	5_ _lociStitched	chr19	6345655	6352805	Map4k2
NM_009008	93	14_ _lociStitched	chr15	78557961	78608201	Rac2
NM_009025	735	5_ _lociStitched	chr8	13673157	13687283	Rasa3
NM_009025	272	9_ _lociStitched	chr8	13643997	13660451	Rasa3
NM_009056	800	5_ _lociStitched	chr17	56819102	56836432	Rfx2
NM_009075	86	14_ _lociStitched	chr6	70716638	70754433	Rpia
NM_009112	512	5_ _lociStitched	chr3	93547234	93550601	S100a10
NM_009127	928	7_ _lociStitched	chr19	44376804	44389316	Scd1
NM_009128	430	8_ _lociStitched	chr19	44247679	44267139	Scd2
NM_009136	901	7_ _lociStitched	chr8	57429565	57438851	Scrg1
NM_009145	575	5_ _lociStitched	chr9	58657476	58663438	Nptn
NM_009151	36	18_ _lociStitched	chr5	113790516	113844857	Selplg
NM_009151	746	5_ _lociStitched	chr5	113858620	113873216	Selplg
NM_009177	283	9_ _lociStitched	chr15	67102981	67121483	St3gal1
NM_009177	335	9_ _lociStitched	chr15	67143048	67174457	St3gal1
NM_009179	212	11_ _lociStitched	chr8	110895550	110945054	St3gal2
NM_009192	463	8_ _lociStitched	chr15	66801267	66827466	Sla
NM_009203	528	5_ _lociStitched	chr19	6664637	6668752	Slc22a12
NM_009223	364	16_ _lociStitched	chr16	11055401	11093461	Snn
NM_009260	815	5_ _lociStitched	chr11	30176457	30195545	Sptbn1
NM_009288	102	15_ _lociStitched	chr11	32536945	32566315	Stk10
NM_009292	956	7_ _lociStitched	chr6	34938129	34953534	Stra8
NM_009296	139	12_ _lociStitched	chr11	87719307	87755379	Supt4a
NM_009320	427	8_ _lociStitched	chr6	91698418	91717196	Slc6a6
NM_009331	711	5_ _lociStitched	chr11	52266433	52279250	Tcf7
NM_009344	854	5_ _lociStitched	chr10	111515630	111540175	Phlda1

NM_009368	1113	10_ _lociStitched	chr12	85958216	85993093	Tgfb3
NM_009370	1050	7_ _lociStitched	chr4	47293778	47324891	Tgfbr1
NM_009373	91	14_ _lociStitched	chr2	158133364	158176967	Tgm2
NM_009387	323	9_ _lociStitched	chr11	117813825	117840877	Tk1
NM_009388	683	5_ _lociStitched	chr14	30551267	30562085	Tkt
NM_009389	118	12_ _lociStitched	chr9	61373612	61393681	Tle3
NM_009402	811	5_ _lociStitched	chr7	18873788	18892620	Pglyrp1
NM_009419	151	12_ _lociStitched	chr5	112265244	112311250	Tpst2
NM_009421	825	5_ _lociStitched	chr2	34957912	34977999	Traf1
NM_009464	258	9_ _lociStitched	chr7	100457746	100468673	Ucp3
NM_009500	216	13_ _lociStitched	chr2	27404385	27419010	Vav2
NM_009510	853	5_ _lociStitched	chr17	6804288	6828798	Ezr
NM_009531	992	7_ _lociStitched	chr6	91488630	91508400	Xpc
NM_009569	19	31_ _lociStitched	chr8	122273816	122331404	Zfpm1
NM_009578	347	9_ _lociStitched	chr11	11656405	11696316	Ikzf1
NM_009578	368	16_ _lociStitched	chr11	11715333	11763667	Ikzf1
NM_009581	533	5_ _lociStitched	chr1	130547248	130551452	Zp3r
NM_009609	941	7_ _lociStitched	chr11	120303144	120316776	Actg1
NM_009631	1052	7_ _lociStitched	chr3	105891369	105927027	Adora3
NM_009667	877	7_ _lociStitched	chr7	110774864	110781725	Ampd3
NM_009768	1041	7_ _lociStitched	chr10	79684609	79713158	Bsg
NM_009801	1072	10_ _lociStitched	chr3	14871518	14890082	Car2
NM_009805	654	5_ _lociStitched	chr1	58716210	58725956	Cflar
NM_009821	429	8_ _lociStitched	chr16	92699719	92718900	Runx1
NM_009824	203	11_ _lociStitched	chr8	122685141	122721514	Cbfa2t3
NM_009834	171	11_ _lociStitched	chr3	51208549	51233857	Noct
NM_009846	894	7_ _lociStitched	chr10	43580630	43589468	Cd24a
NM_009848	80	14_ _lociStitched	chr19	40663229	40694068	Entpd1
NM_009871	241	13_ _lociStitched	chr11	80479973	80523791	Cdk5r1
NM_009895	138	12_ _lociStitched	chr9	107278240	107313575	Cish
NM_009909	233	13_ _lociStitched	chr1	74149503	74181958	Cxcr2
NM_009911	24	33_ _lociStitched	chr1	128549079	128636699	Cxcr4
NM_009911	434	8_ _lociStitched	chr1	128704615	128724934	Cxcr4
NM_009912	100	15_ _lociStitched	chr9	123923108	123951776	Ccr1
NM_009946	78	14_ _lociStitched	chr13	54357574	54381877	Cplx2
NM_009946	959	7_ _lociStitched	chr13	54411859	54427486	Cplx2
NM_009983	101	15_ _lociStitched	chr7	142375224	142404261	Ctsd
NM_009987	1008	7_ _lociStitched	chr9	120037017	120059007	Cx3cr1
NM_010015	1062	10_ _lociStitched	chr14	54222317	54234211	Dad1
NM_010019	738	5_ _lociStitched	chr9	66200185	66214418	Dapk2
NM_010070	119	12_ _lociStitched	chr6	82996814	83017625	Dok1
NM_010119	201	11_ _lociStitched	chr19	6268317	6304058	Ehd1
NM_010121	852	5_ _lociStitched	chr6	70847229	70871375	Eif2ak3

NM_010160	762	5_ _lociStitched	chr2	6707482	6722991	Celf2
NM_010190	32	18_ _lociStitched	chr2	28063114	28100973	Fcnb
NM_010190	1100	10_ _lociStitched	chr2	27979307	28006129	Fcnb
NM_010208	92	14_ _lociStitched	chr4	132945342	132989022	Fgr
NM_010213	752	5_ _lociStitched	chr4	124695632	124710452	Fhl3
NM_010247	603	5_ _lociStitched	chr15	82021803	82028987	Xrcc6
NM_010266	861	5_ _lociStitched	chr19	21384145	21411601	Gda
NM_010266	666	5_ _lociStitched	chr19	21431983	21442158	Gda
NM_010283	895	7_ _lociStitched	chr2	35441242	35450160	Ggta1
NM_010283	918	7_ _lociStitched	chr2	35494948	35506779	Ggta1
NM_010286	108	15_ _lociStitched	chrX	140493376	140541373	Tsc22d3
NM_010302	193	11_ _lociStitched	chr5	140763407	140795210	Gna12
NM_010344	229	13_ _lociStitched	chr8	33646718	33676520	Gsr
NM_010346	1078	10_ _lociStitched	chr11	98457167	98477133	Grb7
NM_010380	906	7_ _lociStitched	chr17	35250378	35260190	H2-D1
NM_010382	1060	10_ _lociStitched	chr17	34293500	34302073	H2-Eb1
NM_010407	395	8_ _lociStitched	chr2	153115485	153129619	Hck
NM_010421	981	7_ _lociStitched	chr9	59534652	59553435	Hexa
NM_010442	631	5_ _lociStitched	chr8	75081267	75089642	Hmox1
NM_010444	180	11_ _lociStitched	chr15	101235579	101263187	Nr4a1
NM_010500	248	13_ _lociStitched	chr1	155038919	155091903	Ier5
NM_010509	437	8_ _lociStitched	chr16	91346652	91368015	Ifnar2
NM_010511	482	8_ _lociStitched	chr10	19562892	19594732	Ifngr1
NM_010566	362	16_ _lociStitched	chr1	87622769	87659784	Inpp5d
NM_010581	227	13_ _lociStitched	chr16	49790613	49819053	Cd47
NM_010657	943	7_ _lociStitched	chr4	119966075	119979836	Hivep3
NM_010686	301	9_ _lociStitched	chr4	130899550	130922132	Laptm5
NM_010696	808	5_ _lociStitched	chr11	34054183	34072454	Lcp2
NM_010709	271	9_ _lociStitched	chr1	131184590	131200841	Eif2d
NM_010721	721	5_ _lociStitched	chr18	56722688	56735891	Lmnb1
NM_010731	211	11_ _lociStitched	chr10	81128339	81173727	Zbtb7a
NM_010745	937	7_ _lociStitched	chr13	37501466	37514717	Ly86
NM_010748	369	16_ _lociStitched	chr13	13518124	13574553	Lyst
NM_010752	793	5_ _lociStitched	chr5	140207320	140224284	Mad1l1
NM_010777	165	11_ _lociStitched	chr18	82468951	82490928	Mbp
NM_010815	145	12_ _lociStitched	chr15	80599243	80640188	Grap2
NM_010831	210	11_ _lociStitched	chr17	31809437	31854321	Sik1
NM_010876	1022	7_ _lociStitched	chr5	134213142	134237846	Ncf1
NM_010877	103	15_ _lociStitched	chr1	152779316	152818485	Ncf2
NM_010901	942	7_ _lociStitched	chr8	106049788	106063436	Nfatc3
NM_010907	97	14_ _lociStitched	chr12	55433792	55496429	Nfkbia
NM_010928	418	8_ _lociStitched	chr3	97991691	98009704	Notch2
NM_010928	366	16_ _lociStitched	chr3	98045774	98086874	Notch2

NM_010939	550	5__lociStitched	chr1	62720709	62725451	Nrp2
NM_011050	998	7__lociStitched	chr19	53799902	53820262	Pdcd4
NM_011050	733	5__lociStitched	chr19	53886837	53900802	Pdcd4
NM_011061	452	8__lociStitched	chr4	140766228	140789347	Padi4
NM_011097	730	5__lociStitched	chr13	55891523	55905302	Pitx1
NM_011103	246	13__lociStitched	chr14	30593442	30642165	Prkcd
NM_011106	440	8__lociStitched	chr2	163697741	163719395	Pkig
NM_011113	124	12__lociStitched	chr7	24442026	24467416	Plaur
NM_011138	1016	7__lociStitched	chr7	25106946	25130364	Pou2f2
NM_011156	596	5__lociStitched	chr10	45100334	45107334	Prep
NM_011157	1082	10__lociStitched	chr10	62500731	62521351	Srgn
NM_011158	791	5__lociStitched	chr12	32001745	32018556	Prkar2b
NM_011158	302	9__lociStitched	chr12	32101182	32124021	Prkar2b
NM_011158	1006	7__lociStitched	chr12	32045539	32067100	Prkar2b
NM_011193	382	8__lociStitched	chr9	56097487	56109156	Pstpip1
NM_011201	1079	10__lociStitched	chr2	167941503	167961840	Ptpn1
NM_011202	804	5__lociStitched	chr5	121106853	121124622	Ptpn11
NM_011206	439	8__lociStitched	chr1	34464310	34485922	Ptpn18
NM_011223	17	23__lociStitched	chr5	115488493	115545608	Pxn
NM_011258	566	5__lociStitched	chr5	65321812	65327484	Rfc1
NM_011303	1105	10__lociStitched	chr4	145019018	145049217	Dhrs3
NM_011305	494	8__lociStitched	chr2	27690692	27727764	Rxra
NM_011338	602	5__lociStitched	chr11	83569187	83576341	Ccl9
NM_011346	297	9__lociStitched	chr1	164066194	164087917	Sell
NM_011352	466	8__lociStitched	chr9	57953413	57980707	Sema7a
NM_011355	401	8__lociStitched	chr2	91078098	91093131	Spi1
NM_011363	789	5__lociStitched	chr7	126466279	126483034	Sh2b1
NM_011385	731	5__lociStitched	chr4	155150328	155164126	Ski
NM_011403	416	8__lociStitched	chr11	102359750	102377588	Slc4a1
NM_011414	465	8__lociStitched	chr2	164359759	164386434	Slpi
NM_011436	1021	7__lociStitched	chr9	42084100	42108661	Sorl1
NM_011436	268	9__lociStitched	chr9	42019312	42034888	Sorl1
NM_011436	487	8__lociStitched	chr9	42125468	42158807	Sorl1
NM_011518	57	17__lociStitched	chr13	52565308	52620191	Syk
NM_011527	758	5__lociStitched	chr4	115047953	115063363	Tal1
NM_011528	312	9__lociStitched	chr7	141370266	141394694	Taldo1
NM_011539	152	12__lociStitched	chr6	38899457	38949828	Tbxas1
NM_011549	970	7__lociStitched	chr17	47753234	47770806	Tfeb
NM_011586	993	7__lociStitched	chr11	77783636	77803444	Myo18a
NM_011605	1045	7__lociStitched	chr10	91179675	91208929	Tmpo
NM_011609	136	12__lociStitched	chr6	125333815	125367922	Tnfrsf1a
NM_011610	1017	7__lociStitched	chr4	145254374	145277915	Tnfrsf1b
NM_011632	727	5__lociStitched	chr12	111180792	111194377	Traf3

NM_011633	917	7_ _lociStitched	chr1	192078138	192089850	Traf5
NM_011671	990	7_ _lociStitched	chr7	100484272	100503879	Ucp2
NM_011673	37	18_ _lociStitched	chr4	59231643	59287308	Ugcg
NM_011676	899	7_ _lociStitched	chr11	78344667	78353693	Unc119
NM_011695	708	5_ _lociStitched	chr14	21810739	21823518	Vdac2
NM_011705	1095	10_ _lociStitched	chr12	106016128	106040712	Vrk1
NM_011708	651	5_ _lociStitched	chr6	125564327	125573888	Vwf
NM_011711	673	5_ _lociStitched	chr15	99332442	99342862	Fmnl3
NM_011777	916	7_ _lociStitched	chr6	42343321	42354810	Zyx
NM_011799	421	8_ _lociStitched	chr11	98900753	98918940	Cdc6
NM_011808	244	13_ _lociStitched	chr9	32699383	32747723	Ets1
NM_011809	840	5_ _lociStitched	chr16	95707885	95729434	Ets2
NM_011817	391	8_ _lociStitched	chr13	51836738	51849886	Gadd45g
NM_011862	1096	10_ _lociStitched	chr15	83418768	83443824	Pacsin2
NM_011863	224	13_ _lociStitched	chr3	131465575	131491067	Papss1
NM_011893	1077	10_ _lociStitched	chr5	34519641	34539413	Sh3bp2
NM_011894	69	21_ _lociStitched	chr14	31386311	31432683	Sh3bp5
NM_011932	358	16_ _lociStitched	chr3	137940974	137975048	Dapp1
NM_011945	262	9_ _lociStitched	chr13	111928610	111942239	Map3k1
NM_011945	592	5_ _lociStitched	chr13	111977829	111984649	Map3k1
NM_011945	543	5_ _lociStitched	chr13	112000331	112004804	Map3k1
NM_011950	836	5_ _lociStitched	chr17	28736004	28757050	Mapk13
NM_011952	256	9_ _lociStitched	chr7	126744669	126755338	Mapk3
NM_011971	946	7_ _lociStitched	chr11	97715364	97729741	Psmb3
NM_012023	969	7_ _lociStitched	chr12	110488035	110505451	Ppp2r5c
NM_013469	264	9_ _lociStitched	chr14	25847801	25862439	Anxa11
NM_013486	479	8_ _lociStitched	chr3	101265879	101296592	Cd2
NM_013501	496	8_ _lociStitched	chr17	31722260	31761376	Cryaa
NM_013515	125	12_ _lociStitched	chr2	35307888	35334713	Stom
NM_013566	821	5_ _lociStitched	chr15	102224699	102244472	Itgb7
NM_013571	556	5_ _lociStitched	chr11	79117865	79123089	Ksr1
NM_013599	923	7_ _lociStitched	chr2	164938809	164950991	Mmp9
NM_013642	618	5_ _lociStitched	chr17	26530712	26538541	Dusp1
NM_013642	339	9_ _lociStitched	chr17	26481176	26515466	Dusp1
NM_013650	237	13_ _lociStitched	chr3	90657467	90693096	S100a8
NM_013659	170	11_ _lociStitched	chr7	80191168	80214919	Sema4b
NM_013660	7	19_ _lociStitched	chr13	51732704	51768891	Sema4d
NM_013687	255	9_ _lociStitched	chr17	28006357	28016822	Tcp11
NM_013711	955	7_ _lociStitched	chr16	18416042	18431317	Txnrd2
NM_013758	489	8_ _lociStitched	chr19	53162742	53196693	Add3
NM_013767	744	5_ _lociStitched	chr15	79432127	79446700	Csnk1e
NM_013818	958	7_ _lociStitched	chr15	79683061	79698631	Gtpbp1
NM_013842	126	12_ _lociStitched	chr11	5485621	5513273	Xbp1

NM_013867	394	8_ _lociStitched	chr3	122393874	122407526	Bcar3
NM_013880	279	9_ _lociStitched	chr17	50453323	50471111	Plcl2
NM_013918	660	5_ _lociStitched	chr16	77020233	77030221	Usp25
NM_015771	542	5_ _lociStitched	chr14	57768652	57773099	Lats2
NM_015818	1067	10_ _lociStitched	chr1	36087639	36103959	Hs6st1
NM_015818	628	5_ _lociStitched	chr1	36124209	36132472	Hs6st1
NM_016677	881	7_ _lociStitched	chr12	17710333	17717443	Hpcal1
NM_016696	850	5_ _lociStitched	chr1	92731595	92755076	Gpc1
NM_016713	774	5_ _lociStitched	chr11	70580398	70596532	Mink1
NM_016718	652	5_ _lociStitched	chr6	120149973	120159598	Ninj2
NM_016720	883	7_ _lociStitched	chr7	99818164	99825679	Neu3
NM_016721	186	11_ _lociStitched	chr7	80753246	80782875	Iqgap1
NM_016769	249	13_ _lociStitched	chr9	63678375	63741052	Smad3
NM_016775	814	5_ _lociStitched	chr2	181536230	181555216	Dnajc5
NM_016797	741	5_ _lociStitched	chr10	24158568	24173037	Stx7
NM_016803	285	9_ _lociStitched	chr10	60213268	60232492	Chst3
NM_016843	583	5_ _lociStitched	chr15	85354078	85360317	Atxn10
NM_016846	1030	7_ _lociStitched	chr1	152591813	152618005	Rgl1
NM_016888	604	5_ _lociStitched	chr11	22809245	22816458	B3gnt2
NM_016888	105	15_ _lociStitched	chr11	22830613	22873872	B3gnt2
NM_016896	219	13_ _lociStitched	chr11	103246185	103265056	Map3k14
NM_016957	700	5_ _lociStitched	chr4	133967541	133979756	Hmgn2
NM_017370	1012	7_ _lociStitched	chr8	109569964	109593037	Hp
NM_017372	461	8_ _lociStitched	chr10	117273612	117298860	Lyz2
NM_017373	458	8_ _lociStitched	chr13	52977016	53001297	Nfil3
NM_017375	409	8_ _lociStitched	chr19	18567616	18583875	Ostf1
NM_018745	756	5_ _lociStitched	chr15	38458588	38473831	Azin1
NM_018754	1115	10_ _lociStitched	chr4	133595652	133631198	Sfn
NM_018765	287	9_ _lociStitched	chr14	79516308	79536457	Wbp4
NM_018775	42	28_ _lociStitched	chr1	39408475	39504070	Tbc1d8
NM_018781	1085	10_ _lociStitched	chr14	70053838	70074904	Egr3
NM_018784	257	9_ _lociStitched	chr16	58497200	58508058	St3gal6
NM_018797	532	5_ _lociStitched	chr10	94843162	94847339	Plxnc1
NM_018797	177	11_ _lociStitched	chr10	94908823	94935511	Plxnc1
NM_018810	154	11_ _lociStitched	chr6	39422679	39436387	Mkrn1
NM_018815	785	5_ _lociStitched	chr6	91095146	91111743	Nup210
NM_019406	346	9_ _lociStitched	chr2	31108446	31148120	Fnbp1
NM_019426	1061	10_ _lociStitched	chr6	136463687	136474057	Atf7ip
NM_019547	3	25_ _lociStitched	chr2	173021844	173084418	Rbm38
NM_019552	930	7_ _lociStitched	chr8	123977960	123990718	Abcb10
NM_019566	423	8_ _lociStitched	chr7	102242038	102260426	Rhog
NM_019636	1107	10_ _lociStitched	chr5	64211418	64242111	Tbc1d1
NM_019661	355	16_ _lociStitched	chr11	5999616	6021259	Ykt6

NM_019680	133	12__lociStitched	chrX	48423965	48454792	Elf4
NM_019687	537	5__lociStitched	chr11	54003083	54007399	Slc22a4
NM_019702	669	5__lociStitched	chr10	21224356	21234715	Hbs1l
NM_019740	359	16__lociStitched	chr10	42244424	42278551	Foxo3
NM_019761	1028	7__lociStitched	chr2	148560320	148585814	Nxt1
NM_019777	696	5__lociStitched	chr1	131275927	131287685	Ikbke
NM_019835	191	11__lociStitched	chr2	167351019	167382517	B4galt5
NM_019866	915	7__lociStitched	chr7	44526757	44537375	Spib
NM_019872	882	7__lociStitched	chr4	107718439	107725686	Dmrtb1
NM_019965	5	25__lociStitched	chr10	59872958	59943865	Dnajb12
NM_019980	303	9__lociStitched	chr16	10957526	10980430	Litaf
NM_020260	773	5__lociStitched	chr16	38635919	38651985	Arhgap31
NM_020285	626	5__lociStitched	chr7	143057984	143066241	Tssc4
NM_020557	488	8__lociStitched	chr12	26452805	26486604	Cmpk2
NM_020559	555	5__lociStitched	chr9	106256855	106261948	Alas1
NM_021278	803	5__lociStitched	chrX	167195228	167212965	Tmsb4x
NM_021281	373	8__lociStitched	chr3	95516041	95523868	Ctss
NM_021349	975	7__lociStitched	chr11	61108825	61126941	Tnfrsf13b
NM_021366	25	33__lociStitched	chr7	63952738	64058763	Klf13
NM_021439	790	5__lociStitched	chr10	82957918	82974693	Chst11
NM_021439	43	17__lociStitched	chr10	82988666	83020296	Chst11
NM_021462	361	16__lociStitched	chr10	80663071	80699122	Mknk2
NM_021478	726	5__lociStitched	chr17	28407675	28421191	Tulp1
NM_021489	333	9__lociStitched	chr13	55419978	55450885	F12
NM_021493	83	14__lociStitched	chr11	97411991	97447247	Arhgap23
NM_021528	1076	10__lociStitched	chr5	140498045	140517623	Chst12
NM_021531	551	5__lociStitched	chr9	21554672	21559591	Carm1
NM_021540	425	8__lociStitched	chr11	49978451	49996975	Rnf130
NM_021606	565	5__lociStitched	chr2	38480899	38486526	Nek6
NM_021611	288	9__lociStitched	chr5	136622651	136642914	Myl10
NM_021887	104	15__lociStitched	chr7	125593838	125636559	Il21r
NM_022331	44	17__lociStitched	chr8	94346091	94379613	Herpud1
NM_022332	739	5__lociStitched	chr6	17741731	17756103	St7
NM_022410	23	33__lociStitched	chr15	77797981	77858123	Myh9
NM_022653	699	5__lociStitched	chr10	81076887	81088958	Thop1
NM_022656	944	7__lociStitched	chr14	31182098	31195940	Nisch
NM_022880	557	5__lociStitched	chr17	45580969	45586205	Slc29a1
NM_022984	181	11__lociStitched	chr8	3644706	3672411	Retn
NM_023049	926	7__lociStitched	chr12	103332034	103344412	Asb2
NM_023377	446	8__lociStitched	chr7	83639353	83661645	Stard5
NM_023409	788	5__lociStitched	chr12	84750937	84767591	Npc2
NM_023536	261	9__lociStitched	chr4	139330825	139344307	Mrto4
NM_023635	90	14__lociStitched	chr9	73047346	73090706	Rab27a

NM_023738	722	5_ _lociStitched	chr9	107970809	107984020	Uba7
NM_024177	644	5_ _lociStitched	chr11	116142461	116151566	Mrpl38
NM_024214	995	7_ _lociStitched	chr8	126788248	126808309	Tomm20
NM_024214	16	23_ _lociStitched	chr8	126820892	126867662	Tomm20
NM_024243	441	8_ _lociStitched	chr4	135897317	135919066	Fuca1
NM_024245	476	8_ _lociStitched	chr9	61954453	61984036	Kif23
NM_024454	778	5_ _lociStitched	chr10	115312419	115328760	Rab21
NM_025290	204	11_ _lociStitched	chr17	31254382	31291297	Rsph1
NM_025295	202	11_ _lociStitched	chr14	31661453	31697675	Btd
NM_025325	13	19_ _lociStitched	chr17	83770338	83832021	Haao
NM_025408	829	5_ _lociStitched	chr7	98321458	98341854	Acer3
NM_025411	530	5_ _lociStitched	chr4	135980979	135985131	Pithd1
NM_025427	252	9_ _lociStitched	chr14	79289621	79297377	Rgcc
NM_025483	675	5_ _lociStitched	chr16	56061810	56072252	Senp7
NM_025499	686	5_ _lociStitched	chr10	82889550	82900713	Eid3
NM_025508	719	5_ _lociStitched	chr13	45442488	45455645	Gmpr
NM_025569	822	5_ _lociStitched	chr1	167369060	167388904	Mgst3
NM_025601	891	7_ _lociStitched	chr8	13593499	13602093	1700029H14Rik
NM_025626	89	14_ _lociStitched	chr2	3710673	3753882	Fam107b
NM_025626	940	7_ _lociStitched	chr2	3655405	3669004	Fam107b
NM_025655	672	5_ _lociStitched	chr11	76879670	76890054	Tmigd1
NM_025774	885	7_ _lociStitched	chr5	136210481	136218488	Prkrip1
NM_025796	269	9_ _lociStitched	chr5	31596567	31612265	Mrpl33
NM_025806	985	7_ _lociStitched	chr6	136671909	136690915	Plbd1
NM_025821	121	12_ _lociStitched	chr16	8666190	8689438	Carhsp1
NM_025843	581	5_ _lociStitched	chr8	83572296	83578483	Ndufb7
NM_025892	228	13_ _lociStitched	chr16	32101688	32131057	Cep19
NM_025919	729	5_ _lociStitched	chr4	136077005	136090706	Rpl11
NM_025929	52	17_ _lociStitched	chr15	74897238	74936993	Ly6m
NM_025935	223	13_ _lociStitched	chr13	43195919	43219079	Tbc1d7
NM_025994	921	7_ _lociStitched	chr4	141860302	141872443	Efhd2
NM_026037	1071	10_ _lociStitched	chr12	24876696	24894489	Mboat2
NM_026062	475	8_ _lociStitched	chr5	107950579	107980055	Dipk1a
NM_026094	833	5_ _lociStitched	chr10	80533820	80554457	Atp8b3
NM_026097	448	8_ _lociStitched	chr11	82833900	82856407	Rffl
NM_026153	123	12_ _lociStitched	chr15	31332527	31357075	Ankrd33b
NM_026170	832	5_ _lociStitched	chr17	26601147	26621768	Ergic1
NM_026212	692	5_ _lociStitched	chr2	26596905	26608417	Agpat2
NM_026257	182	11_ _lociStitched	chr4	134096553	134124721	Ubxn11
NM_026290	277	9_ _lociStitched	chr17	28510645	28528113	Armc12
NM_026330	481	8_ _lociStitched	chr7	125472184	125503815	Nsmce1
NM_026376	924	7_ _lociStitched	chr6	116003441	116015722	Plxnd1
NM_026376	869	7_ _lociStitched	chr6	115968435	115973796	Plxnd1

NM_026414	46	17_ _lociStitched	chr6	86584421	86620114	Asprv1
NM_026514	784	5_ _lociStitched	chr17	79334800	79351385	Cdc42ep3
NM_026644	68	21_ _lociStitched	chr17	12149388	12187252	Agpat4
NM_026644	253	9_ _lociStitched	chr17	12201025	12210099	Agpat4
NM_026700	768	5_ _lociStitched	chr16	93706930	93722834	Dop1b
NM_026738	491	8_ _lociStitched	chr13	49189546	49225132	Card19
NM_026756	189	11_ _lociStitched	chr10	81404480	81435114	Nfic
NM_026775	172	11_ _lociStitched	chr12	85372060	85397750	Tmed10
NM_026785	408	8_ _lociStitched	chr2	164757955	164774054	Ube2c
NM_026830	1058	7_ _lociStitched	chr13	37619217	37658994	Rreb1
NM_026840	509	5_ _lociStitched	chr8	40962309	40965491	Pdgfrl
NM_026856	619	5_ _lociStitched	chr5	106703577	106711474	Zfp644
NM_026917	1110	10_ _lociStitched	chr9	123077740	123109474	Zdhhc3
NM_026942	240	13_ _lociStitched	chr9	58246059	58289093	Stoml1
NM_027064	1054	7_ _lociStitched	chr13	98996541	99033599	H2bl1
NM_027126	953	7_ _lociStitched	chr3	96487080	96502193	Hjv
NM_027129	236	13_ _lociStitched	chr2	131189018	131223797	Ap5s1
NM_027216	178	11_ _lociStitched	chr11	113536322	113563603	Slc39a11
NM_027230	484	8_ _lociStitched	chr2	165868866	165901255	Zmynd8
NM_027258	1057	7_ _lociStitched	chr11	116358929	116398462	Rnf157
NM_027450	267	9_ _lociStitched	chr4	43959627	43974984	Glipr2
NM_027450	659	5_ _lociStitched	chr4	43931303	43941175	Glipr2
NM_027456	987	7_ _lociStitched	chr1	86215048	86234281	Armc9
NM_027460	712	5_ _lociStitched	chr4	149779916	149792807	Slc25a33
NM_027521	393	8_ _lociStitched	chr10	80021891	80035113	Arhgap45
NM_027533	1048	7_ _lociStitched	chr3	102714714	102745032	Tspan2
NM_027572	709	5_ _lociStitched	chr10	40515035	40527819	Slc22a16
NM_027604	636	5_ _lociStitched	chr10	123199665	123208462	Usp15
NM_027711	313	9_ _lociStitched	chr13	95941173	95965633	Iqgap2
NM_027742	553	5_ _lociStitched	chr9	111142777	111147720	Lrrfip2
NM_027898	691	5_ _lociStitched	chr7	31148376	31159788	Gramd1a
NM_027904	678	5_ _lociStitched	chr16	30180677	30191259	Cpn2
NM_027966	1002	7_ _lociStitched	chr1	86423505	86444269	Tex44
NM_027972	474	8_ _lociStitched	chr1	172501706	172531149	Cfap45
NM_027975	971	7_ _lociStitched	chr2	158718393	158736220	Fam83d
NM_027978	81	14_ _lociStitched	chr5	100632491	100663832	Coq2
NM_027995	847	5_ _lociStitched	chr4	134474942	134498009	Paqr7
NM_028019	656	5_ _lociStitched	chr11	80168209	80178007	Rnf135
NM_028023	817	5_ _lociStitched	chr12	112821858	112841039	Cdca4
NM_028047	107	15_ _lociStitched	chr7	24367538	24414326	Smg9
NM_028071	88	14_ _lociStitched	chr8	119798474	119840666	Cotl1
NM_028075	740	5_ _lociStitched	chr15	82218653	82233040	Tnfrsf13c
NM_028079	728	5_ _lociStitched	chr13	62951619	62965210	Aopep

NM_028121	402	8_ _lociStitched	chr9	59280379	59295461	Adpgk
NM_028135	523	5_ _lociStitched	chr1	127511966	127515900	Tmem163
NM_028139	665	5_ _lociStitched	chr12	33211111	33221273	Atxn7l1
NM_028194	299	9_ _lociStitched	chr5	73188645	73210619	Fryl
NM_028197	406	8_ _lociStitched	chr10	62541316	62557160	Kifbp
NM_028312	305	9_ _lociStitched	chr9	110661980	110685038	Ccdc12
NM_028340	205	11_ _lociStitched	chr13	49240273	49278093	Susd3
NM_028443	1010	7_ _lociStitched	chr5	125052288	125074600	Rflna
NM_028451	690	5_ _lociStitched	chr11	57951385	57962733	Larp1
NM_028464	641	5_ _lociStitched	chr17	49431990	49441034	Mocs1
NM_028608	797	5_ _lociStitched	chr10	111986120	112003413	Glipr1
NM_028614	213	11_ _lociStitched	chr9	50802835	50854977	Ppp2r1b
NM_028637	919	7_ _lociStitched	chr2	132685141	132696996	Shld1
NM_028639	111	15_ _lociStitched	chr17	87286758	87345001	Ttc7
NM_028669	706	5_ _lociStitched	chr11	11557422	11570038	Spmip7
NM_028707	477	8_ _lociStitched	chr18	35913460	35943327	Psd2
NM_028716	538	5_ _lociStitched	chr2	34903445	34907769	Phf19
NM_028728	462	8_ _lociStitched	chr15	82987426	83013057	Nfam1
NM_028752	357	16_ _lociStitched	chr5	122213376	122244310	Hvcn1
NM_028773	786	5_ _lociStitched	chrX	48127753	48144355	Sash3
NM_028791	132	12_ _lociStitched	chr17	29657781	29688123	Cmtr1
NM_028862	780	5_ _lociStitched	chr11	44496025	44512452	Rnf145
NM_028864	988	7_ _lociStitched	chr6	38341546	38360840	Zc3hav1
NM_028941	112	12_ _lociStitched	chr8	117333799	117346096	Cmip
NM_028943	710	5_ _lociStitched	chr3	131357446	131370263	Sgms2
NM_028945	1103	10_ _lociStitched	chr14	27350406	27379935	Tasor
NM_028945	868	7_ _lociStitched	chr14	27400224	27405565	Tasor
NM_029083	307	9_ _lociStitched	chr10	59958804	59982135	Ddit4
NM_029116	929	7_ _lociStitched	chr8	14985578	14998171	Kbtbd11
NM_029364	520	5_ _lociStitched	chr10	121373189	121377038	Gns
NM_029441	643	5_ _lociStitched	chr8	116627387	116636471	Cdyl2
NM_029575	807	5_ _lociStitched	chr9	116366107	116384144	Tgfbr2
NM_029575	310	9_ _lociStitched	chr9	116122439	116146592	Tgfbr2
NM_029640	775	5_ _lociStitched	chr15	72971065	72987315	Trappc9
NM_029652	697	5_ _lociStitched	chr7	75774299	75786239	Klhl25
NM_029652	281	9_ _lociStitched	chr7	75854701	75873100	Klhl25
NM_029850	221	13_ _lociStitched	chr5	123313606	123334048	Bcl7a
NM_029935	259	9_ _lociStitched	chr7	132355807	132367407	Chst15
NM_029999	208	11_ _lociStitched	chr17	72925712	72967776	Lbh
NM_030101	141	12_ _lociStitched	chr7	68126979	68164017	Pgpep1l
NM_030209	87	14_ _lociStitched	chr8	120013280	120051542	Crispld2
NM_030251	688	5_ _lociStitched	chr6	88827728	88838938	Abtb1
NM_030599	977	7_ _lociStitched	chr6	128838109	128856453	Klrb1b

NM_030696	1042	7_ _lociStitched	chr11	120929778	120958354	Slc16a3
NM_030724	519	5_ _lociStitched	chr1	167268257	167272023	Uck2
NM_030732	909	7_ _lociStitched	chr3	21952797	21963087	Tbl1xr1
NM_030889	590	5_ _lociStitched	chr5	36372627	36379334	Sorcs2
NM_030890	903	7_ _lociStitched	chr17	34632079	34641561	Prrt1
NM_031172	321	9_ _lociStitched	chr11	58957323	58983364	Trim17
NM_031181	1043	7_ _lociStitched	chr7	43636710	43665390	Siglece
NM_031185	633	5_ _lociStitched	chr10	4120688	4129134	Akap12
NM_031257	40	18_ _lociStitched	chr8	25067201	25133498	Plekha2
NM_031376	318	9_ _lociStitched	chr19	41321257	41347003	Pik3ap1
NM_031376	493	8_ _lociStitched	chr19	41366130	41403023	Pik3ap1
NM_031869	630	5_ _lociStitched	chr5	116021370	116029659	Prkab1
NM_031874	783	5_ _lociStitched	chr9	21907585	21924159	Rab3d
NM_033374	535	5_ _lociStitched	chr11	34769492	34773751	Dock2
NM_033601	1025	7_ _lociStitched	chr7	19806612	19831581	Bcl3
NM_052994	541	5_ _lociStitched	chr10	60077641	60082029	Spock2
NM_052994	160	11_ _lociStitched	chr10	60151099	60171610	Spock2
NM_053091	377	8_ _lociStitched	chr2	152784801	152795531	Cox4i2
NM_053100	1112	10_ _lociStitched	chr19	46495173	46529113	Trim8
NM_053100	973	7_ _lociStitched	chr19	46454072	46471997	Trim8
NM_053149	238	13_ _lociStitched	chr4	46396178	46432959	Hemgn
NM_053166	846	5_ _lociStitched	chr11	48834161	48857013	Trim7
NM_053182	411	8_ _lociStitched	chr3	9878945	9895977	Pag1
NM_053202	338	9_ _lociStitched	chr6	99368196	99402090	Foxp1
NM_053268	568	5_ _lociStitched	chr9	96633976	96639758	Rasa2
NM_054043	564	5_ _lociStitched	chr11	88594548	88600138	Msi2
NM_054050	497	8_ _lociStitched	chr2	29622062	29662518	Rapgef1
NM_054097	516	5_ _lociStitched	chr10	127202994	127206617	Pip4k2c
NM_058212	663	5_ _lociStitched	chr12	83308536	83318636	Dpf3
NM_058212	671	5_ _lociStitched	chr12	83424505	83434885	Dpf3
NM_080289	250	9_ _lociStitched	chr4	44938197	44944720	Grhpr
NM_080289	892	7_ _lociStitched	chr4	44962550	44971283	Grhpr
NM_080443	536	5_ _lociStitched	chr7	66675218	66679510	Asb7
NM_080510	681	5_ _lociStitched	chr2	122153755	122164531	Trim69
NM_080553	661	5_ _lociStitched	chr17	27073756	27083752	Itpr3
NM_080561	222	13_ _lociStitched	chr5	143038118	143060165	Rnf216
NM_080708	1026	7_ _lociStitched	chr5	96905892	96931044	Bmp2k
NM_080708	560	5_ _lociStitched	chr5	96948669	96954072	Bmp2k
NM_130796	982	7_ _lociStitched	chr13	113599774	113618586	Snx18
NM_130863	161	11_ _lociStitched	chr19	4285550	4306317	Grk2
NM_133198	410	8_ _lociStitched	chr12	70218599	70235305	Pygl
NM_133501	960	7_ _lociStitched	chr2	29179684	29195375	Ntng2
NM_133659	33	18_ _lociStitched	chr16	95426923	95465463	Erg

NM_133661	599	5_ _lociStitched	chr6	121336784	121343896	Slc6a12
NM_133665	1013	7_ _lociStitched	chr3	88127900	88150998	Mef2d
NM_133685	167	11_ _lociStitched	chr17	65718824	65741050	Rab31
NM_133724	1018	7_ _lociStitched	chr5	36712796	36736470	Bloc1s4
NM_133737	1104	10_ _lociStitched	chr6	57743807	57773828	Lancl2
NM_133739	961	7_ _lociStitched	chr9	7774439	7790457	Tmem123
NM_133757	67	20_ _lociStitched	chr11	117946369	118016801	Pgs1
NM_133765	777	5_ _lociStitched	chr8	121569911	121586188	Fbxo31
NM_133809	552	5_ _lociStitched	chr1	175640607	175645540	Kmo
NM_133815	329	9_ _lociStitched	chr1	181852332	181881693	Lbr
NM_133835	372	8_ _lociStitched	chr2	26011143	26018620	Ubac1
NM_133867	611	5_ _lociStitched	chr3	107845858	107853372	Eps8l3
NM_133867	834	5_ _lociStitched	chr3	107812262	107833115	Eps8l3
NM_133895	705	5_ _lociStitched	chr5	127608721	127621255	Slc15a4
NM_133910	464	8_ _lociStitched	chr5	36549422	36575704	Tbc1d14
NM_133919	53	17_ _lociStitched	chr5	103721619	103763253	Aff1
NM_133933	1117	10_ _lociStitched	chr6	88037677	88075484	Rpn1
NM_133999	196	11_ _lociStitched	chr10	41189286	41222858	Fig4
NM_134083	594	5_ _lociStitched	chr14	73116285	73123183	Rcbtb2
NM_134086	948	7_ _lociStitched	chr15	96592945	96607502	Slc38a1
NM_134091	1029	7_ _lociStitched	chr15	81020673	81046485	Sgsm3
NM_134131	164	11_ _lociStitched	chr18	50016306	50037926	Tnfaip8
NM_134148	984	7_ _lociStitched	chr19	4169549	4188446	Carns1
NM_134156	880	7_ _lociStitched	chr12	80228290	80235284	Actn1
NM_134189	870	7_ _lociStitched	chr11	57650673	57656183	Galnt10
NM_138306	436	8_ _lociStitched	chr2	91965133	91986490	Dgkz
NM_138313	1040	7_ _lociStitched	chr2	118533849	118562302	Bmf
NM_138601	324	9_ _lociStitched	chr10	78133819	78161291	Gatd3a
NM_138650	689	5_ _lociStitched	chr16	22608213	22619468	Dgkg
NM_138664	1114	10_ _lociStitched	chr16	91601491	91636875	Dnajc28
NM_138665	966	7_ _lociStitched	chr2	27295796	27312445	Sardh
NM_138950	802	5_ _lociStitched	chr11	75443687	75461305	Wdr81
NM_138956	76	14_ _lociStitched	chr10	121486341	121508038	Rassf3
NM_138956	1073	10_ _lociStitched	chr10	121411936	121430562	Rassf3
NM_139065	389	8_ _lociStitched	chr5	66066874	66079776	Rbm47
NM_139145	317	9_ _lociStitched	chr16	94221943	94247582	Hlcs
NM_139198	184	11_ _lociStitched	chr5	100557301	100586142	Plac8
NM_139272	380	8_ _lociStitched	chr8	124271082	124282387	Galnt2
NM_144516	591	5_ _lociStitched	chr13	9872649	9879424	Zmynd11
NM_144538	662	5_ _lociStitched	chr19	10013293	10023346	Rab3il1
NM_144549	445	8_ _lociStitched	chr15	59706776	59729045	Trib1
NM_144788	319	9_ _lociStitched	chr12	51841995	51867808	Hectd1
NM_144797	732	5_ _lociStitched	chr11	121734491	121748293	Metrnl

NM_144846	315	9_ _lociStitched	chr15	64075519	64100631	Cyrib
NM_144846	1101	10_ _lociStitched	chr15	63997447	64024434	Cyrib
NM_144854	757	5_ _lociStitched	chr16	87610843	87626239	Map3k7cl
NM_145067	1009	7_ _lociStitched	chr6	136713941	136736021	Gucy2c
NM_145155	680	5_ _lociStitched	chr5	146307118	146317860	Wasf3
NM_145158	116	12_ _lociStitched	chr17	71284785	71301075	Emilin2
NM_145158	1065	10_ _lociStitched	chr17	71256799	71271918	Emilin2
NM_145376	14	19_ _lociStitched	chr13	73433673	73500663	Lpcat1
NM_145383	848	5_ _lociStitched	chr6	115886612	115909844	Rho
NM_145458	1090	10_ _lociStitched	chr14	8093649	8116467	Pxk
NM_145465	175	11_ _lociStitched	chr14	121347344	121373367	Stk24
NM_145475	195	11_ _lociStitched	chr15	86172199	86204764	Cerk
NM_145500	963	7_ _lociStitched	chr19	41989737	42006052	Ubtd1
NM_145510	999	7_ _lociStitched	chr1	134507593	134528020	Rabif
NM_145514	367	16_ _lociStitched	chr1	181216779	181262350	Wdr26
NM_145522	849	5_ _lociStitched	chr2	34782634	34806005	Rabepk
NM_145536	794	5_ _lociStitched	chr2	153076019	153093078	Ccm2l
NM_145554	35	18_ _lociStitched	chr4	134728840	134773421	Ldlrap1
NM_145587	392	8_ _lociStitched	chr7	126276189	126289387	Sbk1
NM_145605	417	8_ _lociStitched	chr8	121788975	121806934	Klhdc4
NM_145823	56	17_ _lociStitched	chr11	107397557	107451927	Pitpnc1
NM_145824	84	14_ _lociStitched	chr8	105798476	105833836	Ranbp10
NM_145836	140	12_ _lociStitched	chr12	86800940	86837103	Irf2bpl
NM_145836	431	8_ _lociStitched	chr12	86875851	86895455	Irf2bpl
NM_145853	292	9_ _lociStitched	chr5	120559036	120579882	Tpcn1
NM_145857	424	8_ _lociStitched	chr8	88651075	88669565	Nod2
NM_145927	617	5_ _lociStitched	chr12	76886482	76894197	Fntb
NM_145928	149	12_ _lociStitched	chr14	40919076	40963801	Tspan14
NM_145959	545	5_ _lociStitched	chr15	58591176	58595730	Fam91a1
NM_145964	653	5_ _lociStitched	chr3	127815437	127825105	Ap1ar
NM_145968	887	7_ _lociStitched	chr17	7874417	7882465	Tagap
NM_145976	6	19_ _lociStitched	chr13	56151699	56185525	Tifab
NM_146001	134	12_ _lociStitched	chr5	135460277	135493394	Hip1
NM_146118	677	5_ _lociStitched	chr2	32486075	32496575	Slc25a25
NM_146119	845	5_ _lociStitched	chr2	32881370	32904116	Niban2
NM_146145	449	8_ _lociStitched	chr4	101170326	101192978	Jak1
NM_146173	876	7_ _lociStitched	chr6	29696832	29703687	Tspan33
NM_146184	226	13_ _lociStitched	chr7	25624715	25652565	B3gnt8
NM_146191	991	7_ _lociStitched	chr7	66304264	66323963	Lrrk1
NM_146226	472	8_ _lociStitched	chr9	108082348	108111432	Apeh
NM_146680	624	5_ _lociStitched	chr19	12005469	12013665	Or4d11
NM_148933	98	15_ _lociStitched	chr2	180435339	180456650	Slco4a1
NM_148947	510	5_ _lociStitched	chr17	35102523	35105737	Ly6g5c

NM_152810	384	8_ _lociStitched	chr17	45290828	45302736	Cdc5l
NM_153068	682	5_ _lociStitched	chr7	15953676	15964455	Ehd2
NM_153104	939	7_ _lociStitched	chr11	95796462	95809954	Phospho1
NM_153119	26	24_ _lociStitched	chr9	65527882	65577822	Plekho2
NM_153138	275	9_ _lociStitched	chr2	73453162	73470565	Wipf1
NM_153178	2	25_ _lociStitched	chr15	73130841	73182805	Ago2
NM_153382	609	5_ _lociStitched	chr14	57707300	57714691	Lats2
NM_153408	291	9_ _lociStitched	chr1	36261806	36282641	Neurl3
NM_153423	750	5_ _lociStitched	chr4	133079155	133093892	Wasf2
NM_153576	115	12_ _lociStitched	chr7	25424998	25440983	Cxcl17
NM_153776	29	18_ _lociStitched	chr12	113222532	113245687	Tmem121
NM_153794	645	5_ _lociStitched	chr18	68189983	68199165	Fam210a
NM_170758	21	22_ _lociStitched	chr11	114872837	114931904	Cd300a
NM_170777	117	12_ _lociStitched	chr9	22107107	22126327	Elof1
NM_172258	351	9_ _lociStitched	chr11	55106212	55155719	Slc36a3
NM_172261	225	13_ _lociStitched	chr11	94987545	95013200	Ppp1r9b
NM_172279	648	5_ _lociStitched	chr7	19466097	19475423	Mark4
NM_172285	1108	10_ _lociStitched	chr8	117512976	117543973	Plcg2
NM_172406	234	13_ _lociStitched	chr1	58937596	58970700	Trak2
NM_172415	978	7_ _lociStitched	chr4	140566520	140584887	Arhgef10l
NM_172424	187	11_ _lociStitched	chr5	118465097	118495340	Med13l
NM_172424	1015	7_ _lociStitched	chr5	118635983	118659271	Med13l
NM_172468	576	5_ _lociStitched	chr4	59814130	59820092	Snx30
NM_172524	588	5_ _lociStitched	chr11	46122487	46129028	Nipal4
NM_172543	147	12_ _lociStitched	chr11	95328328	95370644	Fam117a
NM_172589	627	5_ _lociStitched	chr13	93989516	93997775	Lhfpl2
NM_172599	1035	7_ _lociStitched	chr14	47514919	47541777	Atg14
NM_172606	620	5_ _lociStitched	chr15	31534919	31542819	Marchf6
NM_172607	1102	10_ _lociStitched	chr15	75875657	75905146	Naprt
NM_172612	819	5_ _lociStitched	chr15	98673045	98692688	Rnd1
NM_172661	1088	10_ _lociStitched	chr2	32115456	32137967	Prrc2b
NM_172688	265	9_ _lociStitched	chr4	32163778	32178437	Map3k7
NM_172704	570	5_ _lociStitched	chr4	151956435	151962262	Dnajc11
NM_172707	1036	7_ _lociStitched	chr5	32410763	32437873	Ppp1cb
NM_172723	818	5_ _lociStitched	chr5	139284879	139304321	Adap1
NM_172725	1083	10_ _lociStitched	chr5	142439781	142460531	Ap5z1
NM_172728	962	7_ _lociStitched	chr6	53446414	53462474	Creb5
NM_172874	816	5_ _lociStitched	chr4	108064588	108083687	Podn
NM_172884	851	5_ _lociStitched	chr5	113113926	113137837	2900026A02Rik
NM_172938	884	7_ _lociStitched	chr10	42894609	42902220	Scml4
NM_173363	298	9_ _lociStitched	chr12	111514285	111536210	Eif5
NM_173371	763	5_ _lociStitched	chr4	150000591	150016153	H6pd
NM_173405	809	5_ _lociStitched	chr5	140731312	140749752	Amz1

NM_173413	340	9__lociStitched	chr9	66882369	66917610	Rab8b
NM_174989	1044	7__lociStitched	chr17	56264694	56293451	Ticam1
NM_175121	348	9__lociStitched	chr15	96736717	96778410	Slc38a2
NM_175121	606	5__lociStitched	chr15	96709013	96716247	Slc38a2
NM_175134	453	8__lociStitched	chr15	36448296	36471892	Ankrd46
NM_175256	866	7__lociStitched	chr16	33731365	33736134	Heg1
NM_175268	15	39__lociStitched	chr7	132672144	132740087	Fam53b
NM_175268	242	13__lociStitched	chr7	132766459	132810721	Fam53b
NM_175272	501	5__lociStitched	chr7	49320303	49322785	Nav2
NM_175274	898	7__lociStitched	chr5	140636713	140645738	Ttyh3
NM_175279	647	5__lociStitched	chr7	113037304	113046564	Rassf10
NM_175349	243	13__lociStitched	chr17	5286212	5332042	Ldhal6b
NM_175353	407	8__lociStitched	chr19	37546545	37562582	Exoc6
NM_175367	912	7__lociStitched	chr12	91756889	91767295	Ston2
NM_175403	415	8__lociStitched	chr5	115150278	115168063	Mlec
NM_175423	1093	10__lociStitched	chr5	123017251	123040825	Orai1
NM_175433	109	15__lociStitched	chr7	80014928	80064747	Zfp710
NM_175445	337	9__lociStitched	chr2	132006385	132040265	Rassf2
NM_175451	1121	10__lociStitched	chr10	84488595	84530943	Ckap4
NM_175470	508	5__lociStitched	chr3	108166093	108169173	Gpr61
NM_175476	567	5__lociStitched	chr6	87498947	87504667	Arhgap25
NM_175493	767	5__lociStitched	chr12	100922681	100938345	Gpr68
NM_175493	725	5__lociStitched	chr12	100954202	100967716	Gpr68
NM_175511	896	7__lociStitched	chr2	32057007	32065960	Fam78a
NM_175522	860	5__lociStitched	chr5	140010530	140035910	Elfn1
NM_175645	314	9__lociStitched	chr7	117470024	117495037	Xylt1
NM_175751	1051	7__lociStitched	chr18	54957781	54993125	Zfp608
NM_175751	254	9__lociStitched	chr18	54899314	54908836	Zfp608
NM_176902	443	8__lociStitched	chr11	116417989	116440128	Ubald2
NM_177073	322	9__lociStitched	chr7	100843900	100870570	Relt
NM_177089	546	5__lociStitched	chr8	25191238	25195837	Tacc1
NM_177099	400	8__lociStitched	chr1	180875527	180890422	Lefty2
NM_177260	194	11__lociStitched	chr3	84654138	84686166	Tmem154
NM_177301	1046	7__lociStitched	chr7	28792114	28821538	Hnrnpl
NM_177304	950	7__lociStitched	chr8	46928562	46943509	Enpp6
NM_177304	561	5__lociStitched	chr8	46887550	46893040	Enpp6
NM_177305	827	5__lociStitched	chr1	88635870	88656040	Arl4c
NM_177320	920	7__lociStitched	chr11	68439901	68452027	Pik3r5
NM_177376	502	5__lociStitched	chr2	70090526	70093186	Myo3b
NM_177460	782	5__lociStitched	chr9	65186175	65202658	Parp16
NM_177547	635	5__lociStitched	chr1	9775803	9784528	Sgk3
NM_177589	996	7__lociStitched	chr9	121297608	121317744	Ulk4
NM_177604	527	5__lociStitched	chr1	130740168	130744240	AA986860

NM_177632	934	7_ _lociStitched	chr16	30595390	30608494	Fam43a
NM_177646	548	5_ _lociStitched	chr1	87846274	87850930	Dgkd
NM_177683	471	8_ _lociStitched	chr6	114873461	114901821	Vgll4
NM_177733	282	9_ _lociStitched	chr4	136172316	136190804	E2f2
NM_177771	1019	7_ _lociStitched	chr9	110440389	110464111	Klhl18
NM_177780	1004	7_ _lociStitched	chr14	67868828	67890135	Dock5
NM_177782	886	7_ _lociStitched	chr2	166650243	166658271	Prex1
NM_177782	614	5_ _lociStitched	chr2	166702904	166710562	Prex1
NM_177782	387	8_ _lociStitched	chr2	166619367	166632058	Prex1
NM_177785	1001	7_ _lociStitched	chr4	42860288	42880929	Spata31f3
NM_177876	63	20_ _lociStitched	chr5	124011422	124060542	Vps37b
NM_178045	1074	10_ _lociStitched	chr6	116664373	116683517	Rassf4
NM_178060	607	5_ _lociStitched	chr11	98747852	98755114	Thra
NM_178114	397	8_ _lociStitched	chr15	97458060	97472552	Amigo2
NM_178149	748	5_ _lociStitched	chr11	3334809	3349510	Pik3ip1
NM_178220	432	8_ _lociStitched	chr7	99531251	99550954	Arrb1
NM_178357	715	5_ _lociStitched	chr12	24633376	24646352	Klf11
NM_178398	945	7_ _lociStitched	chr5	142637412	142651369	Wipi2
NM_178593	110	15_ _lociStitched	chr1	165662535	165719080	Rcsd1
NM_178606	309	9_ _lociStitched	chr10	67025449	67049437	Reep3
NM_178635	771	5_ _lociStitched	chr7	99087019	99102950	Uvrag
NM_178635	352	9_ _lociStitched	chr7	99121085	99174251	Uvrag
NM_178763	413	8_ _lociStitched	chr11	121542972	121560405	Zfp750
NM_178772	936	7_ _lociStitched	chr3	27209704	27222919	Nceh1
NM_178874	1087	10_ _lociStitched	chr1	132366816	132389061	Tmcc2
NM_178886	308	9_ _lociStitched	chr2	102034495	102058422	Ldlrad3
NM_178893	214	11_ _lociStitched	chr4	46547001	46599302	Coro2a
NM_178901	949	7_ _lociStitched	chr7	127081774	127096382	AI467606
NM_178931	657	5_ _lociStitched	chr4	154934523	154944368	Tnfrsf14
NM_181321	332	9_ _lociStitched	chr11	117763030	117792878	Tmc6
NM_181397	435	8_ _lociStitched	chr17	50050804	50071308	Rftn1
NM_181397	888	7_ _lociStitched	chr17	50018994	50027240	Rftn1
NM_181403	414	8_ _lociStitched	chr19	10691879	10709476	Vps37c
NM_181413	720	5_ _lociStitched	chr17	27925359	27938549	Anks1
NM_181421	197	11_ _lociStitched	chr5	121233503	121267197	Hectd4
NM_181819	578	5_ _lociStitched	chr11	94259804	94265827	Wfikkn2
NM_182785	781	5_ _lociStitched	chr7	24837726	24854170	Lypd4
NM_182806	586	5_ _lociStitched	chr14	121903252	121909682	Gpr18
NM_183031	66	20_ _lociStitched	chr14	121928821	121995604	Gpr183
NM_183161	927	7_ _lociStitched	chr2	180728056	180740450	Slc17a9
NM_183175	10	19_ _lociStitched	chr14	60708984	60758577	C1qtnf9
NM_183208	190	11_ _lociStitched	chr14	25458937	25489642	Zmiz1
NM_183263	623	5_ _lociStitched	chr11	76260795	76268989	Mrm3

NM_183318	144	12_ _lociStitched	chrX	102035690	102075395	Rtl5
NM_183426	341	9_ _lociStitched	chr10	80070946	80106499	Sbno2
NM_183428	428	8_ _lociStitched	chr4	132059802	132078919	Epb41
NM_194334	20	22_ _lociStitched	chr9	90232129	90275814	Tbc1d2b
NM_194355	266	9_ _lociStitched	chr18	67560477	67575319	Spire1
NM_198109	74	14_ _lociStitched	chr5	113972523	113990216	Ssh1
NM_198414	873	7_ _lociStitched	chr9	95550785	95557446	Paqr9
NM_198423	863	5_ _lociStitched	chr11	120209283	120237721	Bahcc1
NM_198438	862	5_ _lociStitched	chr4	106907739	106935671	Ssbp3
NM_198604	813	5_ _lociStitched	chr6	125426521	125445480	Plekhg6
NM_198626	403	8_ _lociStitched	chr16	30961020	30976228	Xxylt1
NM_198631	826	5_ _lociStitched	chr7	16398673	16418839	Zc3h4
NM_198642	370	16_ _lociStitched	chr14	75009517	75069883	Rubcnl
NM_198656	438	8_ _lociStitched	chr2	178544401	178565947	Cdh26
NM_198664	1111	10_ _lociStitched	chr4	46613853	46646257	Tbc1d2
NM_198671	1089	10_ _lociStitched	chr8	120475295	120498081	Gse1
NM_198671	300	9_ _lociStitched	chr8	120322853	120345031	Gse1
NM_198671	220	13_ _lociStitched	chr8	120363771	120383628	Gse1
NM_199068	983	7_ _lociStitched	chr5	142366739	142385635	Foxk1
NM_199197	547	5_ _lociStitched	chr18	80193088	80197694	Rbfa
NM_199299	968	7_ _lociStitched	chr11	51831947	51848997	Jade2
NM_199309	11	19_ _lociStitched	chr16	32502095	32551788	Zdhhc19
NM_201351	612	5_ _lociStitched	chr19	10582226	10589774	Cyb561a3
NM_207219	459	8_ _lociStitched	chr17	48404272	48428786	Oard1
NM_207277	490	8_ _lociStitched	chr5	34896502	34931379	Msantd1
NM_211357	336	9_ _lociStitched	chr4	132590651	132624420	Eya3
NM_212487	879	7_ _lociStitched	chr15	101974425	101981324	Krt78
NR_002847	85	14_ _lociStitched	chr19	5786514	5823605	Malat1
NR_003523	334	9_ _lociStitched	chr15	62159006	62190141	Macroh2a3
NR_003623	1075	10_ _lociStitched	chr1	180386858	180406254	Gm5069
NR_003627	507	5_ _lociStitched	chr15	95960972	95964038	D030018L15Rik
NR_015500	34	18_ _lociStitched	chr2	167739879	167778820	A530013C23Rik
NR_015500	122	12_ _lociStitched	chr2	167693571	167717702	A530013C23Rik
NR_015540	986	7_ _lociStitched	chr1	89594545	89613664	4933400F21Rik
NR_015540	979	7_ _lociStitched	chr1	89562917	89581385	4933400F21Rik
NR_015548	207	11_ _lociStitched	chr7	135628229	135667441	5830432E09Rik
NR_015614	22	22_ _lociStitched	chr7	90020315	90083746	E230029C05Rik
NR_015618	874	7_ _lociStitched	chr17	35997681	36004359	A930015D03Rik
NR_026944	698	5_ _lociStitched	chr10	77220964	77232925	Gm10941
NR_027488	754	5_ _lociStitched	chr16	22047240	22062417	Senp2
NR_027666	772	5_ _lociStitched	chr1	170918974	170934966	Fcrla
NR_027893	655	5_ _lociStitched	chr13	52822566	52832349	BB123696
NR_028125	713	5_ _lociStitched	chr8	77478910	77491828	0610038B21Rik

NR_028264	525	5_ _lociStitched	chr14	61968170	61972122	Dleu2
NR_028427	1091	10_ _lociStitched	chr19	53439505	53462429	Mirt1
NR_028590	480	8_ _lociStitched	chr2	128415003	128446077	Morrbid
NR_029457	843	5_ _lociStitched	chrX	169997350	170019194	G530011O06Rik
NR_029532	158	11_ _lociStitched	chr6	31107572	31126974	Mir29b-1
NR_029556	952	7_ _lociStitched	chr11	78060876	78075943	Mir144
NR_029729	350	9_ _lociStitched	chr15	85634479	85679671	Mirlet7c-2
NR_029791	64	20_ _lociStitched	chr7	101453210	101504712	Mir139
NR_029804	517	5_ _lociStitched	chr15	82183890	82187600	Mir33
NR_030450	867	7_ _lociStitched	chr12	69756423	69761485	Mir681
NR_030455	577	5_ _lociStitched	chr4	11123448	11129448	Mir684-2
NR_030500	670	5_ _lociStitched	chr5	136393732	136404093	Mir721
NR_030571	320	9_ _lociStitched	chr11	86385353	86411187	Mir467c
NR_030675	649	5_ _lociStitched	chr15	79891573	79900941	D730005E14Rik
NR_030699	761	5_ _lociStitched	chr5	20940686	20956130	A630072M18Rik
NR_030762	130	12_ _lociStitched	chr2	144218914	144248411	Snord17
NR_033133	922	7_ _lociStitched	chr11	69843212	69855359	Plscr3
NR_033218	230	13_ _lociStitched	chr13	37853030	37883448	Rreb1
NR_033492	345	9_ _lociStitched	chr14	25577380	25615595	4931406H21Rik
NR_033493	1007	7_ _lociStitched	chr16	32650826	32672628	Tnk2os
NR_033496	498	8_ _lociStitched	chr4	107742882	107783922	Lrp8
NR_033558	787	5_ _lociStitched	chr10	43625740	43642386	F930017D23Rik
NR_034051	503	5_ _lociStitched	chr15	98510707	98513391	Snora34
NR_035421	8	19_ _lociStitched	chr12	100983419	101026503	Mir1190
NR_035430	932	7_ _lociStitched	chr12	55377474	55390444	Mir1198
NR_035457	897	7_ _lociStitched	chr12	102689774	102698735	Mir1936
NR_035488	174	11_ _lociStitched	chr7	135579837	135605671	Mir1962
NR_036598	1123	10_ _lociStitched	chr12	110966020	111010638	6030440G07Rik
NR_037236	173	11_ _lociStitched	chr14	25504645	25530430	Mir3075
NR_037272	518	5_ _lociStitched	chr2	30689946	30693708	Mir3089
NR_037569	55	17_ _lociStitched	chr7	73568847	73619323	Chaserr
NR_038011	468	8_ _lociStitched	chr1	151074953	151102374	C730036E19Rik
NR_038019	638	5_ _lociStitched	chr10	99458763	99467729	Gm20110
NR_038091	668	5_ _lociStitched	chr16	24593304	24603571	Morf4l1-ps1
NR_038130	589	5_ _lociStitched	chr12	99284258	99290804	4930474N09Rik
NR_039553	263	9_ _lociStitched	chr11	75559441	75573584	Mir3971
NR_039573	760	5_ _lociStitched	chr15	80896502	80911936	Mir5113
NR_039582	296	9_ _lociStitched	chr4	133377668	133399202	Mir5122
NR_040340	554	5_ _lociStitched	chr7	68282349	68287400	Altre
NR_040343	1119	10_ _lociStitched	chr7	80628827	80667538	Gm15880
NR_040385	747	5_ _lociStitched	chr3	41351195	41365803	Platr4
NR_040403	701	5_ _lociStitched	chr3	89378483	89390710	Gm15417
NR_040409	447	8_ _lociStitched	chr3	96555143	96577610	Gm15441

NR_040449	96	14_ _lociStitched	chr19	58086416	58144357	Gm10007
NR_040485	931	7_ _lociStitched	chr16	95827184	95840118	1600002D24Rik
NR_040486	872	7_ _lociStitched	chr16	93125122	93131627	1810053B23Rik
NR_040518	311	9_ _lociStitched	chr16	10998719	11023025	Gm4262
NR_040552	513	5_ _lociStitched	chr3	127929927	127933359	9830132P13Rik
NR_040581	935	7_ _lociStitched	chr10	43814728	43827903	1700027J07Rik
NR_040581	106	15_ _lociStitched	chr10	43722711	43766530	1700027J07Rik
NR_040618	684	5_ _lociStitched	chr2	35830853	35841687	9030204H09Rik
NR_040627	837	5_ _lociStitched	chr1	184644342	184665464	4930488B22Rik
NR_040679	1003	7_ _lociStitched	chr5	100217503	100238782	2310034O05Rik
NR_040681	200	11_ _lociStitched	chr5	64376460	64412108	1700027F09Rik
NR_040686	460	8_ _lociStitched	chr5	148954897	148979731	8430423G03Rik
NR_045069	245	13_ _lociStitched	chr15	79720470	79768902	Gm16576
NR_045078	486	8_ _lociStitched	chr5	64567723	64600348	Gm3716
NR_045079	724	5_ _lociStitched	chr17	5801863	5815371	3300005D01Rik
NR_045163	353	16_ _lociStitched	chr6	72100613	72120110	4933431G14Rik
NR_045165	857	5_ _lociStitched	chr6	72139601	72164847	4933431G14Rik
NR_045190	974	7_ _lociStitched	chr4	129909048	129927024	E330017L17Rik
NR_045288	616	5_ _lociStitched	chr12	99639870	99647573	1700064M15Rik
NR_045294	893	7_ _lociStitched	chr14	75829010	75837772	Gm4285
NR_045313	839	5_ _lociStitched	chr11	119856263	119877758	Rptoros
NR_045322	855	5_ _lociStitched	chr19	47507478	47532250	Gm19557
NR_045346	736	5_ _lociStitched	chr11	103346844	103360998	Arhgap27os3
NR_045384	569	5_ _lociStitched	chr5	64024685	64030505	5830416I19Rik
NR_045390	1118	10_ _lociStitched	chr18	73934882	73973203	D730045A05Rik
NR_045394	911	7_ _lociStitched	chr18	75277927	75288310	2010010A06Rik
NR_045435	664	5_ _lociStitched	chr17	86437682	86447786	2010106C02Rik
NR_045458	1032	7_ _lociStitched	chr17	83978015	84004374	4933433H22Rik
NR_045660	658	5_ _lociStitched	chr14	76524023	76533883	4930444M15Rik
NR_045729	113	12_ _lociStitched	chr2	33838087	33853966	Nron
NR_045733	41	18_ _lociStitched	chr6	120566736	120636262	1700072O05Rik
NR_045821	580	5_ _lociStitched	chr5	138257847	138264028	6330418K02Rik
NR_045838	1106	10_ _lociStitched	chr2	129213848	129244256	A730036I17Rik
NR_045844	176	11_ _lociStitched	chr12	25239994	25266400	Gm17746
NR_045844	674	5_ _lociStitched	chr12	25325715	25336144	Gm17746
NR_045844	831	5_ _lociStitched	chr12	25378366	25398973	Gm17746
NR_045844	695	5_ _lociStitched	chr12	25300771	25312471	Gm17746
NR_045844	349	9_ _lociStitched	chr12	25165002	25209703	Gm17746
NR_045871	844	5_ _lociStitched	chr5	107677706	107699637	4930428O21Rik
NR_045872	137	12_ _lociStitched	chr3	95966216	96001255	Gm9054
NR_045873	280	9_ _lociStitched	chr11	79075533	79093368	Gm11201
NR_045881	276	9_ _lociStitched	chr18	4907822	4925276	Gm10556
NR_045898	327	9_ _lociStitched	chr11	79640657	79669358	Rab11fip4os2

NR_045911	902	7_ _lociStitched	chr4	140944094	140953418	Gm13031
NR_046194	1011	7_ _lociStitched	chr11	85790572	85813139	Bcas3os2
NR_077219	354	16_ _lociStitched	chr16	91440834	91462247	A930006K02Rik
NM_001177394	967	7_ _lociStitched	chr2	45100599	45117607	Zeb2os
NM_001039472	598	5_ _lociStitched	chr1	136133675	136140751	Kif21b
NM_028770	450	8_ _lociStitched	chr15	101297931	101320808	krt80
NR_027640	27	24_ _lociStitched	chr1	156985494	157049639	Klkbl4
NM_054070	558	5_ _lociStitched	chr8	123450324	123455635	Afg3l1
