# Supplementary material for: Multi-omic integration of single-cell data uncovers methylation profiles of super-enhancers in skeletal muscle stem cells
Source: Epigenetics Chromatin. 2025 Aug 11;18:54. doi: 10.1186/s13072-025-00619-0 (PMC12337566; doi:10.1186/s13072-025-00619-0)
Supplement: Supplementary file 1 — Supplementary Material 1 [file 13072_2025_619_MOESM1_ESM.zip › Supplementary data/Supplementary Figure Legends.docx]

Supplementary Figure Legends:

Supplementary Figure 1: Clusters and their top five SEs. The top five SEs for each cluster were presented, ranked by their AUROC values to emphasize their regulatory significance. Each cluster was displayed separately, and the corresponding methylation profiles for the SEs were shown.

Supplementary Figure 2: Genes associated with the top five SEs. The top five genes linked to SEs for each cluster are displayed, alongside their corresponding methylation profiles. These profiles exhibit a strong consistency with the SEs.

Supplementary Figure 3: A heatmap displayed the marker peaks for regions showing differential accessibility within each cell type. B: MA scatter plots showed the differential peaks in chromatin open regions between aged and young individuals in each cell type. C: The top 3 ranked motifs in the aged group were NFYB, SP2, and PBX3, while in the young group, they were NR3C1, AR, and PGR, showing a clear difference in motif preferences between the two groups. D: 17 TFs were then re-evaluated specifically in MuSCs to compare differences between the aged and young group.

Supplementary Figure 4: A: CellChat analysis assessed the number and strength of interactions between cell types. B: The number and strength of interactions were presented specifically within MuSCs. C: A bubble plot illustrated that the majority of pathways were actively involved in intercellular communication between MuSCs and other cell types, emphasizing the key signaling interactions across various cellular networks.

Supplementary Figure 5: Methylation status of FCNB, GRAMD4, and EEA1 and their SEs. The methylation profiles of FCNB, GRAMD4, and EEA1 showed distinct patterns compared to their corresponding SEs, with all displaying upregulated methylation DMPs.
