# Supplementary material for: Multi-omic integration of single-cell data uncovers methylation profiles of super-enhancers in skeletal muscle stem cells
Source: Epigenetics Chromatin. 2025 Aug 11;18:54. doi: 10.1186/s13072-025-00619-0 (PMC12337566; doi:10.1186/s13072-025-00619-0)
Supplement: Supplementary file 1 — Supplementary Material 1 [file 13072_2025_619_MOESM1_ESM.zip › Supplementary data/Supplementary Table 2.pdf]

Supplementary Table 2. SEs in DMRs

SE-chrom	SE-start	SE-end	Lenth	SE-rank	RE_id	SE-related-GENE1	DMR-chrom	DMR-start	DMR-end
chr8	122273816	122331404	57588	19	31 lociStitched	Zfpm1	chr8	122305573	122305654
chr2	28063114	28100973	37859	32	18_ _lociStitched	Fcnb	chr2	28083204	28083463
chr4	32219489	32258130	38641	49	17 lociStitched	Bach2	chr4	32239184	32239216
chr5	124011422	124060542	49120	63	20 lociStitched	Vps37b	chr5	124045479	124045699
chr15	86050380	86071444	21064	75	14_ _lociStitched	Gramd4	chr15	86069499	86069744
chr4	133158830	133201399	42569	148	12_ _lociStitched	Gpr3	chr4	133195684	133195860
chr13	54205172	54223844	18672	157	11 lociStitched	Hrh2	chr13	54209706	54209768
chr6	120465376	120486428	21052	162	11 lociStitched	Il17ra	chr6	120470179	120470263
chr11	113536322	113563603	27281	178	11_ _lociStitched	Slc39a11	chr11	113540697	113540749
chr14	25458937	25489642	30705	190	11 lociStitched	Zmiz1	chr14	25461185	25461237
chr11	94987545	95013200	25655	225	13 lociStitched	Ppp1r9b	chr11	95011095	95011218
chr10	95765274	95781298	16024	270	9_ _lociStitched	Eea1	chr10	95776561	95776795
chr16	91520257	91539058	18801	284	9 lociStitched	Ifngr2	chr16	91526460	91526564
chr1	164066194	164087917	21723	297	9 lociStitched	Sell	chr1	164076674	164076827
chr16	91440834	91462247	21413	354	16_ _lociStitched	A930006K02Rik	chr16	91444667	91444847
chr19	44247679	44267139	19460	430	8 lociStitched	Scd2	chr19	44255587	44255605
chr17	31722260	31761376	39116	496	8 lociStitched	Cryaa	chr17	31730887	31731200
chr4	107742882	107783922	41040	498	8_ _lociStitched	Lrp8	chr4	107747019	107747265
chr15	82218653	82233040	14387	740	5 lociStitched	Tnfrsf13c	chr15	82225143	82225317
chr4	124695632	124710452	14820	752	5 lociStitched	Fhl3/Utp11	chr4	124696105	124696149
chr11	115899429	115929754	30325	864	5_ _lociStitched	Smim6	chr11	115903534	115903603
chr6	115968435	115973796	5361	869	7 lociStitched	Plxnd1	chr6	115970782	115970852
chr4	140944094	140953418	9324	902	7 lociStitched	Gm13031	chr4	140947102	140947143
chr5	65850727	65861048	10321	910	7_ _lociStitched	Rhoh	chr5	65855389	65855453
chr2	180728056	180740450	12394	927	7 lociStitched	Slc17a9	chr2	180733302	180733387
chr7	80444148	80457504	13356	938	7 lociStitched	Furin	chr7	80452856	80452992
chr10	79684609	79713158	28549	1041	7_ _lociStitched	Bsg	chr10	79690298	79690581
chr12	85624129	85661377	37248	1055	7_ _lociStitched	Jdp2	chr12	85646265	85646291
chr10	121411936	121430562	18626	1073	10 lociStitched	Rassf3	chr10	121422601	121422806
chr6	88037677	88075484	37807	1117	10 lociStitched	Rpn1	chr6	88061471	88061593
chr18	73934882	73973203	38321	1118	10_ _lociStitched	D730045A05Rik	chr18	73948387	73948472
chr12	110966020	111010638	44618	1123	10_ _lociStitched	6030440G07Rik	chr12	111005004	111005057
