# Supplementary figures and images for: Multi-omic integration of single-cell data uncovers methylation profiles of super-enhancers in skeletal muscle stem cells
Source: Epigenetics Chromatin. 2025 Aug 11;18:54. doi: 10.1186/s13072-025-00619-0 (PMC12337566; doi:10.1186/s13072-025-00619-0)

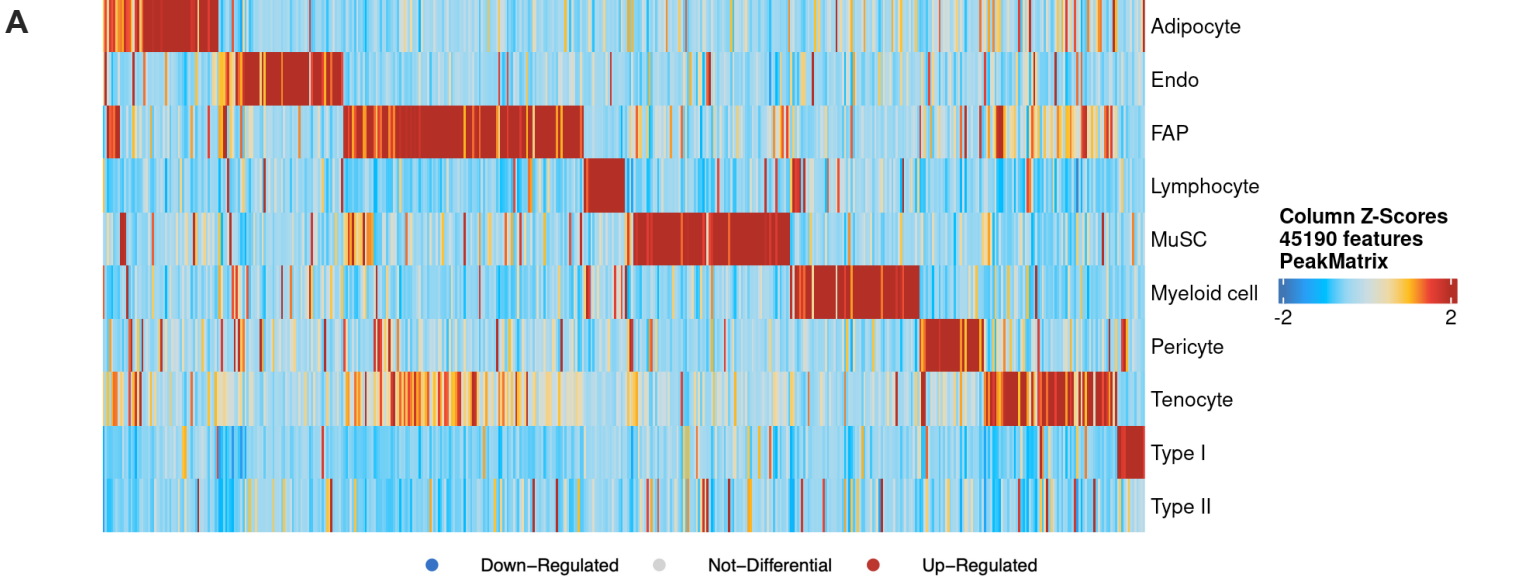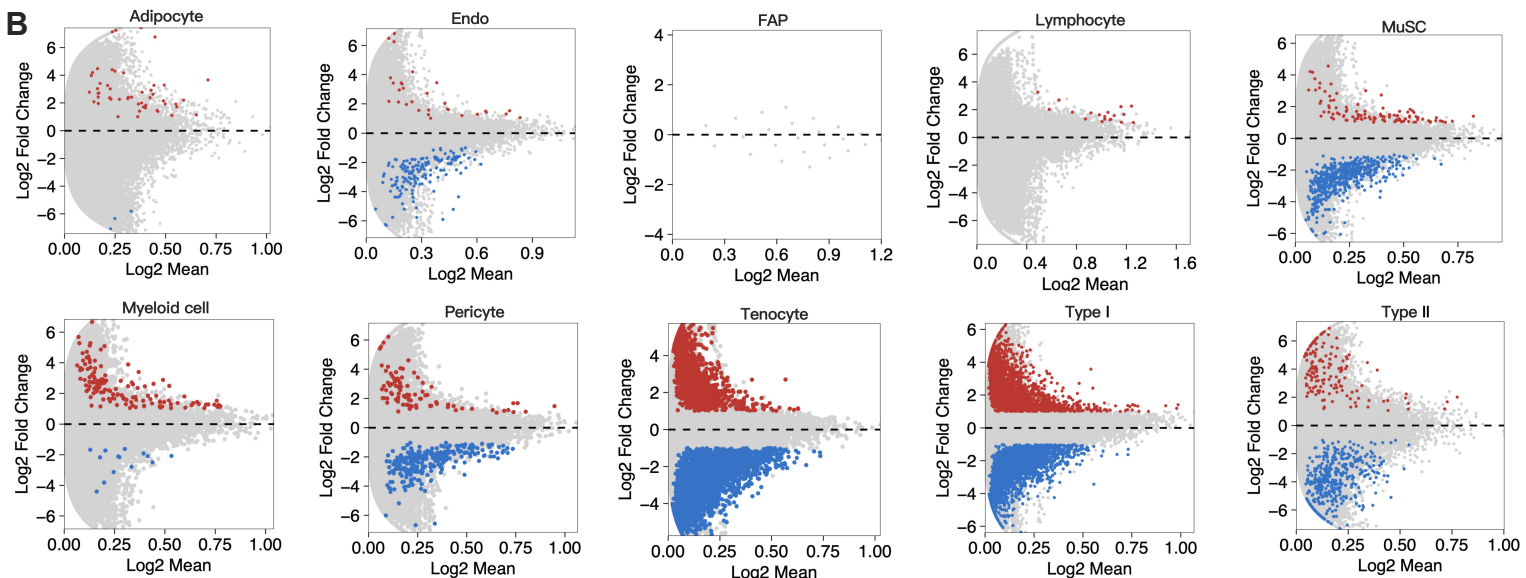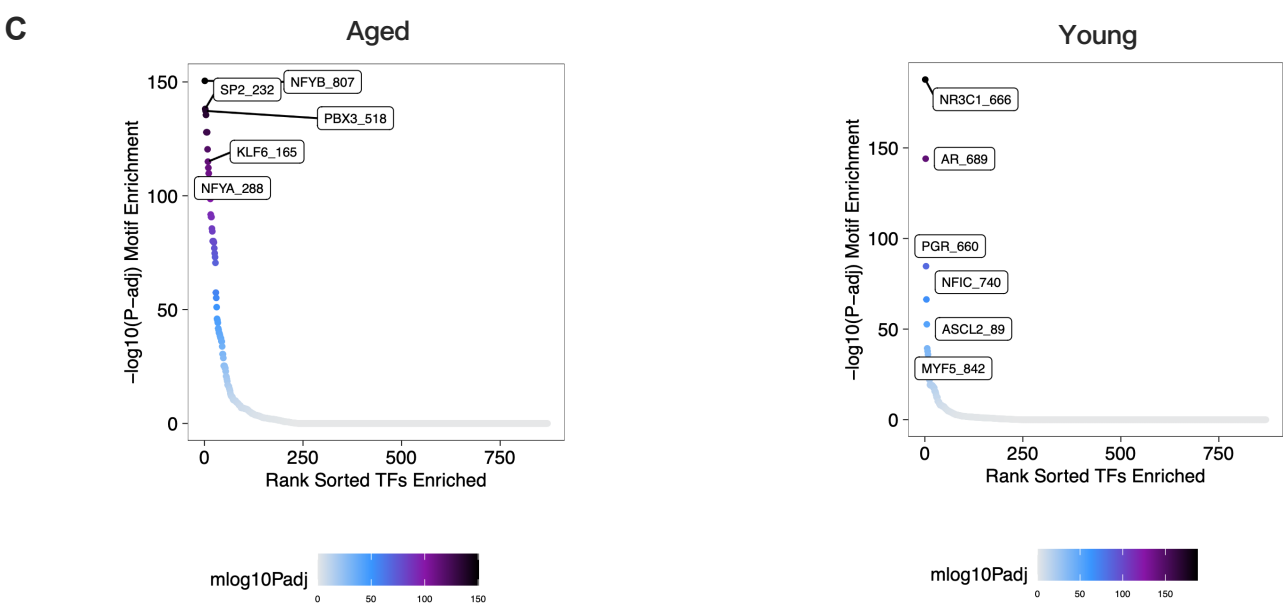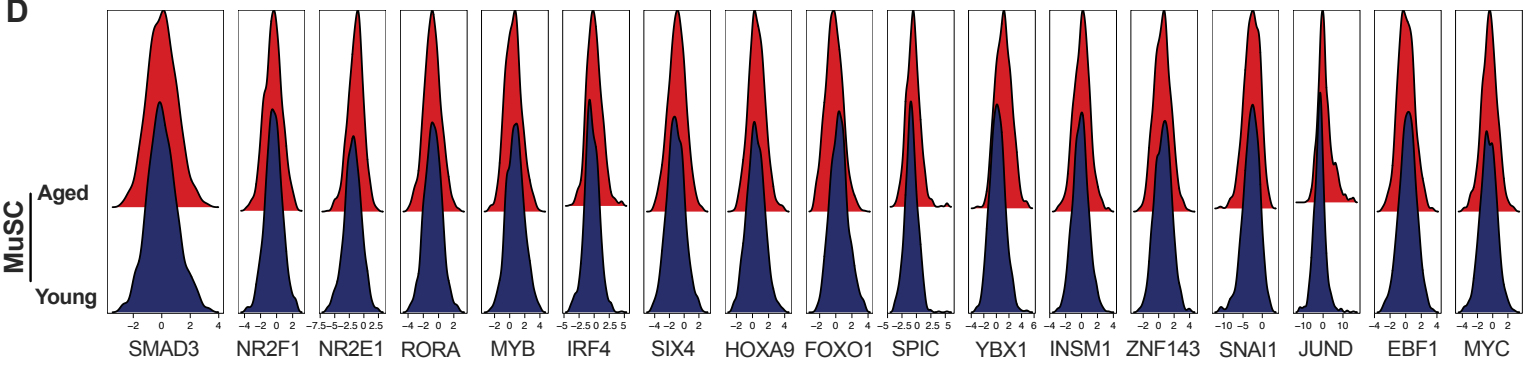

Supplement: Supplementary file 1 — Supplementary Material 1 [file 13072_2025_619_MOESM1_ESM.zip › Supplementary data/supplefig3.pdf]

# B

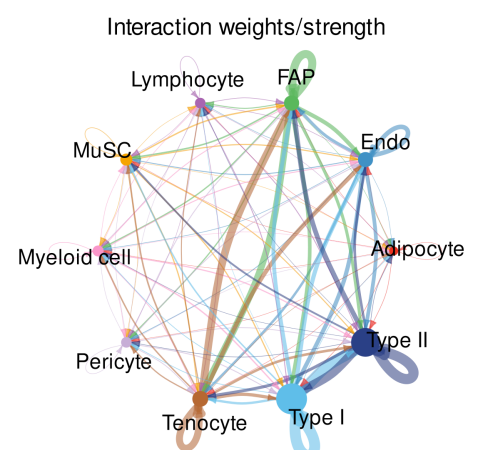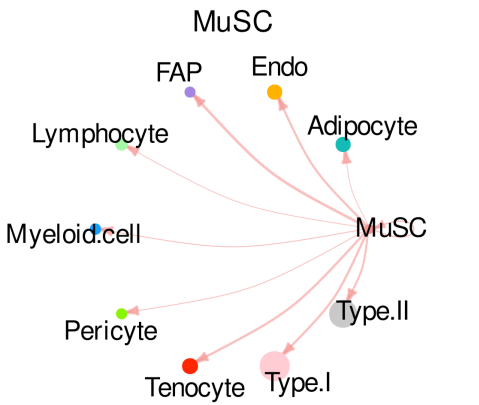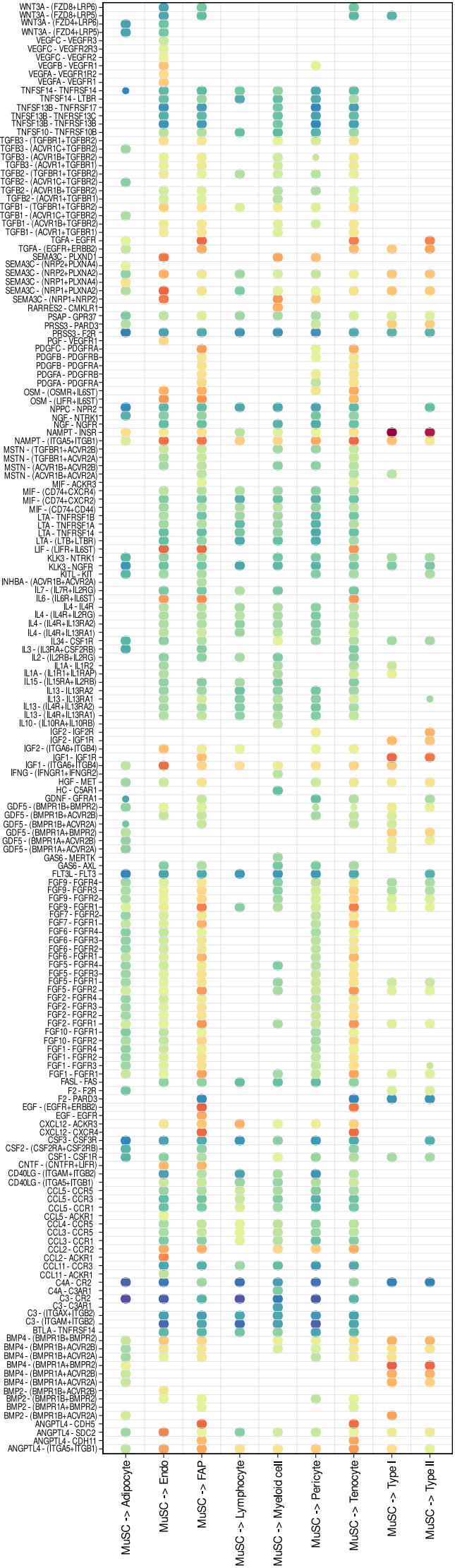

Supplement: Supplementary file 1 — Supplementary Material 1 [file 13072_2025_619_MOESM1_ESM.zip › Supplementary data/supplefig4.pdf]

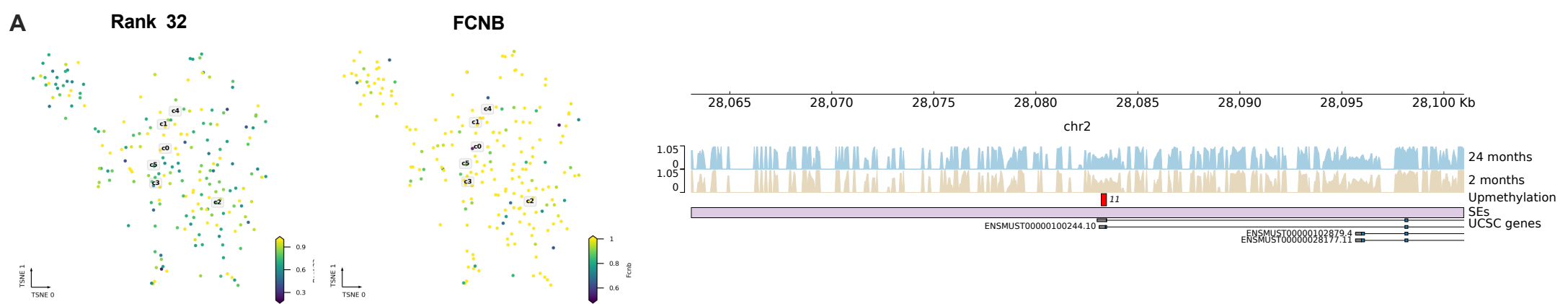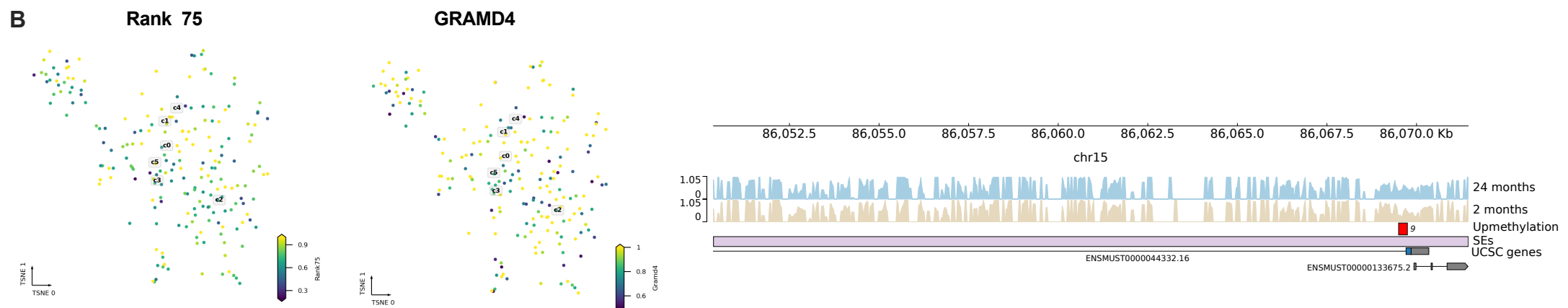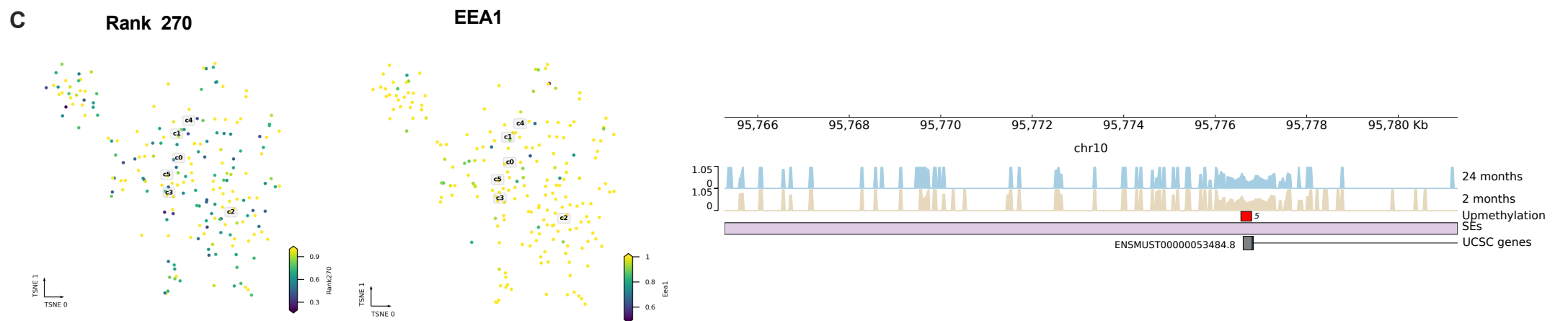

Supplement: Supplementary file 1 — Supplementary Material 1 [file 13072_2025_619_MOESM1_ESM.zip › Supplementary data/supplefig5.pdf]
